# Additional insights into the organization of transcriptional regulatory modules based on a 3D model of the *Saccharomyces cerevisiae* genome

**DOI:** 10.1186/s13104-022-05940-5

**Published:** 2022-02-19

**Authors:** Thibault Poinsignon, Mélina Gallopin, Jean-Michel Camadro, Pierre Poulain, Gaëlle Lelandais

**Affiliations:** 1 Institute for Integrative Biology of the Cell (I2BC), CEA, CNRS, Université Paris-Saclay, 91198 Gif-sur-Yvette, France; 2 Institut Jacques Monod, CNRS, Université de Paris, 75006 Paris, France

**Keywords:** Transcriptional regulations, Chromosome conformation capture, Yeast, 3D-Scere

## Abstract

**Objectives:**

Transcriptional regulatory modules are usually modelled via a network, in which nodes correspond to genes and edges correspond to regulatory associations between them. In the model yeast *Saccharomyces cerevisiae*, the topological properties of such a network are well-described (distribution of degrees, hierarchical levels, organization in network motifs, etc.). To go further on this, our aim was to search for additional information resulting from the new combination of classical representations of transcriptional regulatory networks with more realistic models of the spatial organization of *S. cerevisiae* genome in the nucleus.

**Results:**

Taking advantage of independent studies with high-quality datasets, i.e. lists of target genes for specific transcription factors and chromosome positions in a three dimensional space representing the nucleus, particular spatial co-localizations of genes that shared common regulatory mechanisms were searched. All transcriptional modules of *S. cerevisiae*, as described in the latest release of the YEASTRACT database were analyzed and significant biases toward co-localization for a few sets of target genes were observed. To help other researchers to reproduce such analysis with any list of genes of their interest, an interactive web tool called 3D-Scere (https://3d-scere.ijm.fr/) is provided.

**Supplementary Information:**

The online version contains supplementary material available at 10.1186/s13104-022-05940-5.

## Introduction

Normal cell functioning requires appropriate gene expression, which depends on multiple regulatory layers (see [1] for review). In this context, transcriptional regulatory modules (TRMs) were extensively studied (for instance [2–5]). By definition, a TRM is a set of genes for which transcriptional activity is modulated by a specific transcription factor (TF) [6]. In the model yeast *Saccharomyces cerevisiae*, TRMs are well described [2–5] and public databases like YEASTRACT [7] or SGD [8], provide lists of target genes for any TF. All together TRMs were explored to better understand their individual organizations, but also their collective relationships [4, 5, 9, 10]. In most studies, questions were addressed via a representation of TRMs as networks. In these networks, TF and target genes are the nodes, which are connected by directed edges (from TF to related targets). Topological properties of such networks were analysed to reveal the design principles underlying transcriptional regulations. It allowed the discovery of important regulatory motifs, surprisingly consistent across very different species [10, 11].

In addition to this information, spatial organization of the 16 chromosomes of *S. cerevisiae* was reported in the literature [1]. Experimental techniques derived from chromosome conformation capture (3C) were used to obtain a tridimensional (3D) model [12]. This model is based on the idea that interphase chromosomes are not positioned randomly within the nucleus. In particular, chromosomes should adopt a “Rabl configuration”, in which centromeres are clustered together at one pole of the nucleus, whereas arms are extended in several directions until telomeres, which are abutted to the nuclear envelope. Moreover, chromosome 12, which carries the rDNA repeats in *S. cerevisiae*, is expected to extend outward to join the nucleolus, i.e. the site of ribosome biogenesis (Additional file 1). This 3D model is relevant with the existence of a repressive chromatin structure, i.e. silent chromatin, which is known in yeasts for a long time (see [13] for a review) and affects mating-type loci, telomeres or rDNA repeats. More recently, this 3D model was used to study potential connections between interchromosomal DNA contacts and gene co-expressions [14]. Significant correlations were found, thus supporting the idea that a non-random nature of the genome organization helps to coordinate transcriptional processes in groups of genes, like those found in TRMs.

In this work, our aim was to search for additional insights into the organization of TRMs based on the 3D model of the *S. cerevisiae* genome at interphase. The TRMs were explored from a new perspective, which integrates functional and spatial information presently available, and addressed the following question: are target genes associated to a common TF (TRM) randomly disseminated within the nucleus, or are they preferentially co-localized? In the literature, this question was only partially answered, focusing essentially on spatial distances between genes coding for TFs and associated targets [15]. Our analysis represents an additional step in this context, reporting all distances between genes that belong to any TRM, as described in the latest release of the YEASTRACT database. Statistical parameters are provided, to quantify the intensity of potential bias observed in distributions of pairwise Euclidean distances calculated between lists of genes. A web tool called 3D-Scere (https://3d-scere.ijm.fr/) was also developed. With this tool, any researcher can retrieve information for all pairs of genes that belong to a list of his/her interest.

## Main text

This study extends on two previous analyses of transcriptional regulatory modules, recently presented in the literature. The first one is that of Monteiro et al*.* [2], published in 2020. The authors assessed the regulatory features of the current transcriptional network of *S. cerevisiae*, taking advantage of the latest release of their YEASTRACT database, which comprised almost 200,000 interactions including 220 TFs and 6886 target genes. The second one is that of Sun et al*.* [15], published in 2019. The authors used the 3D model of *S. cerevisiae* genome proposed by Duan et al*.* [12], to study the spatial organization of the regulatory network of *S. cerevisiae*. We propose that the perspectives and the data from the two studies can be elegantly combined to increase the scope of the results presented in each of them. Indeed, both studies have strengths, but also limitations. On one hand, the study of Monteiro et al*.* is based on a colossal work to collect, clean, and organize the transcriptional regulations identified in more than a thousand publications in peer-reviewed international journals. Notably, the authors also provided confidence level information for each regulation, thus delivering very high-quality data. They observed interesting topological properties of the global *S. cerevisiae* transcriptional network and discussed the complexity of the transcription regulatory processes that control gene expression. In that respect, searching for a potential role of genome organization in the functioning of this network, represents a natural perspective. On the other hand, Sun et al*.* had the original idea to place the transcriptional regulations between genes in the context of the 3D genome model available in *S. cerevisiae*. They concluded that “the transcriptional regulatory network of *S. cerevisiae* presents an optimized structure in space to adapt to functional requirements”. Undoubtedly very promising, we think that this conclusion (i) suffers from the use of transcriptional regulations, which were only partially verified and (ii) lacks individual analyses of TRMs.

**Fig. 1:**
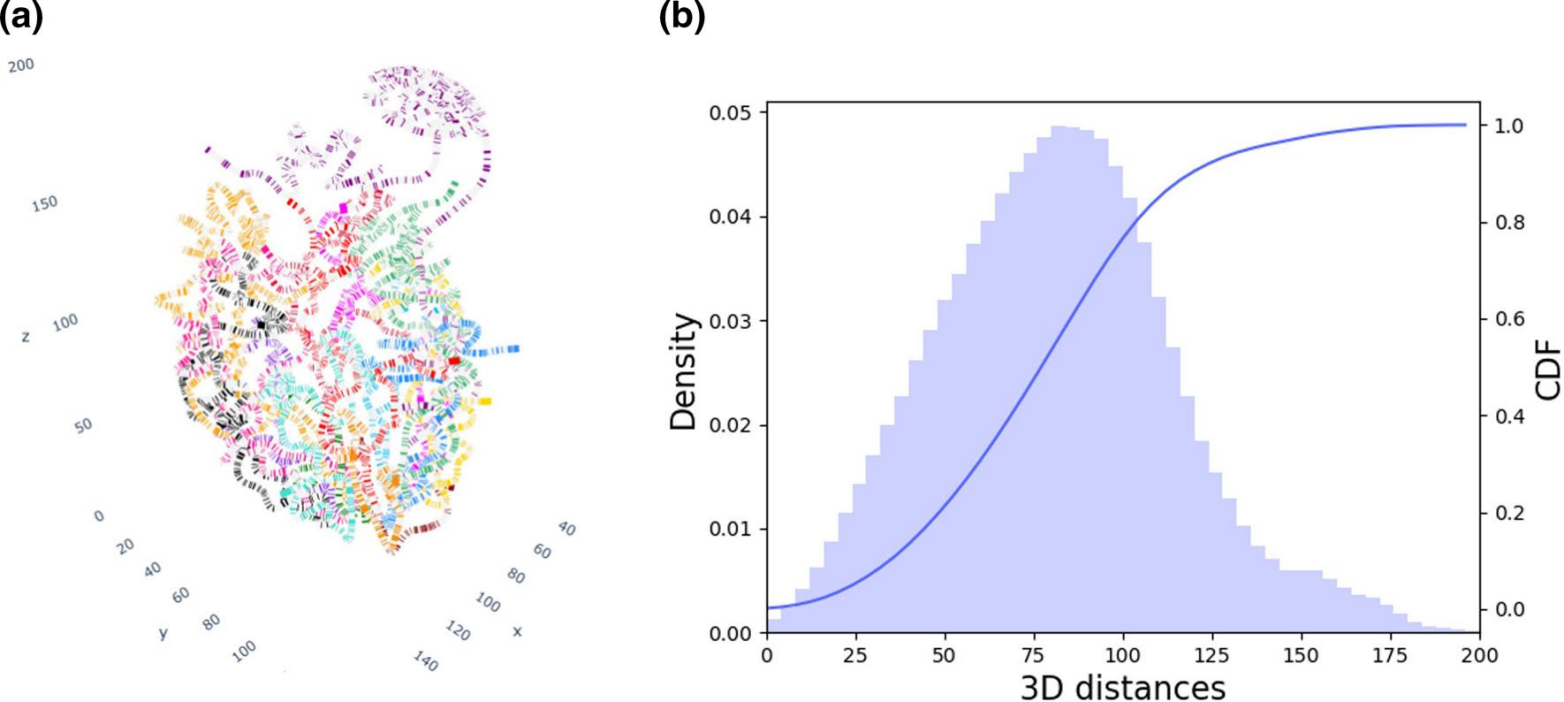
Spatial organization of the 16 chromosomes of *S. cerevisiae.* **a** Screenshot of the 3D genome model available in the 3D-Scere tool. **b** Density histogram (light blue) of all Euclidean distances between all chromosomal features in the 3D model (see “Main text”), and Cumulative Distribution Function (CDF, dark blue) of the density histogram

**Fig. 2:**
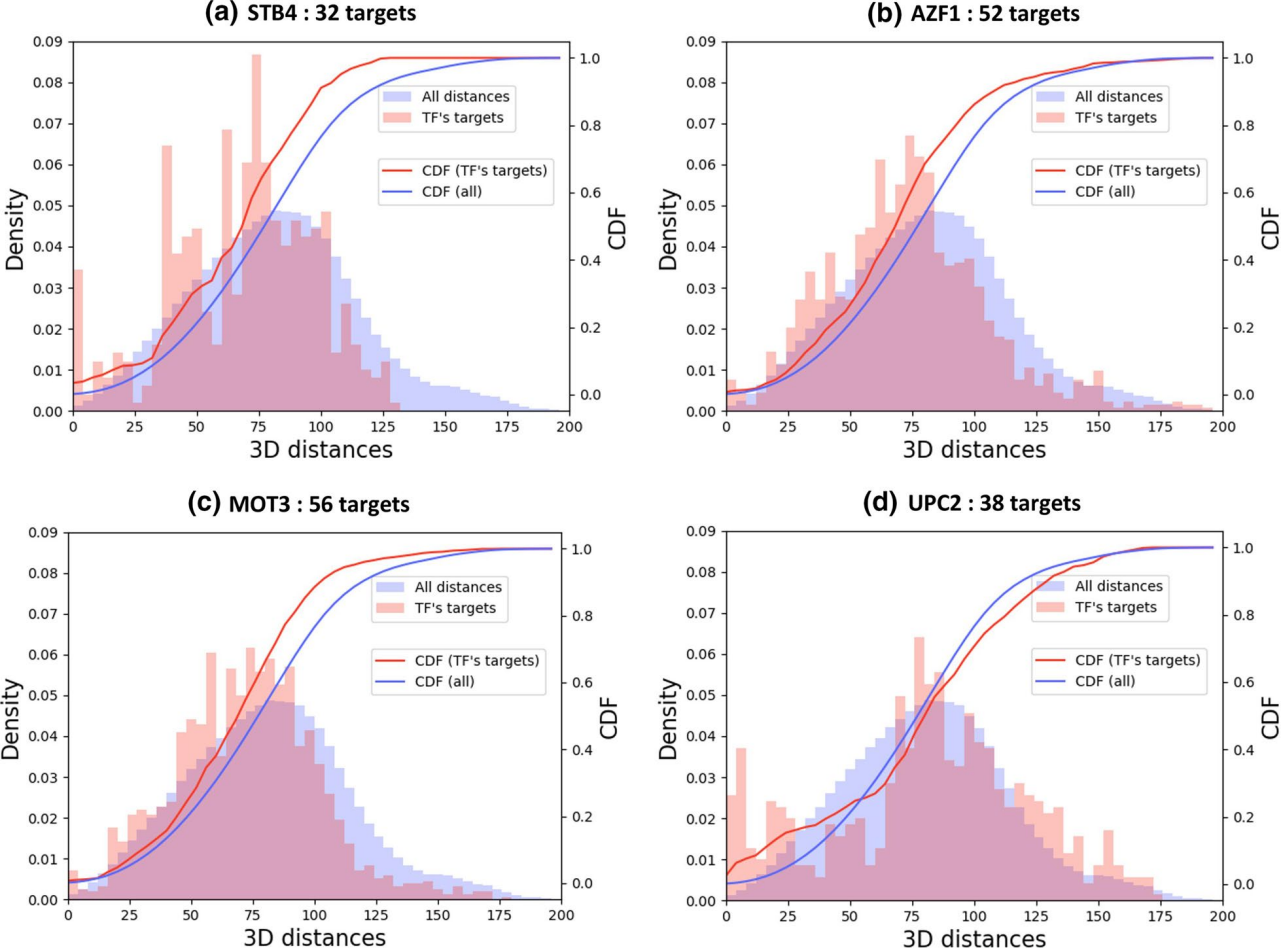
Examples of transcriptional regulatory modules in which targets are preferentially co-localized within the nucleus. Distance histograms (pink) for the targets of four TFs (STB4, AZF1, MOT3 and UPC2) are shown and compared to the distance histogram of all distances (light blue) as presented in Fig. 1. These TFs were selected because (i) they have a number of target genes > 30, (ii) they exhibit the highest values of Kolmogorov Smirnov statistics, with (iii) associated significant adjusted p-values (< 0.05)

The work presented in this article was performed in three steps. First, the TRMs were extracted from the study of Monteiro et al*.* [2]. The supplementary data provided all the YEASTRACT transcriptional regulations, annotated according to “binding evidence”, “expression evidence” or “both”. We decided to focus on regulatory associations which relied on “binding evidence” only. They represent 176 TFs with 6475 target genes, connected with 45,209 associations (23% of the full regulatory associations dataset). Second, the 3D model from the study of Duan et al*.* [12] was recovered. In the related supplementary data, the 3D coordinates for 26,538 “points” were found. Each point can be seen as a precise location in space, defined by 3D-coordinates (x, y, z). All together the points define all chromosomes of *S. cerevisiae* genome (Fig. 1a). Each chromosome was arranged into pairs of successive points, which thus delimit chromosomal regions in space. Note here that the obtained regions were of variable sizes because the points in the initial 3D model were not equidistant. We for instance observed that in situations where chromosomes are folded or change direction in space, more points were present to model the same length of DNA base pairs. Tridimensional coordinates for 9185 *S. cerevisiae* genome features (including 6572 ORFs) were next derived (Additional file 2) and used for calculations of spatial Euclidean distances between all pairs of genome features (this represents 42,177,520 distances) (Fig. 1b). All distance calculations are available as Additional file 3. For each TRM defined by the 176 different TFs, pairwise distances between target genes were selected. Distance distributions obtained with all features of the *S. cerevisiae* genome and with the subset of genes that belong to a particular TRM were finally superimposed and used to quantify a potential bias for co-localization (smaller distances) between target genes in TRMs. All results are available as Additional file 4. A Kolmogorov Smirnov (KS) test with a Bonferonni correction to quantify the deviation from the distribution of all genes was performed. As a result, several TFs for which the target genes exhibited atypical locations within the nucleus were observed. These TFs are listed in Table 1, and the distance distributions of the four TRMs with the highest KS statistic (i.e. highest deviation from the distribution of all targets) are shown in Fig. 2. An interesting situation, regarding the Upc2 transcriptional module, is detailed in Additional file 6.

**Table 1:** Statistical parameters derived from the study of the organization of transcriptional regulatory modules based on a 3D model of the *Saccharomyces cerevisiae* genome

TF	Description (SGD database)	# targets	KS value	p-val	p-val (adjusted)
STB4	Putative transcription factor; contains a Zn(II)2Cys6 zinc finger domain characteristic of DNA-binding proteins; computational analysis suggests a role in regulation of expression of genes encoding transporters; binds Sin3p in a two-hybrid assay	32	0.166	2e−12	3e−10
AZF1	Zinc-finger transcription factor; involved in diauxic shift; in the presence of glucose, activates transcription of genes involved in growth and carbon metabolism; in nonfermentable carbon sources, activates transcription of genes involved in maintenance of cell wall integrity; relocalizes to the cytosol in response to hypoxia	52	0.153	1e−27	1e−25
MOT3	Transcriptional repressor, activator; role in cellular adjustment to osmotic stress including modulation of mating efficiency; involved in repression of subset of hypoxic genes by Rox1p, repression of several DAN/TIR genes during aerobic growth, ergosterol biosynthetic genes in response to hyperosmotic stress; contributes to recruitment of Tup1p-Cyc8p general repressor to promoters; relocalizes to cytosol under hypoxia; forms [MOT3+] prion under anaerobic conditions	56	0.144	2e−28	5e−26
UPC2	Sterol regulatory element binding protein; induces sterol biosynthetic genes, upon sterol depletion; acts as a sterol sensor, binding ergosterol in sterol rich conditions; relocates from intracellular membranes to perinuclear foci upon sterol depletion; redundant activator of filamentation with ECM22, up-regulating the expression of filamentous growth genes; contains a Zn[2]-Cys[6] binuclear cluster; UPC2 has a paralog, ECM22, that arose from the whole genome duplication	38	0.106	2e−07	4e−05
PHO2	Homeobox transcription factor; regulatory targets include genes involved in phosphate metabolism; binds cooperatively with Pho4p to the PHO5 promoter; phosphorylation of Pho2p facilitates interaction with Pho4p; relocalizes to the cytosol in response to hypoxia	134	0.100	6e−78	1e−75
DAL80	Negative regulator of genes in multiple nitrogen degradation pathways; expression is regulated by nitrogen levels and by Gln3p; member of the GATA-binding family, forms homodimers and heterodimers with Gzf3p; DAL80 has a paralog, GZF3, that arose from the whole genome duplication	57	0.097	1e−13	2e−11
YAP3	Basic leucine zipper (bZIP) transcription factor	39	0.095	2e−06	0.0004
PLM2	Putative transcription factor, contains Forkhead Associated domain; found associated with chromatin; target of SBF transcription factor; induced in response to DNA damaging agents and deletion of telomerase; PLM2 has a paralog, TOS4, that arose from the whole genome duplication	182	0.093	5e−125	9e−123
RSF2	Zinc-finger protein; involved in transcriptional control of both nuclear and mitochondrial genes, many of which specify products required for glycerol-based growth, respiration, and other functions; RSF2 has a paralog, TDA9, that arose from the whole genome duplication; relocalizes from nucleus to cytoplasm upon DNA replication stress	35	0.092	7e−05	0.012
RPH1	JmjC domain-containing histone demethylase; targets tri- and dimethylated H3K36; associates with actively transcribed regions and promotes elongation; repressor of autophagy-related genes in nutrient-replete conditions; damage-responsive repressor of PHR1; phosphorylated by the Rad53p-dependent DNA damage checkpoint pathway and by a Rim1p-mediated event during starvation; target of stress-induced hormesis; RPH1 has a paralog, GIS1, that arose from the whole genome duplication	91	0.090	7e−30	1e−27

The ten TFs with the highest values of KS statistics are shown here. Results for all other TFs are available as Additional file 4

Finally, an open-source tool was developed, for interactive visualization and exploration. Source code is available on GitHub https://github.com/data-fun/3d-scere and the tool is freely usable online at https://3d-scere.ijm.fr/. It allows the visualization of any list of genes in the context of the 3D model of *S. cerevisiae* genome (Additional file 5 for screenshots). Further information can easily be added, like functional annotations (GO terms) or gene expression measurements. Qualitative or quantitative functional properties are highlighted in the large-scale 3D context of the genome with only a few mouse clicks.

## Limitations

We see in this work three main limitations. The first one concerns the biological relevance of the 3D model of the *S. cerevisiae* genome that was used. Created more than 10 years ago [12], this structural model represents only a static (and averaged) view of the relative positioning of the 16 chromosomes in the nucleus at interphase. It was obtained from 3C experiment data, which had to be processed with complex numerical procedures, to find an optimal solution. Because “optimal” does not guarantee “real”, all observations that emerge from this model must be further validated. In that respect, new data generated with the latest and most powerful Hi-C techniques, at different stages of the *S. cerevisiae* cell cycle to capture the dynamics of its genome organization could be of great interest. The second limitation concerns the lack of landmarks for the localization of genes, within the nucleus. Are they located near the nuclear envelope and possibly near pores allowing, for instance, the rapid export of transcripts to the cytoplasm? Such information is presently missing from our analyses. One solution could be to calculate additional distances with referential points on chromosomes such as centromeres, telomeres, or the outside emblematic region of rDNA repeats. Finally, the third limitation, in our point of view, relies on the definition of TRMs by themself. We defined a TRM as a set of genes for which the expression is modulated by a common TF. In this work, we reasoned by individual TRM. But a target gene can belong to several TRMs and also can require, to be transcriptionally regulated, the association between several TFs. Such genes could be studied specifically for particular co-localizations on the 3D model of the *S. cerevisiae* genome. Our strategy thus opens interesting research perspectives in the context of the study of gene lists that belong to transcriptional modules, but it can be of interest for any list of genes. The spatial proximity could be studied, between strongly (or weakly) expressed genes, or between genes which encode proteins involved in common metabolic pathways or which associate within complexes, etc. In this context, the online tool (https://3d-scere.ijm.fr/) will be of interest to the community, allowing any researcher to query any list of genes for which he/she has a particular interest in.

## Supplementary Information

**Additional file 1.** Pictures of the 3D model of the S. cerevisiae genome, as it is available in the 3D-Scere tool.

**Additional file 2.** Text file describing the method that was applied to associate the 9185 *S. cerevisiae* chromosomal features with the spatial coordinates of the 3D model.

**Additional file 3.** ZIP file with distance calculations between all pairwise chromosomal features for which spatial coordinates were associated to the 3D model (link from Zenodo repository: https://zenodo.org/record/5841177/files/3D_distances.parquet.gzip?download=1).

**Additional file 4.** ZIP file with graphical representations associated to each transcriptional module: (link from Zenodo repository: https://zenodo.org/record/5841177/files/supplementary-data-file-S4.zip?download=1).

**Additional file 5.** General overview of the 3d-Scere tool. A public web acces is available at https://3d-scere.ijm.fr. Three different uses are proposed to users: (1) «GO term projection», (2) Quantitative variable projection and (3) 3D distances histogram and network. Each access starts with the upload of a list of genes of interest for the user. Note that the list of Upc2 targets can be loaded as a «demo data». From the list of genes, users can manipulate either qualitative information (Access 1) or quantitative information (Access 2) and obtain graphics showing location on the chromosome or location on the 3D model of S. cerevisiae genome. Distribution of pairwise distances between genes is obtain with the Access 3.

**Additional file 6. **New insights into the transcriptional module related to Upc2 transcription factor.

## Data Availability

Source code written to generate figures is available on GitHub: https://github.com/data-fun/3d-scere-scripts. Source code written to develop the web tool (3d-Scere) is also available on GitHub: https://github.com/data-fun/3d-scere. Results are archived in the Zenodo repository: https://zenodo.org/record/5841177.

## Abbreviations

TRM: Transcriptional regulatory module
TF: Transcription factor
3C: Chromosome conformation capture
ORF: Open reading frame
GO: Gene ontology
Scere: *Saccharomyces cerevisiae*
SGD: Saccharomyces genome database
