# Supplementary figures and images for: Additional insights into the organization of transcriptional regulatory modules based on a 3D model of the Saccharomyces cerevisiae genome (part 1 of 2)
Source: BMC Res Notes. 2022 Feb 19;15:67. doi: 10.1186/s13104-022-05940-5 (PMC8858486; doi:10.1186/s13104-022-05940-5)

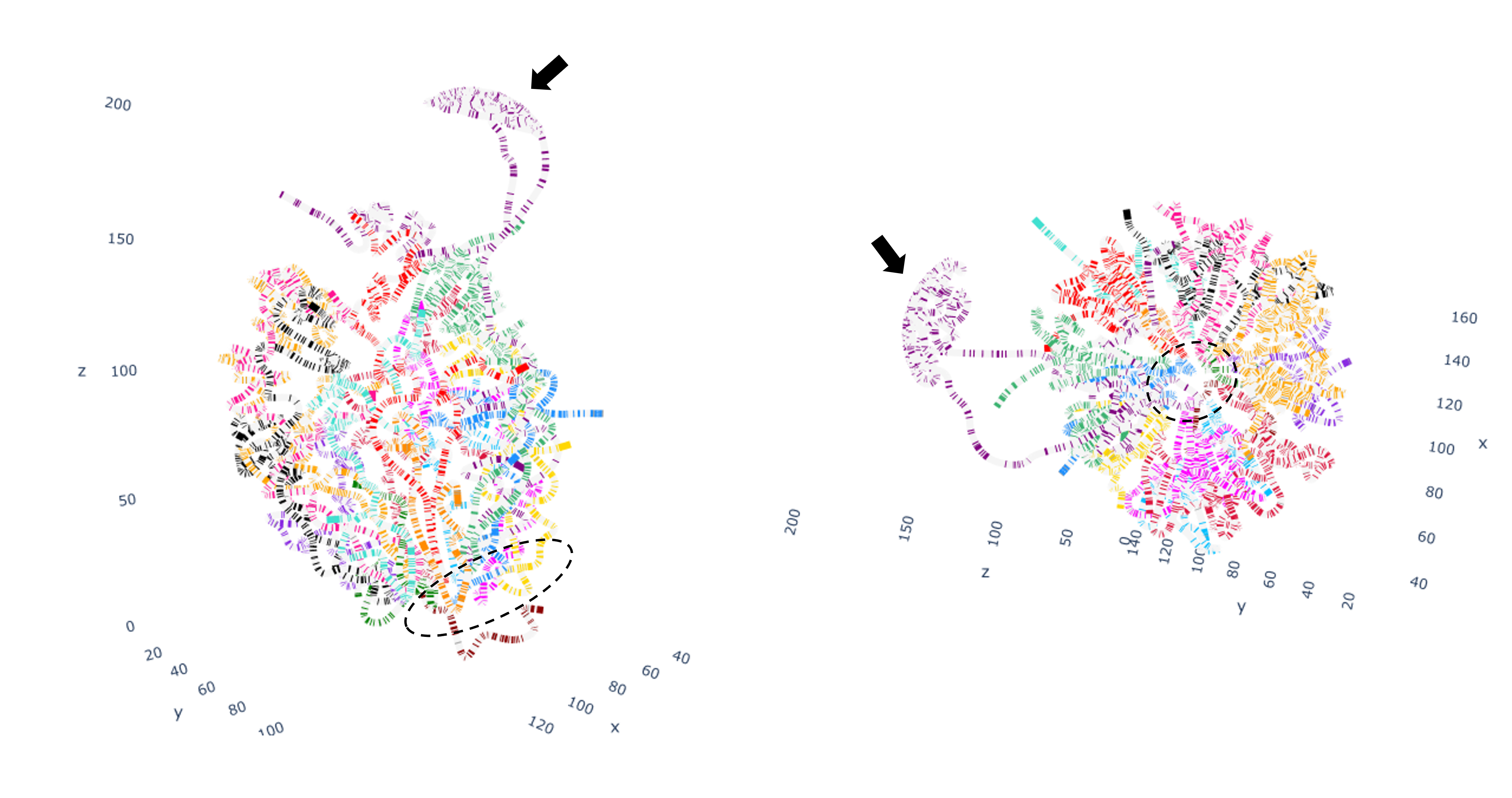

Supplement: Supplementary file 1 — Additional file 1. Pictures of the 3D model of the S. cerevisiae genome, as it is available in the 3D-Scere tool. [file 13104_2022_5940_MOESM1_ESM.png]

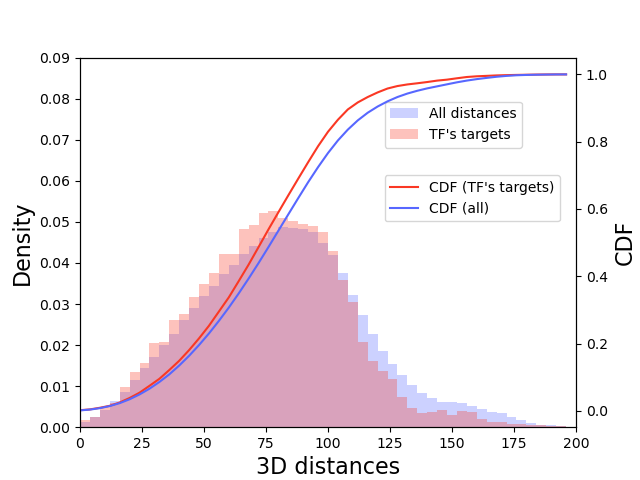

Supplement: Supplementary file 4 — Additional file 4. ZIP file with graphical representations associated to each transcriptional module: (link from Zenodo repository: https://zenodo.org/record/5841177/files/supplementary-data-file-S4.zip?download=1). [file 13104_2022_5940_MOESM4_ESM.zip › 3D_distances_distribution/TOS4_199_targets.csv.png]

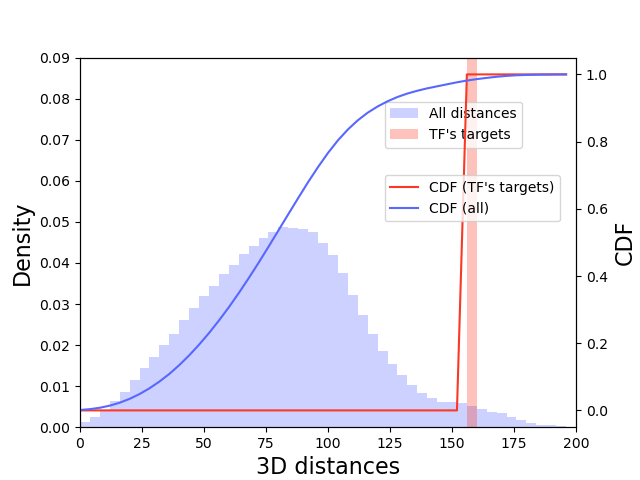

Supplement: Supplementary file 4 — Additional file 4. ZIP file with graphical representations associated to each transcriptional module: (link from Zenodo repository: https://zenodo.org/record/5841177/files/supplementary-data-file-S4.zip?download=1). [file 13104_2022_5940_MOESM4_ESM.zip › 3D_distances_distribution/MAL13_2_targets.csv.png]

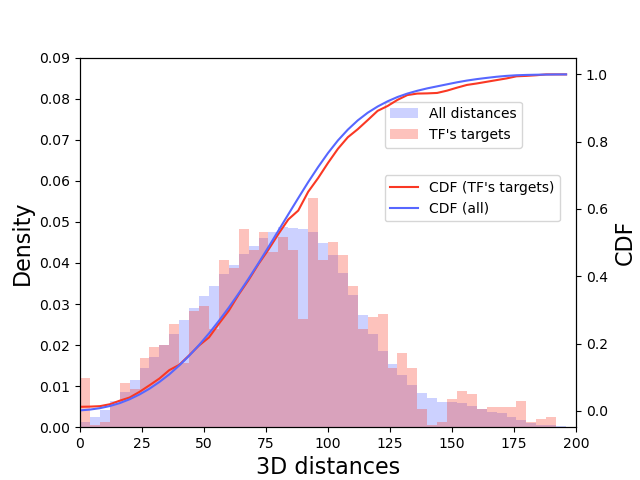

Supplement: Supplementary file 4 — Additional file 4. ZIP file with graphical representations associated to each transcriptional module: (link from Zenodo repository: https://zenodo.org/record/5841177/files/supplementary-data-file-S4.zip?download=1). [file 13104_2022_5940_MOESM4_ESM.zip › 3D_distances_distribution/MET4_57_targets.csv.png]

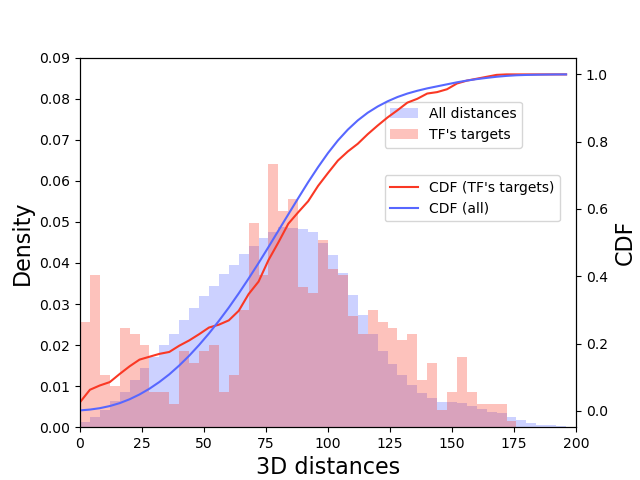

Supplement: Supplementary file 4 — Additional file 4. ZIP file with graphical representations associated to each transcriptional module: (link from Zenodo repository: https://zenodo.org/record/5841177/files/supplementary-data-file-S4.zip?download=1). [file 13104_2022_5940_MOESM4_ESM.zip › 3D_distances_distribution/UPC2_38_targets.csv.png]

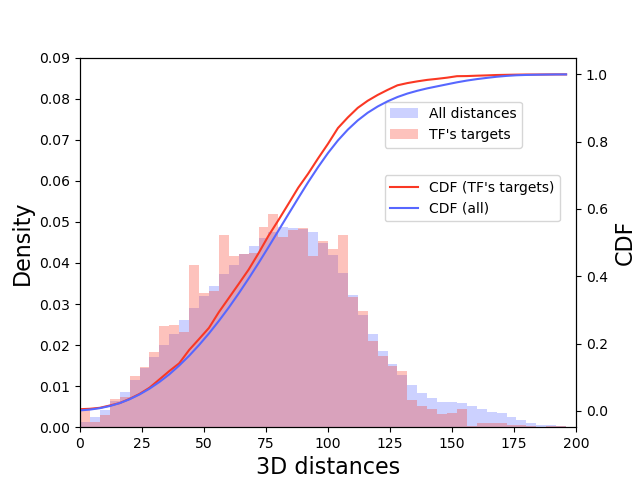

Supplement: Supplementary file 4 — Additional file 4. ZIP file with graphical representations associated to each transcriptional module: (link from Zenodo repository: https://zenodo.org/record/5841177/files/supplementary-data-file-S4.zip?download=1). [file 13104_2022_5940_MOESM4_ESM.zip › 3D_distances_distribution/UGA3_105_targets.csv.png]

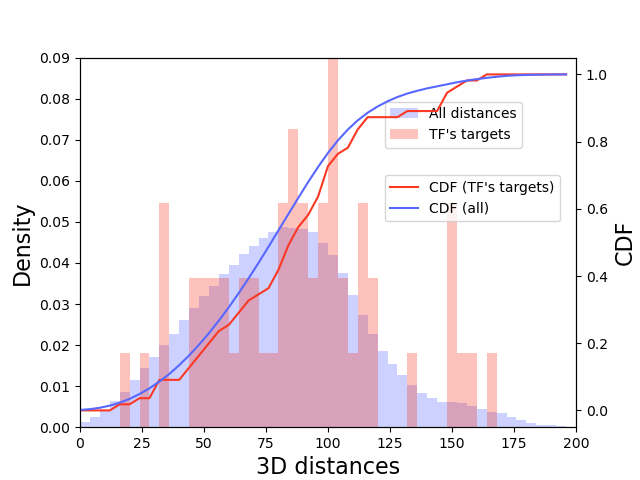

Supplement: Supplementary file 4 — Additional file 4. ZIP file with graphical representations associated to each transcriptional module: (link from Zenodo repository: https://zenodo.org/record/5841177/files/supplementary-data-file-S4.zip?download=1). [file 13104_2022_5940_MOESM4_ESM.zip › 3D_distances_distribution/PDC2_11_targets.csv.png]

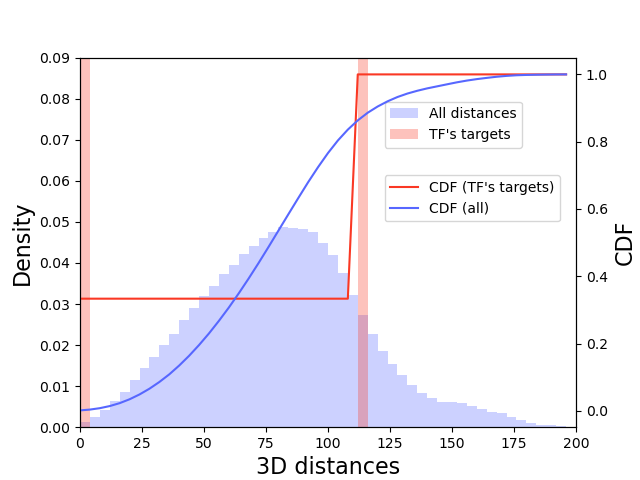

Supplement: Supplementary file 4 — Additional file 4. ZIP file with graphical representations associated to each transcriptional module: (link from Zenodo repository: https://zenodo.org/record/5841177/files/supplementary-data-file-S4.zip?download=1). [file 13104_2022_5940_MOESM4_ESM.zip › 3D_distances_distribution/GAL80_3_targets.csv.png]

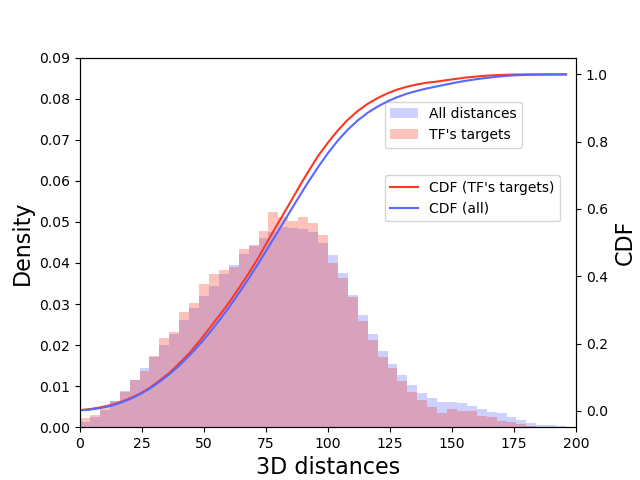

Supplement: Supplementary file 4 — Additional file 4. ZIP file with graphical representations associated to each transcriptional module: (link from Zenodo repository: https://zenodo.org/record/5841177/files/supplementary-data-file-S4.zip?download=1). [file 13104_2022_5940_MOESM4_ESM.zip › 3D_distances_distribution/RFX1_381_targets.csv.png]

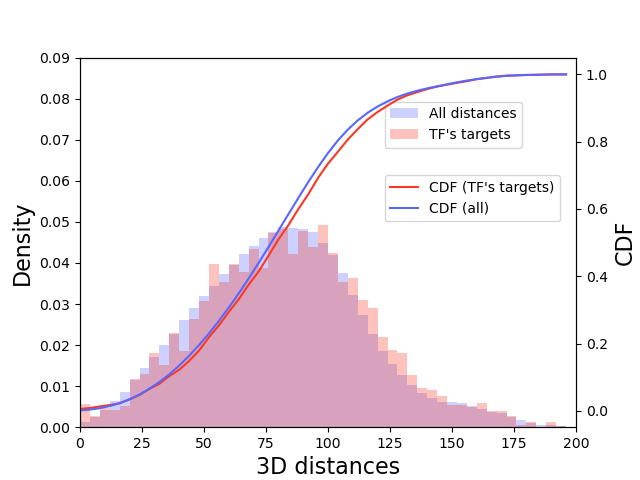

Supplement: Supplementary file 4 — Additional file 4. ZIP file with graphical representations associated to each transcriptional module: (link from Zenodo repository: https://zenodo.org/record/5841177/files/supplementary-data-file-S4.zip?download=1). [file 13104_2022_5940_MOESM4_ESM.zip › 3D_distances_distribution/HAP3_159_targets.csv.png]

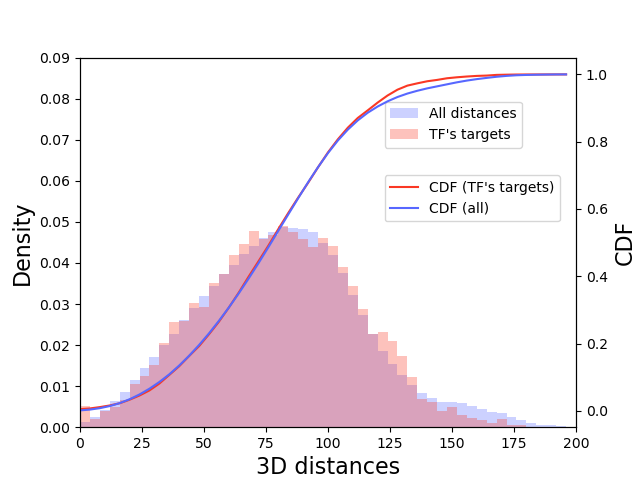

Supplement: Supplementary file 4 — Additional file 4. ZIP file with graphical representations associated to each transcriptional module: (link from Zenodo repository: https://zenodo.org/record/5841177/files/supplementary-data-file-S4.zip?download=1). [file 13104_2022_5940_MOESM4_ESM.zip › 3D_distances_distribution/PDR1_198_targets.csv.png]

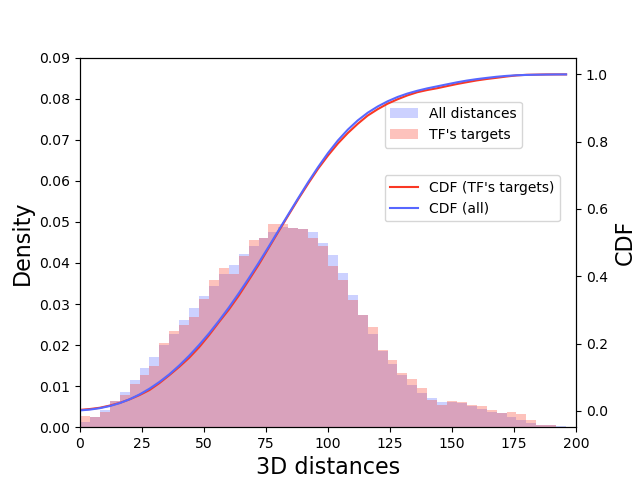

Supplement: Supplementary file 4 — Additional file 4. ZIP file with graphical representations associated to each transcriptional module: (link from Zenodo repository: https://zenodo.org/record/5841177/files/supplementary-data-file-S4.zip?download=1). [file 13104_2022_5940_MOESM4_ESM.zip › 3D_distances_distribution/TBF1_501_targets.csv.png]

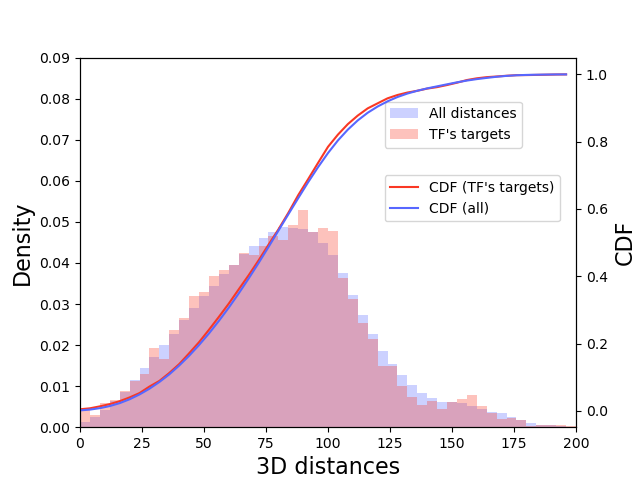

Supplement: Supplementary file 4 — Additional file 4. ZIP file with graphical representations associated to each transcriptional module: (link from Zenodo repository: https://zenodo.org/record/5841177/files/supplementary-data-file-S4.zip?download=1). [file 13104_2022_5940_MOESM4_ESM.zip › 3D_distances_distribution/YHP1_201_targets.csv.png]

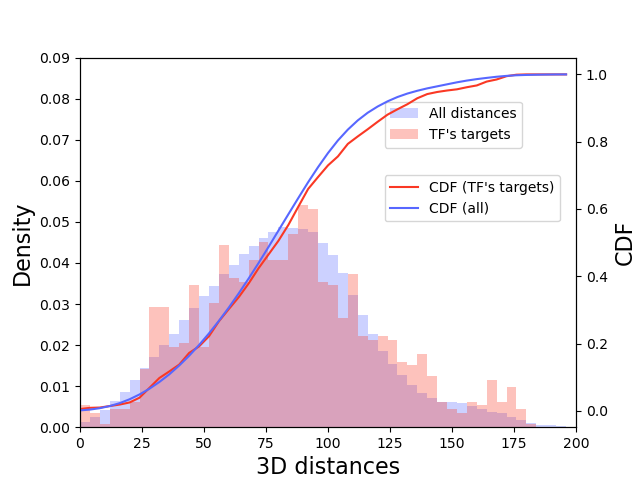

Supplement: Supplementary file 4 — Additional file 4. ZIP file with graphical representations associated to each transcriptional module: (link from Zenodo repository: https://zenodo.org/record/5841177/files/supplementary-data-file-S4.zip?download=1). [file 13104_2022_5940_MOESM4_ESM.zip › 3D_distances_distribution/CAT8_48_targets.csv.png]

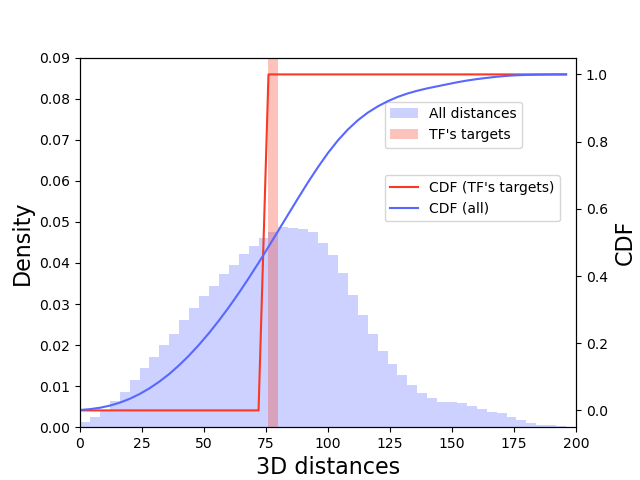

Supplement: Supplementary file 4 — Additional file 4. ZIP file with graphical representations associated to each transcriptional module: (link from Zenodo repository: https://zenodo.org/record/5841177/files/supplementary-data-file-S4.zip?download=1). [file 13104_2022_5940_MOESM4_ESM.zip › 3D_distances_distribution/ACA1_2_targets.csv.png]

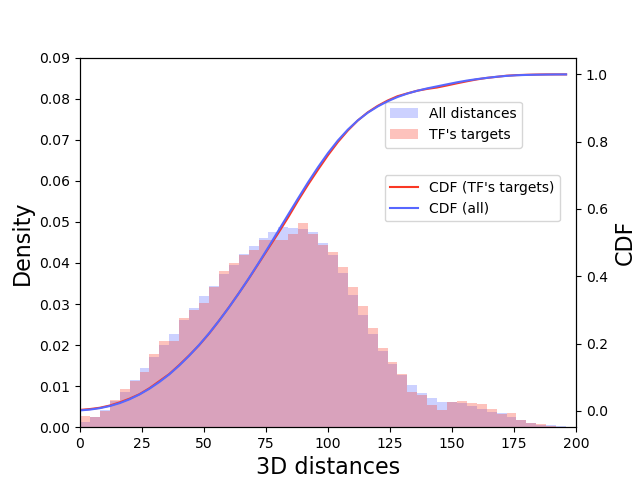

Supplement: Supplementary file 4 — Additional file 4. ZIP file with graphical representations associated to each transcriptional module: (link from Zenodo repository: https://zenodo.org/record/5841177/files/supplementary-data-file-S4.zip?download=1). [file 13104_2022_5940_MOESM4_ESM.zip › 3D_distances_distribution/MET32_401_targets.csv.png]

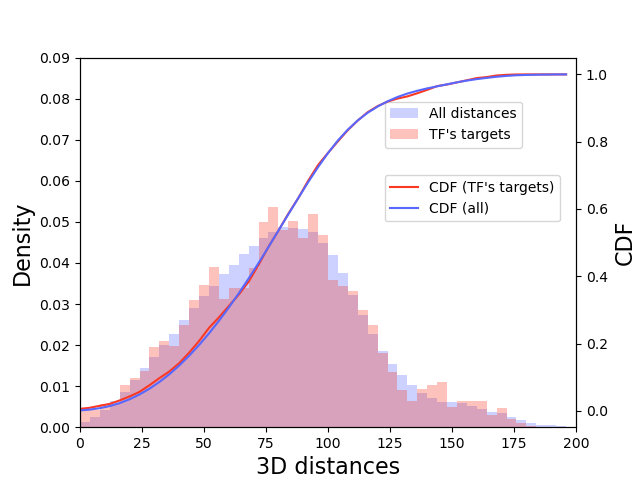

Supplement: Supplementary file 4 — Additional file 4. ZIP file with graphical representations associated to each transcriptional module: (link from Zenodo repository: https://zenodo.org/record/5841177/files/supplementary-data-file-S4.zip?download=1). [file 13104_2022_5940_MOESM4_ESM.zip › 3D_distances_distribution/HAL9_106_targets.csv.png]

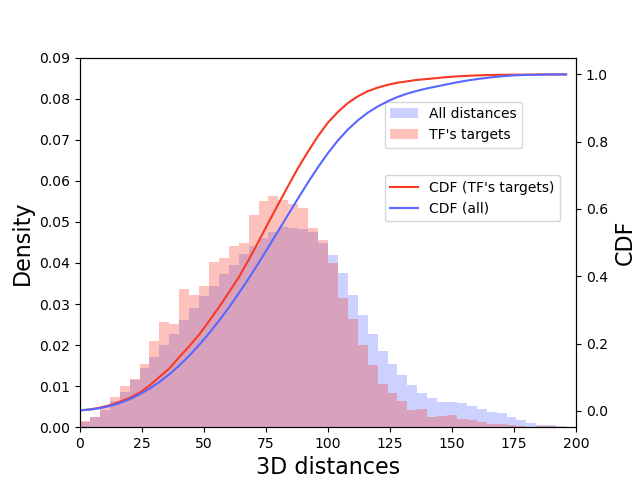

Supplement: Supplementary file 4 — Additional file 4. ZIP file with graphical representations associated to each transcriptional module: (link from Zenodo repository: https://zenodo.org/record/5841177/files/supplementary-data-file-S4.zip?download=1). [file 13104_2022_5940_MOESM4_ESM.zip › 3D_distances_distribution/PLM2_182_targets.csv.png]

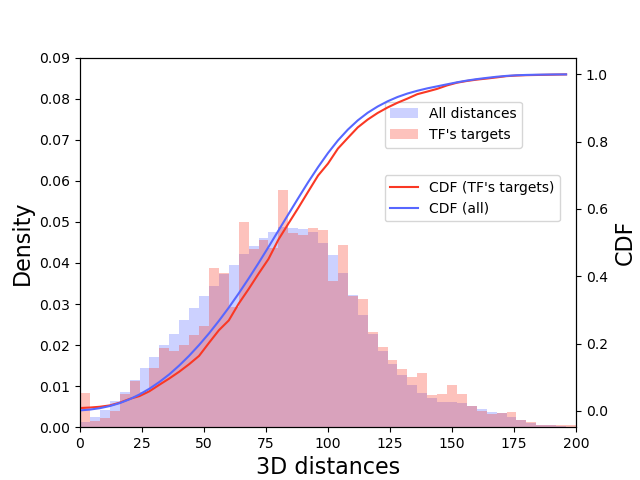

Supplement: Supplementary file 4 — Additional file 4. ZIP file with graphical representations associated to each transcriptional module: (link from Zenodo repository: https://zenodo.org/record/5841177/files/supplementary-data-file-S4.zip?download=1). [file 13104_2022_5940_MOESM4_ESM.zip › 3D_distances_distribution/RME1_85_targets.csv.png]

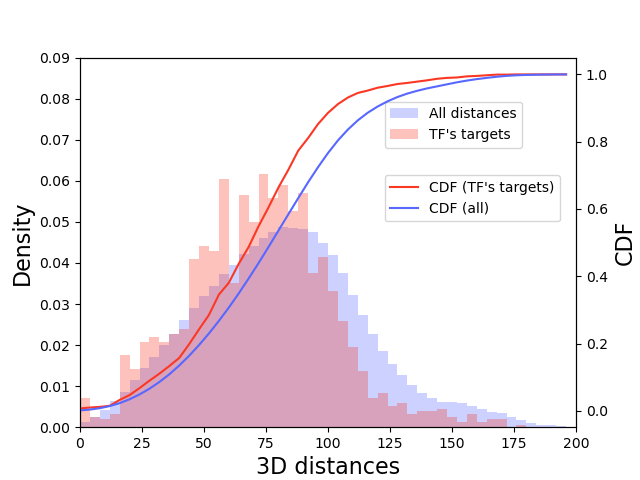

Supplement: Supplementary file 4 — Additional file 4. ZIP file with graphical representations associated to each transcriptional module: (link from Zenodo repository: https://zenodo.org/record/5841177/files/supplementary-data-file-S4.zip?download=1). [file 13104_2022_5940_MOESM4_ESM.zip › 3D_distances_distribution/MOT3_56_targets.csv.png]

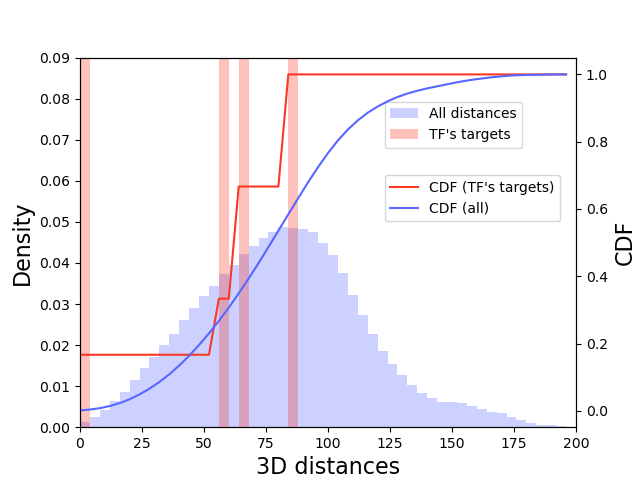

Supplement: Supplementary file 4 — Additional file 4. ZIP file with graphical representations associated to each transcriptional module: (link from Zenodo repository: https://zenodo.org/record/5841177/files/supplementary-data-file-S4.zip?download=1). [file 13104_2022_5940_MOESM4_ESM.zip › 3D_distances_distribution/YPR196W_4_targets.csv.png]

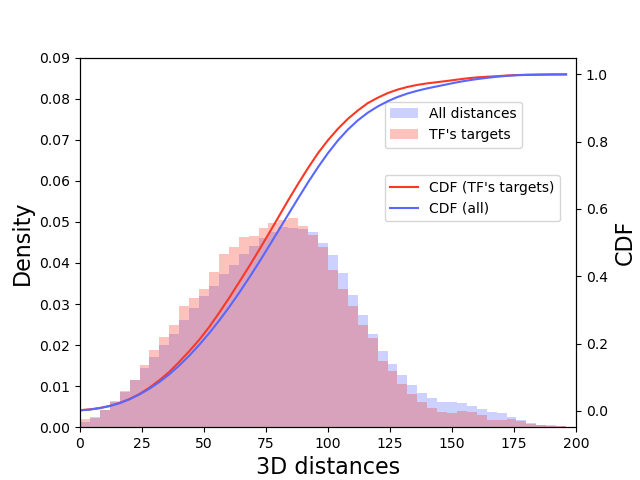

Supplement: Supplementary file 4 — Additional file 4. ZIP file with graphical representations associated to each transcriptional module: (link from Zenodo repository: https://zenodo.org/record/5841177/files/supplementary-data-file-S4.zip?download=1). [file 13104_2022_5940_MOESM4_ESM.zip › 3D_distances_distribution/SOK2_863_targets.csv.png]

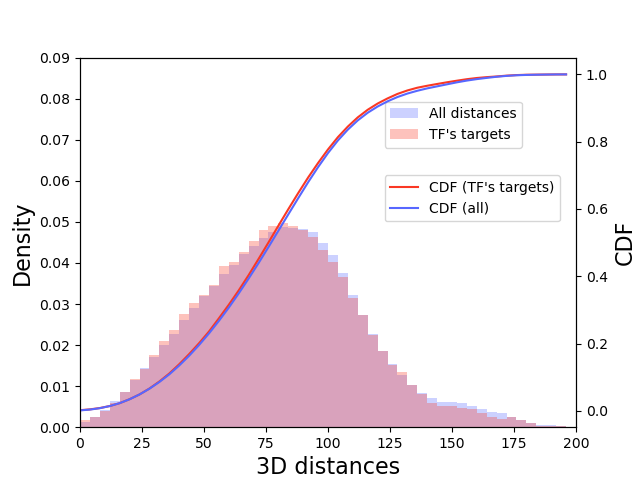

Supplement: Supplementary file 4 — Additional file 4. ZIP file with graphical representations associated to each transcriptional module: (link from Zenodo repository: https://zenodo.org/record/5841177/files/supplementary-data-file-S4.zip?download=1). [file 13104_2022_5940_MOESM4_ESM.zip › 3D_distances_distribution/MSN4_1241_targets.csv.png]

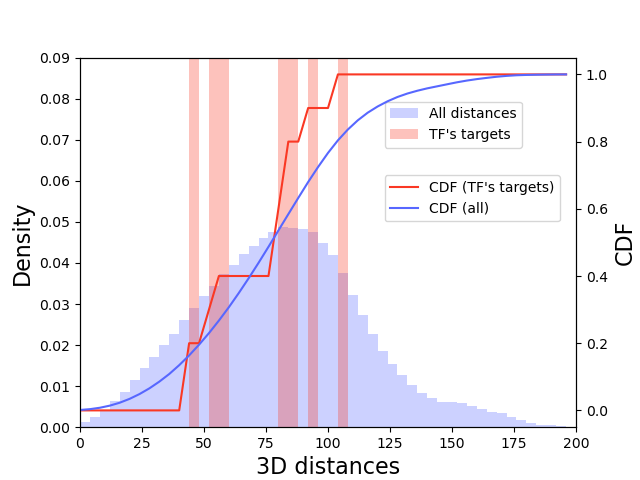

Supplement: Supplementary file 4 — Additional file 4. ZIP file with graphical representations associated to each transcriptional module: (link from Zenodo repository: https://zenodo.org/record/5841177/files/supplementary-data-file-S4.zip?download=1). [file 13104_2022_5940_MOESM4_ESM.zip › 3D_distances_distribution/OPI1_5_targets.csv.png]

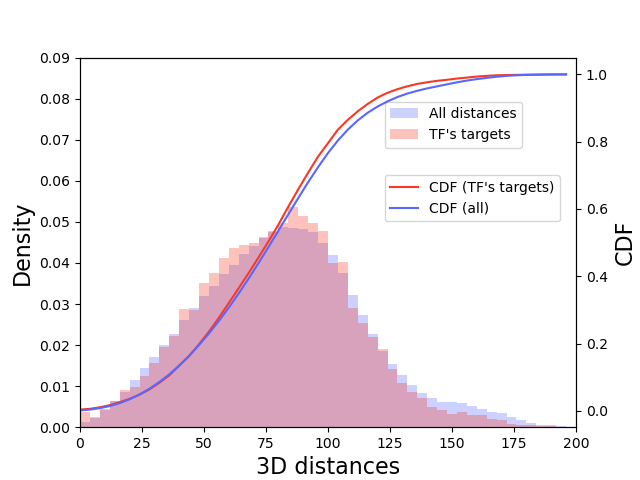

Supplement: Supplementary file 4 — Additional file 4. ZIP file with graphical representations associated to each transcriptional module: (link from Zenodo repository: https://zenodo.org/record/5841177/files/supplementary-data-file-S4.zip?download=1). [file 13104_2022_5940_MOESM4_ESM.zip › 3D_distances_distribution/MBP1_373_targets.csv.png]

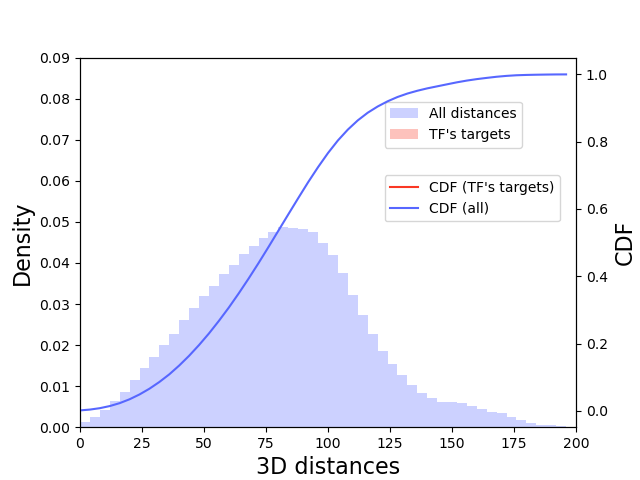

Supplement: Supplementary file 4 — Additional file 4. ZIP file with graphical representations associated to each transcriptional module: (link from Zenodo repository: https://zenodo.org/record/5841177/files/supplementary-data-file-S4.zip?download=1). [file 13104_2022_5940_MOESM4_ESM.zip › 3D_distances_distribution/MTH1_1_targets.csv.png]

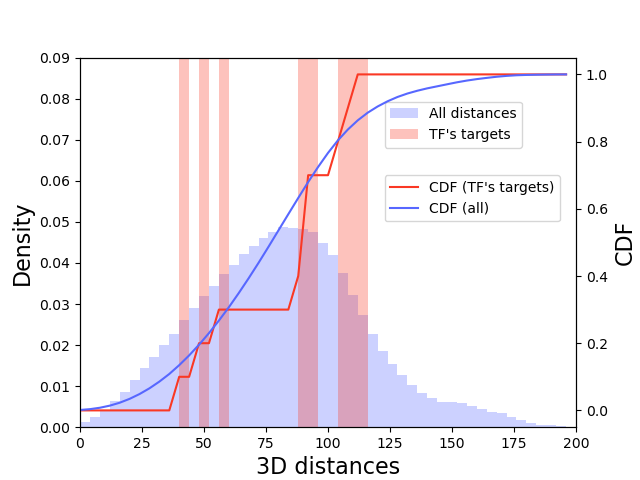

Supplement: Supplementary file 4 — Additional file 4. ZIP file with graphical representations associated to each transcriptional module: (link from Zenodo repository: https://zenodo.org/record/5841177/files/supplementary-data-file-S4.zip?download=1). [file 13104_2022_5940_MOESM4_ESM.zip › 3D_distances_distribution/OAF3_5_targets.csv.png]

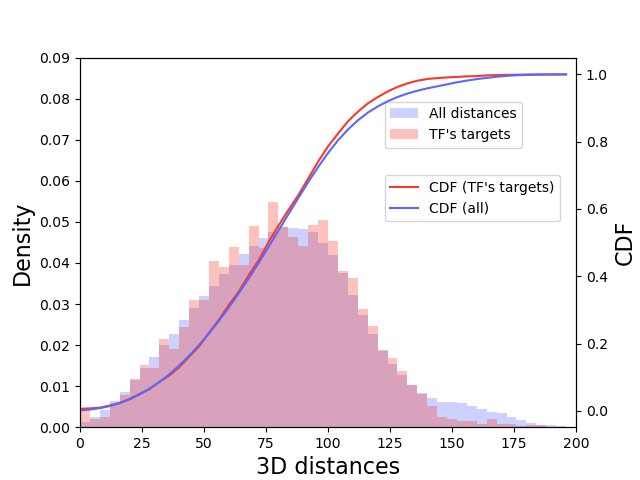

Supplement: Supplementary file 4 — Additional file 4. ZIP file with graphical representations associated to each transcriptional module: (link from Zenodo repository: https://zenodo.org/record/5841177/files/supplementary-data-file-S4.zip?download=1). [file 13104_2022_5940_MOESM4_ESM.zip › 3D_distances_distribution/RIM101_150_targets.csv.png]

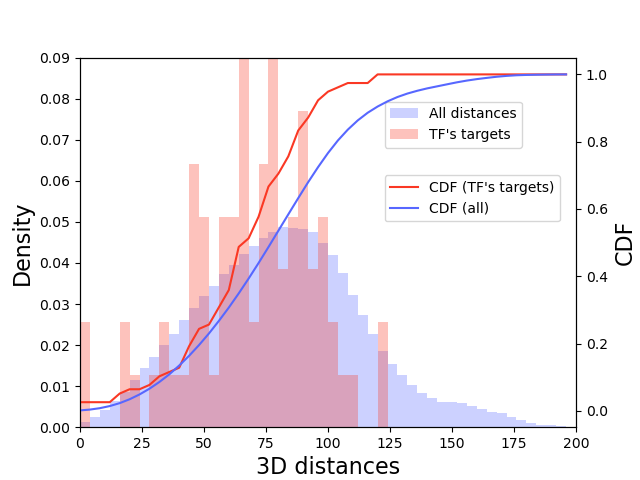

Supplement: Supplementary file 4 — Additional file 4. ZIP file with graphical representations associated to each transcriptional module: (link from Zenodo repository: https://zenodo.org/record/5841177/files/supplementary-data-file-S4.zip?download=1). [file 13104_2022_5940_MOESM4_ESM.zip › 3D_distances_distribution/WAR1_13_targets.csv.png]

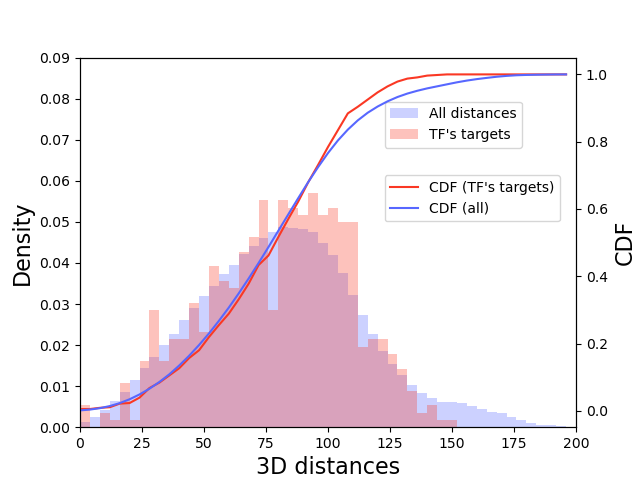

Supplement: Supplementary file 4 — Additional file 4. ZIP file with graphical representations associated to each transcriptional module: (link from Zenodo repository: https://zenodo.org/record/5841177/files/supplementary-data-file-S4.zip?download=1). [file 13104_2022_5940_MOESM4_ESM.zip › 3D_distances_distribution/ERT1_34_targets.csv.png]

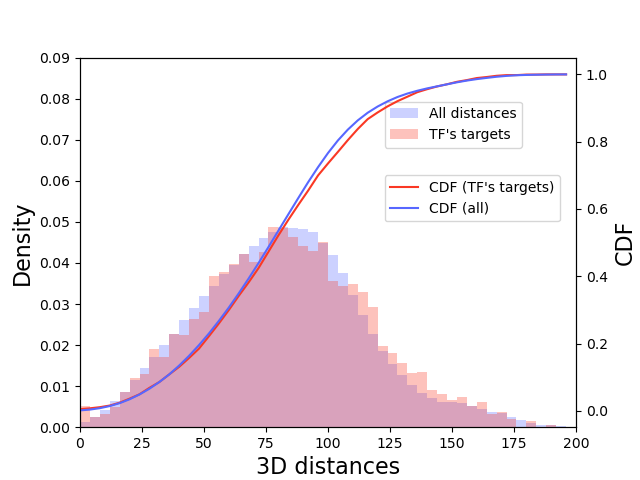

Supplement: Supplementary file 4 — Additional file 4. ZIP file with graphical representations associated to each transcriptional module: (link from Zenodo repository: https://zenodo.org/record/5841177/files/supplementary-data-file-S4.zip?download=1). [file 13104_2022_5940_MOESM4_ESM.zip › 3D_distances_distribution/HAP2_171_targets.csv.png]

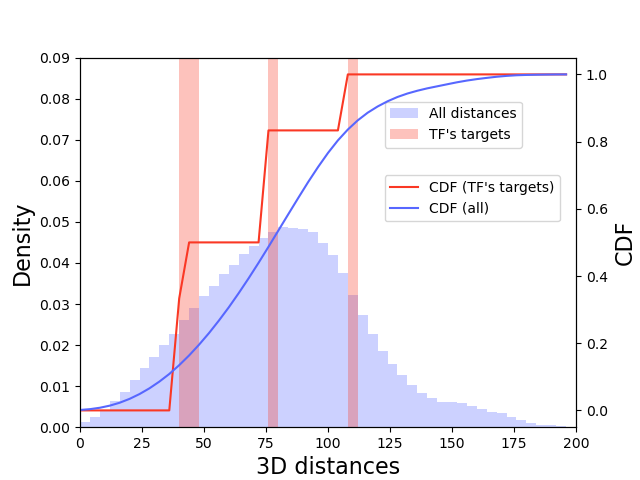

Supplement: Supplementary file 4 — Additional file 4. ZIP file with graphical representations associated to each transcriptional module: (link from Zenodo repository: https://zenodo.org/record/5841177/files/supplementary-data-file-S4.zip?download=1). [file 13104_2022_5940_MOESM4_ESM.zip › 3D_distances_distribution/HMO1_4_targets.csv.png]

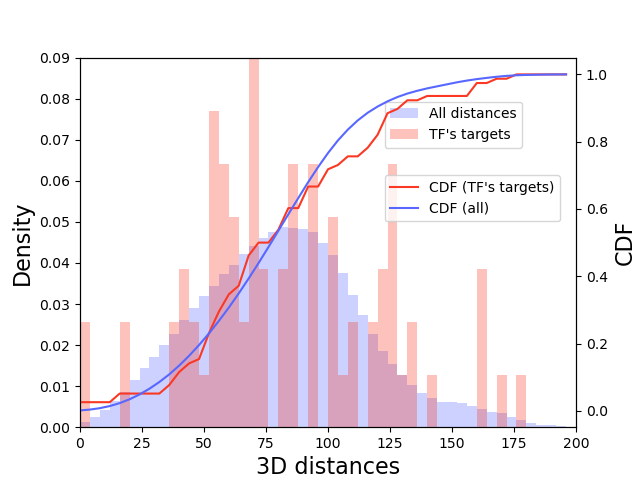

Supplement: Supplementary file 4 — Additional file 4. ZIP file with graphical representations associated to each transcriptional module: (link from Zenodo repository: https://zenodo.org/record/5841177/files/supplementary-data-file-S4.zip?download=1). [file 13104_2022_5940_MOESM4_ESM.zip › 3D_distances_distribution/TOG1_13_targets.csv.png]

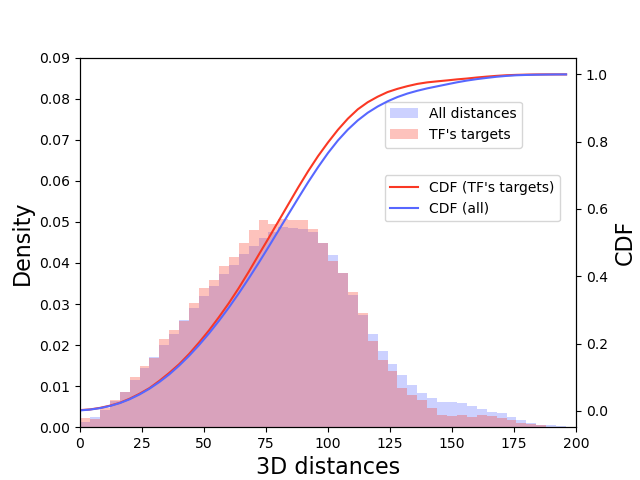

Supplement: Supplementary file 4 — Additional file 4. ZIP file with graphical representations associated to each transcriptional module: (link from Zenodo repository: https://zenodo.org/record/5841177/files/supplementary-data-file-S4.zip?download=1). [file 13104_2022_5940_MOESM4_ESM.zip › 3D_distances_distribution/OAF1_493_targets.csv.png]

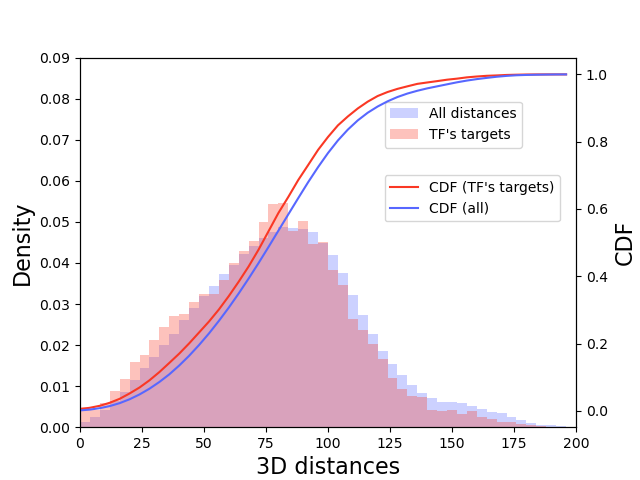

Supplement: Supplementary file 4 — Additional file 4. ZIP file with graphical representations associated to each transcriptional module: (link from Zenodo repository: https://zenodo.org/record/5841177/files/supplementary-data-file-S4.zip?download=1). [file 13104_2022_5940_MOESM4_ESM.zip › 3D_distances_distribution/HSF1_245_targets.csv.png]

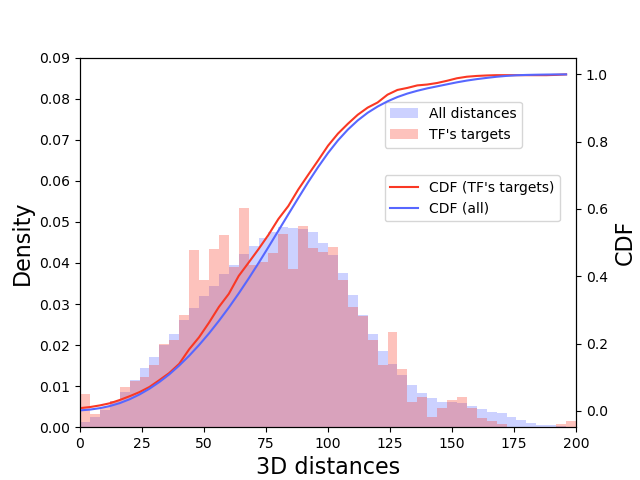

Supplement: Supplementary file 4 — Additional file 4. ZIP file with graphical representations associated to each transcriptional module: (link from Zenodo repository: https://zenodo.org/record/5841177/files/supplementary-data-file-S4.zip?download=1). [file 13104_2022_5940_MOESM4_ESM.zip › 3D_distances_distribution/ZAP1_84_targets.csv.png]

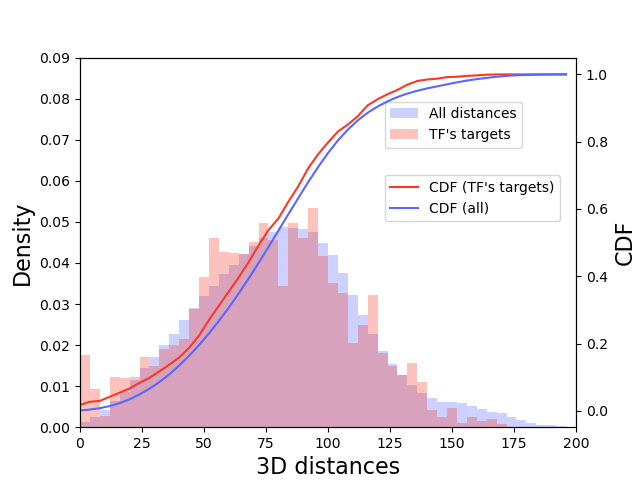

Supplement: Supplementary file 4 — Additional file 4. ZIP file with graphical representations associated to each transcriptional module: (link from Zenodo repository: https://zenodo.org/record/5841177/files/supplementary-data-file-S4.zip?download=1). [file 13104_2022_5940_MOESM4_ESM.zip › 3D_distances_distribution/ARO80_95_targets.csv.png]

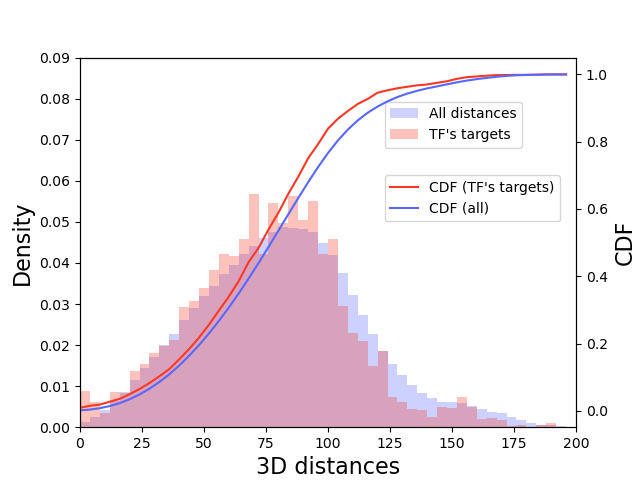

Supplement: Supplementary file 4 — Additional file 4. ZIP file with graphical representations associated to each transcriptional module: (link from Zenodo repository: https://zenodo.org/record/5841177/files/supplementary-data-file-S4.zip?download=1). [file 13104_2022_5940_MOESM4_ESM.zip › 3D_distances_distribution/ARG81_93_targets.csv.png]

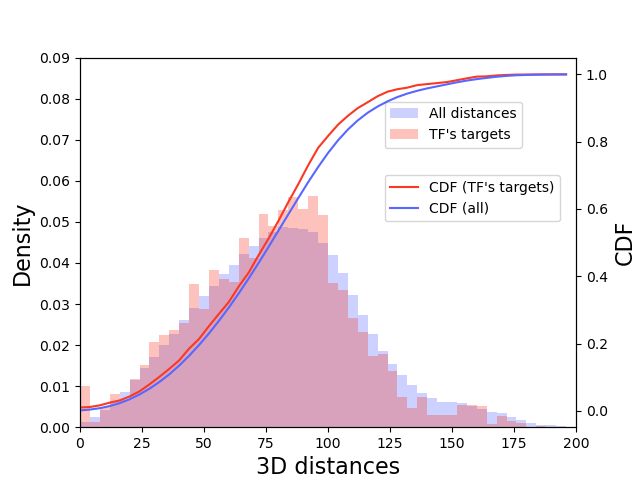

Supplement: Supplementary file 4 — Additional file 4. ZIP file with graphical representations associated to each transcriptional module: (link from Zenodo repository: https://zenodo.org/record/5841177/files/supplementary-data-file-S4.zip?download=1). [file 13104_2022_5940_MOESM4_ESM.zip › 3D_distances_distribution/ACE2_154_targets.csv.png]

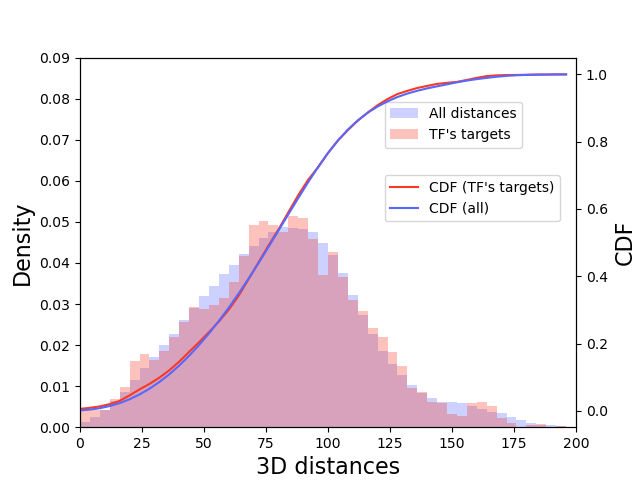

Supplement: Supplementary file 4 — Additional file 4. ZIP file with graphical representations associated to each transcriptional module: (link from Zenodo repository: https://zenodo.org/record/5841177/files/supplementary-data-file-S4.zip?download=1). [file 13104_2022_5940_MOESM4_ESM.zip › 3D_distances_distribution/GCR2_142_targets.csv.png]

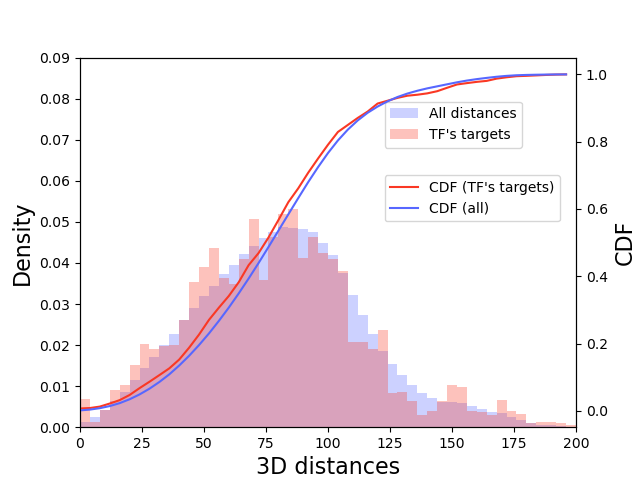

Supplement: Supplementary file 4 — Additional file 4. ZIP file with graphical representations associated to each transcriptional module: (link from Zenodo repository: https://zenodo.org/record/5841177/files/supplementary-data-file-S4.zip?download=1). [file 13104_2022_5940_MOESM4_ESM.zip › 3D_distances_distribution/MET31_87_targets.csv.png]

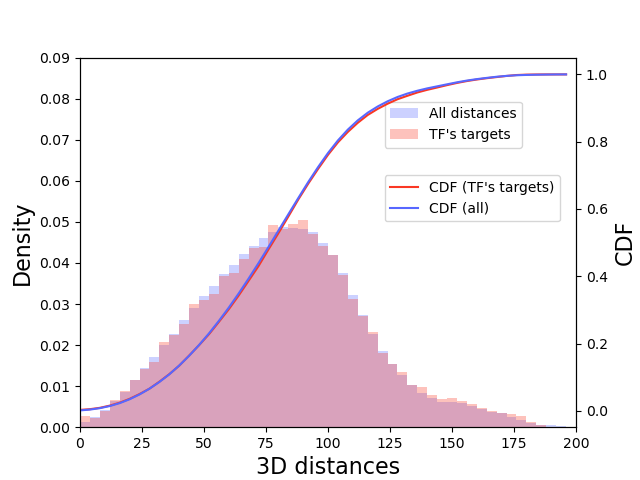

Supplement: Supplementary file 4 — Additional file 4. ZIP file with graphical representations associated to each transcriptional module: (link from Zenodo repository: https://zenodo.org/record/5841177/files/supplementary-data-file-S4.zip?download=1). [file 13104_2022_5940_MOESM4_ESM.zip › 3D_distances_distribution/FHL1_849_targets.csv.png]

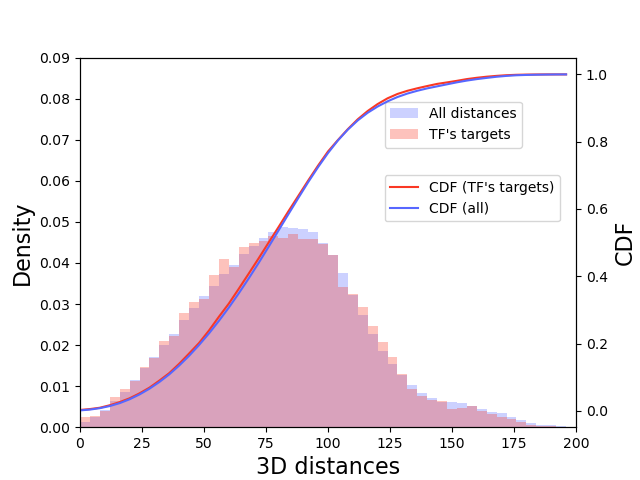

Supplement: Supplementary file 4 — Additional file 4. ZIP file with graphical representations associated to each transcriptional module: (link from Zenodo repository: https://zenodo.org/record/5841177/files/supplementary-data-file-S4.zip?download=1). [file 13104_2022_5940_MOESM4_ESM.zip › 3D_distances_distribution/ABF1_357_targets.csv.png]

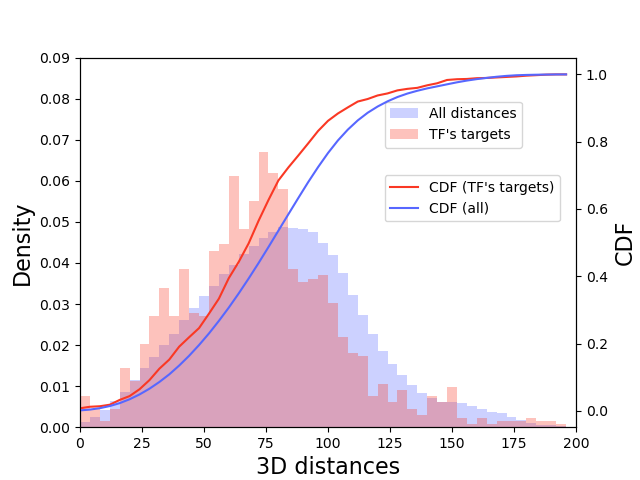

Supplement: Supplementary file 4 — Additional file 4. ZIP file with graphical representations associated to each transcriptional module: (link from Zenodo repository: https://zenodo.org/record/5841177/files/supplementary-data-file-S4.zip?download=1). [file 13104_2022_5940_MOESM4_ESM.zip › 3D_distances_distribution/AZF1_52_targets.csv.png]

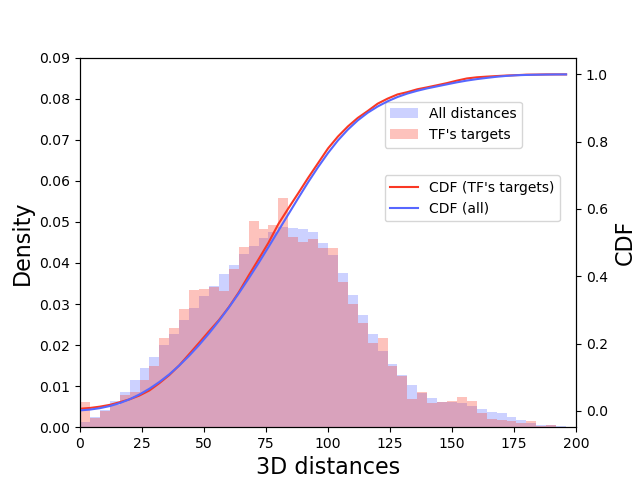

Supplement: Supplementary file 4 — Additional file 4. ZIP file with graphical representations associated to each transcriptional module: (link from Zenodo repository: https://zenodo.org/record/5841177/files/supplementary-data-file-S4.zip?download=1). [file 13104_2022_5940_MOESM4_ESM.zip › 3D_distances_distribution/SWI5_217_targets.csv.png]

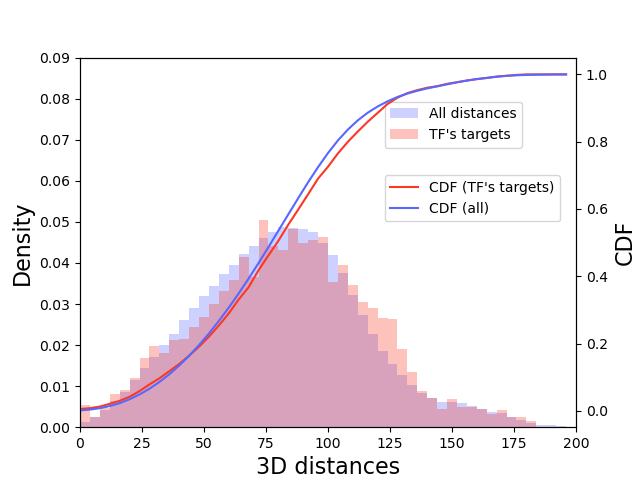

Supplement: Supplementary file 4 — Additional file 4. ZIP file with graphical representations associated to each transcriptional module: (link from Zenodo repository: https://zenodo.org/record/5841177/files/supplementary-data-file-S4.zip?download=1). [file 13104_2022_5940_MOESM4_ESM.zip › 3D_distances_distribution/GAT1_139_targets.csv.png]

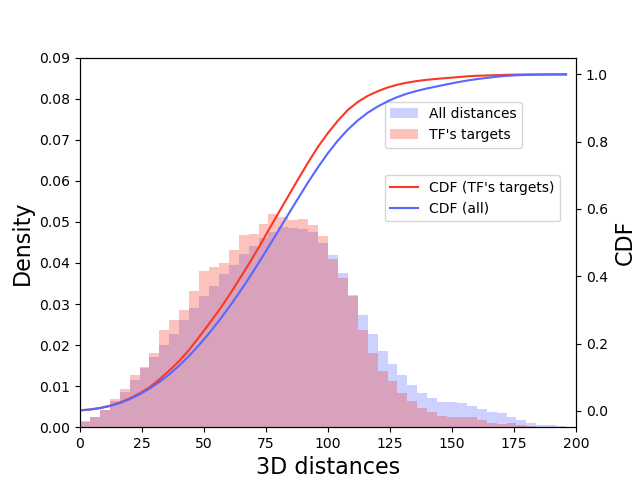

Supplement: Supplementary file 4 — Additional file 4. ZIP file with graphical representations associated to each transcriptional module: (link from Zenodo repository: https://zenodo.org/record/5841177/files/supplementary-data-file-S4.zip?download=1). [file 13104_2022_5940_MOESM4_ESM.zip › 3D_distances_distribution/XBP1_407_targets.csv.png]

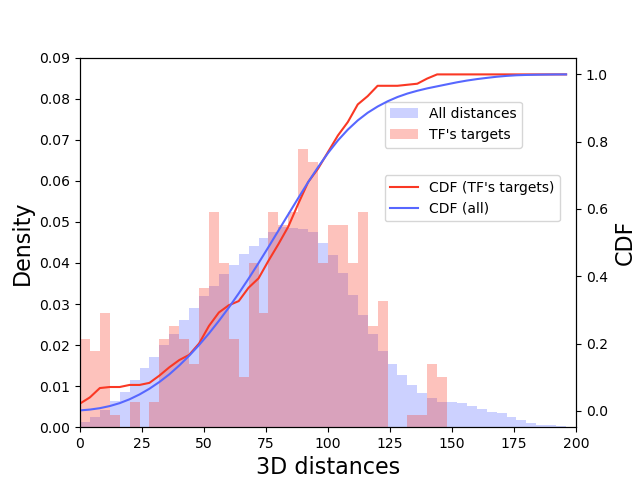

Supplement: Supplementary file 4 — Additional file 4. ZIP file with graphical representations associated to each transcriptional module: (link from Zenodo repository: https://zenodo.org/record/5841177/files/supplementary-data-file-S4.zip?download=1). [file 13104_2022_5940_MOESM4_ESM.zip › 3D_distances_distribution/PPR1_26_targets.csv.png]

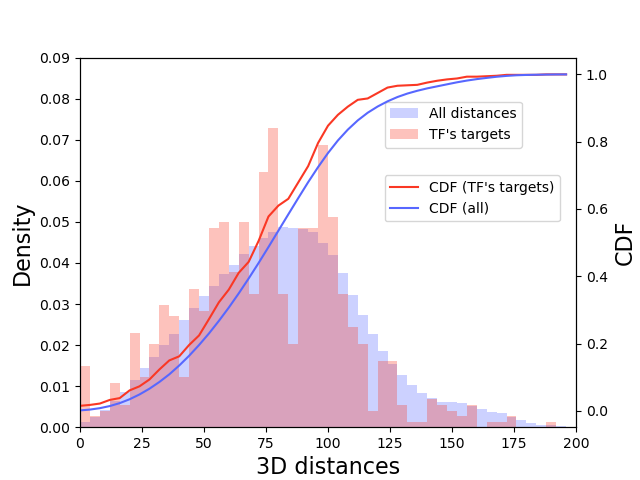

Supplement: Supplementary file 4 — Additional file 4. ZIP file with graphical representations associated to each transcriptional module: (link from Zenodo repository: https://zenodo.org/record/5841177/files/supplementary-data-file-S4.zip?download=1). [file 13104_2022_5940_MOESM4_ESM.zip › 3D_distances_distribution/YAP3_39_targets.csv.png]

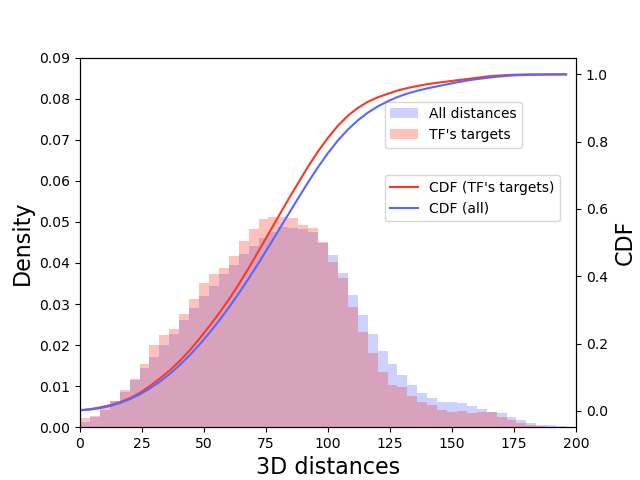

Supplement: Supplementary file 4 — Additional file 4. ZIP file with graphical representations associated to each transcriptional module: (link from Zenodo repository: https://zenodo.org/record/5841177/files/supplementary-data-file-S4.zip?download=1). [file 13104_2022_5940_MOESM4_ESM.zip › 3D_distances_distribution/REB1_543_targets.csv.png]

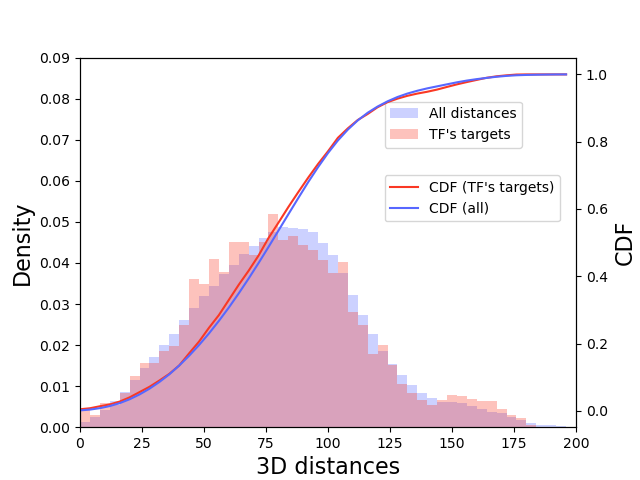

Supplement: Supplementary file 4 — Additional file 4. ZIP file with graphical representations associated to each transcriptional module: (link from Zenodo repository: https://zenodo.org/record/5841177/files/supplementary-data-file-S4.zip?download=1). [file 13104_2022_5940_MOESM4_ESM.zip › 3D_distances_distribution/UME6_178_targets.csv.png]

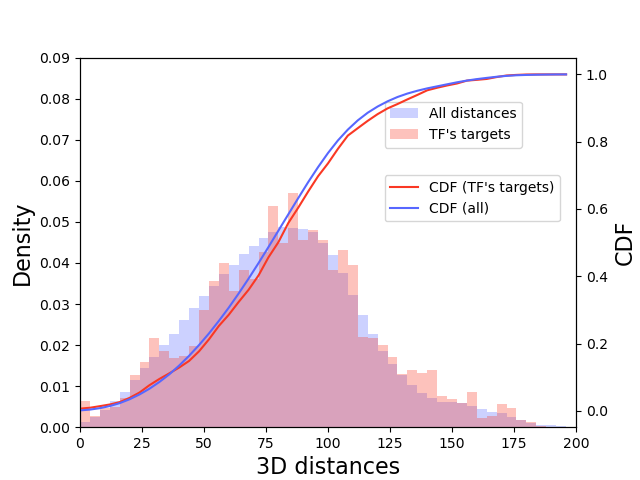

Supplement: Supplementary file 4 — Additional file 4. ZIP file with graphical representations associated to each transcriptional module: (link from Zenodo repository: https://zenodo.org/record/5841177/files/supplementary-data-file-S4.zip?download=1). [file 13104_2022_5940_MOESM4_ESM.zip › 3D_distances_distribution/SIP4_83_targets.csv.png]

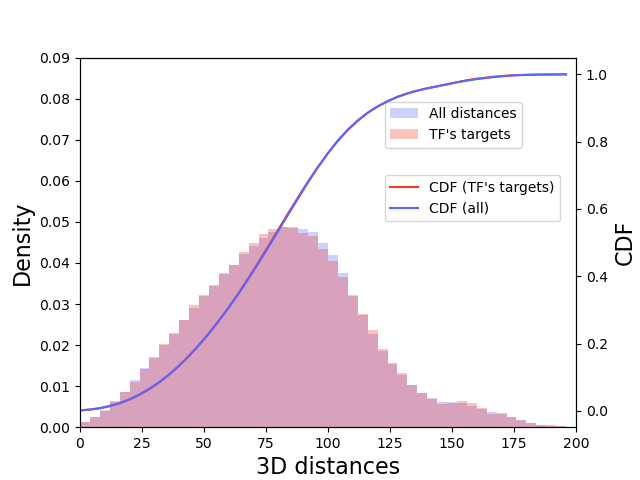

Supplement: Supplementary file 4 — Additional file 4. ZIP file with graphical representations associated to each transcriptional module: (link from Zenodo repository: https://zenodo.org/record/5841177/files/supplementary-data-file-S4.zip?download=1). [file 13104_2022_5940_MOESM4_ESM.zip › 3D_distances_distribution/SPT23_1758_targets.csv.png]

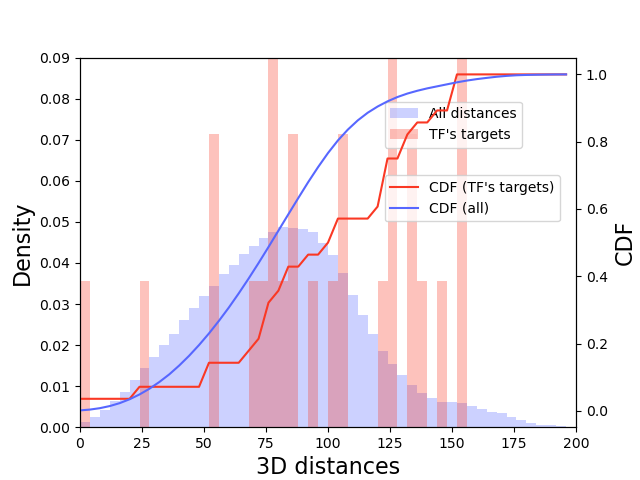

Supplement: Supplementary file 4 — Additional file 4. ZIP file with graphical representations associated to each transcriptional module: (link from Zenodo repository: https://zenodo.org/record/5841177/files/supplementary-data-file-S4.zip?download=1). [file 13104_2022_5940_MOESM4_ESM.zip › 3D_distances_distribution/TDA9_8_targets.csv.png]

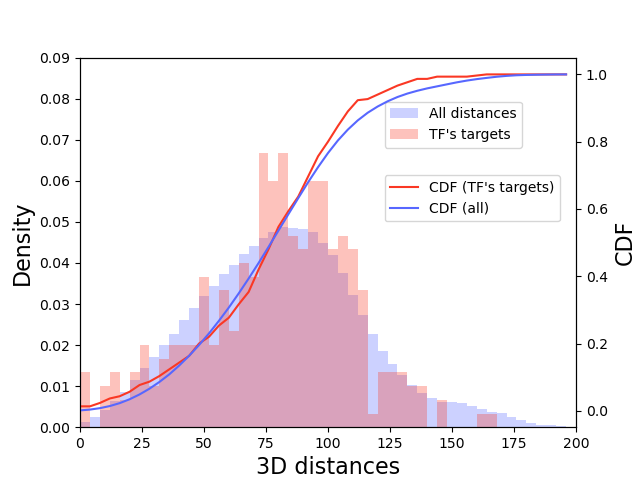

Supplement: Supplementary file 4 — Additional file 4. ZIP file with graphical representations associated to each transcriptional module: (link from Zenodo repository: https://zenodo.org/record/5841177/files/supplementary-data-file-S4.zip?download=1). [file 13104_2022_5940_MOESM4_ESM.zip › 3D_distances_distribution/MIG2_25_targets.csv.png]

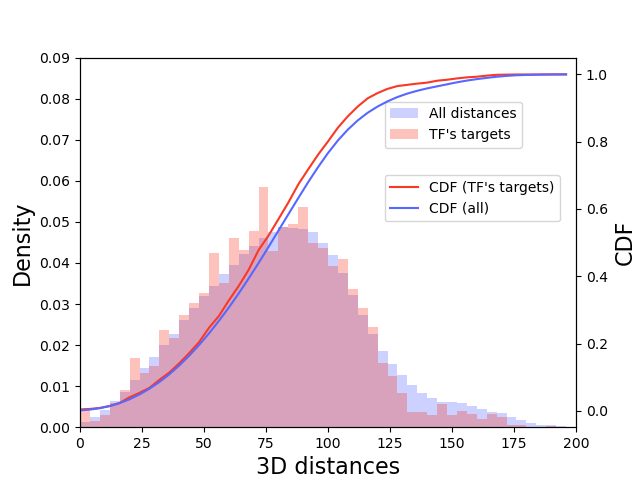

Supplement: Supplementary file 4 — Additional file 4. ZIP file with graphical representations associated to each transcriptional module: (link from Zenodo repository: https://zenodo.org/record/5841177/files/supplementary-data-file-S4.zip?download=1). [file 13104_2022_5940_MOESM4_ESM.zip › 3D_distances_distribution/CUP9_91_targets.csv.png]

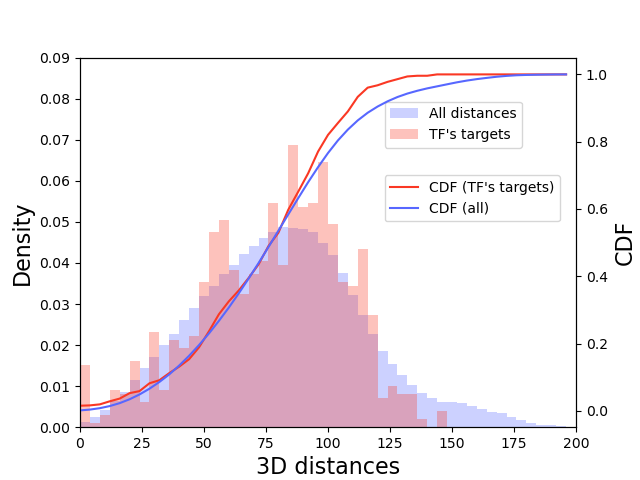

Supplement: Supplementary file 4 — Additional file 4. ZIP file with graphical representations associated to each transcriptional module: (link from Zenodo repository: https://zenodo.org/record/5841177/files/supplementary-data-file-S4.zip?download=1). [file 13104_2022_5940_MOESM4_ESM.zip › 3D_distances_distribution/RDS1_45_targets.csv.png]

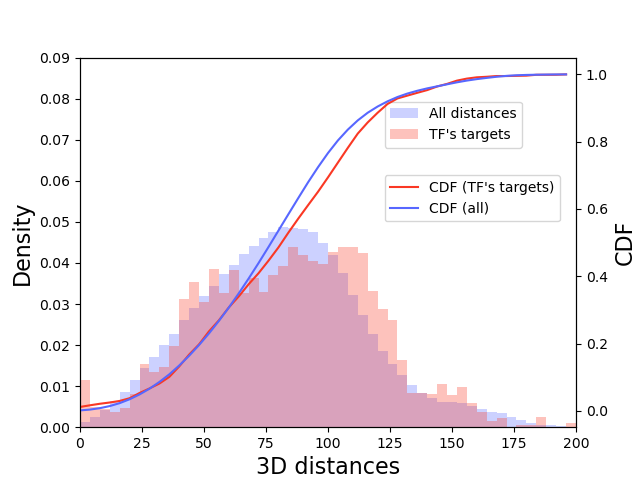

Supplement: Supplementary file 4 — Additional file 4. ZIP file with graphical representations associated to each transcriptional module: (link from Zenodo repository: https://zenodo.org/record/5841177/files/supplementary-data-file-S4.zip?download=1). [file 13104_2022_5940_MOESM4_ESM.zip › 3D_distances_distribution/GAL4_132_targets.csv.png]

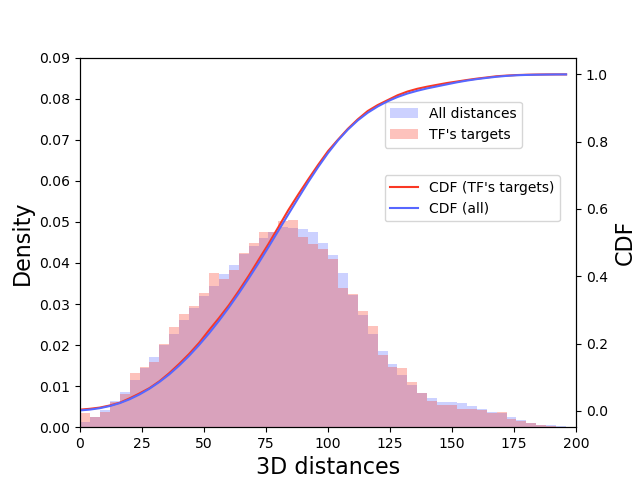

Supplement: Supplementary file 4 — Additional file 4. ZIP file with graphical representations associated to each transcriptional module: (link from Zenodo repository: https://zenodo.org/record/5841177/files/supplementary-data-file-S4.zip?download=1). [file 13104_2022_5940_MOESM4_ESM.zip › 3D_distances_distribution/MCM1_397_targets.csv.png]

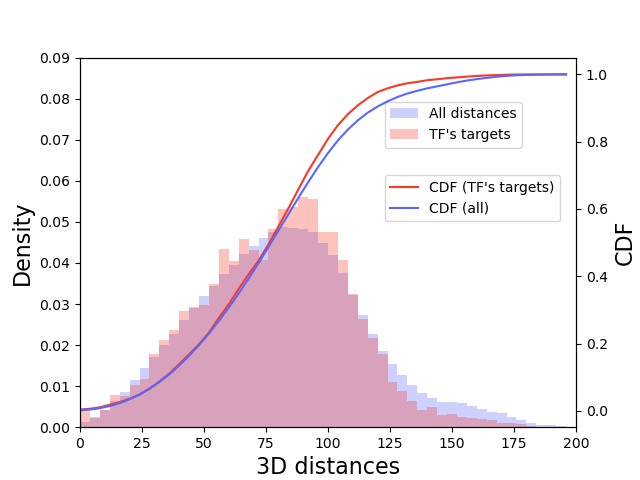

Supplement: Supplementary file 4 — Additional file 4. ZIP file with graphical representations associated to each transcriptional module: (link from Zenodo repository: https://zenodo.org/record/5841177/files/supplementary-data-file-S4.zip?download=1). [file 13104_2022_5940_MOESM4_ESM.zip › 3D_distances_distribution/CRZ1_242_targets.csv.png]

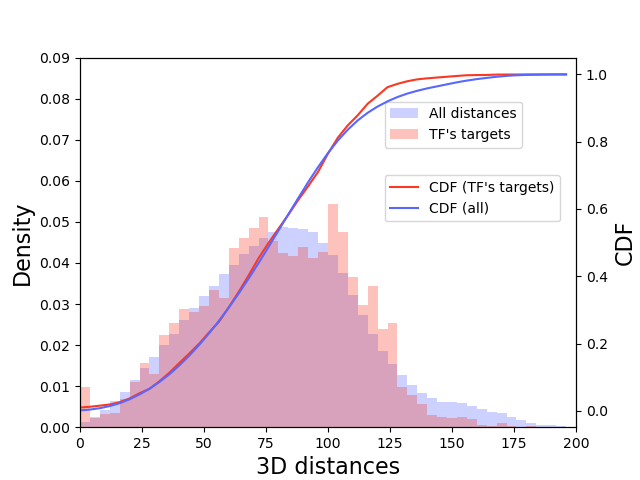

Supplement: Supplementary file 4 — Additional file 4. ZIP file with graphical representations associated to each transcriptional module: (link from Zenodo repository: https://zenodo.org/record/5841177/files/supplementary-data-file-S4.zip?download=1). [file 13104_2022_5940_MOESM4_ESM.zip › 3D_distances_distribution/GAT3_148_targets.csv.png]

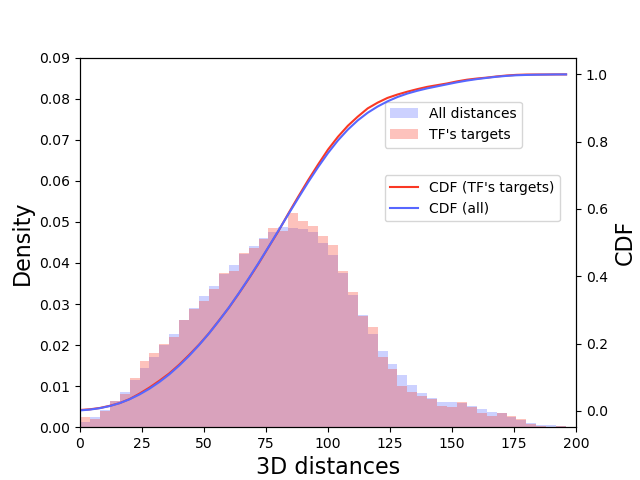

Supplement: Supplementary file 4 — Additional file 4. ZIP file with graphical representations associated to each transcriptional module: (link from Zenodo repository: https://zenodo.org/record/5841177/files/supplementary-data-file-S4.zip?download=1). [file 13104_2022_5940_MOESM4_ESM.zip › 3D_distances_distribution/ADR1_343_targets.csv.png]

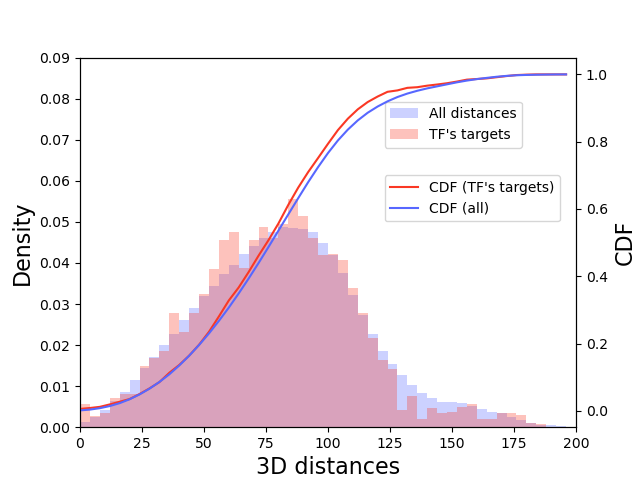

Supplement: Supplementary file 4 — Additional file 4. ZIP file with graphical representations associated to each transcriptional module: (link from Zenodo repository: https://zenodo.org/record/5841177/files/supplementary-data-file-S4.zip?download=1). [file 13104_2022_5940_MOESM4_ESM.zip › 3D_distances_distribution/HOT1_73_targets.csv.png]

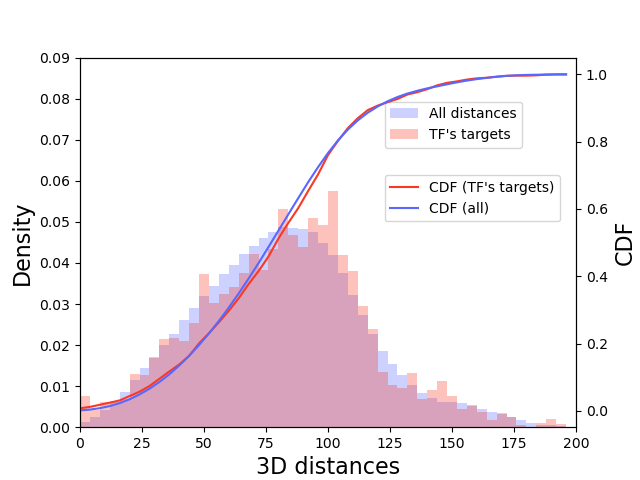

Supplement: Supplementary file 4 — Additional file 4. ZIP file with graphical representations associated to each transcriptional module: (link from Zenodo repository: https://zenodo.org/record/5841177/files/supplementary-data-file-S4.zip?download=1). [file 13104_2022_5940_MOESM4_ESM.zip › 3D_distances_distribution/MAC1_105_targets.csv.png]

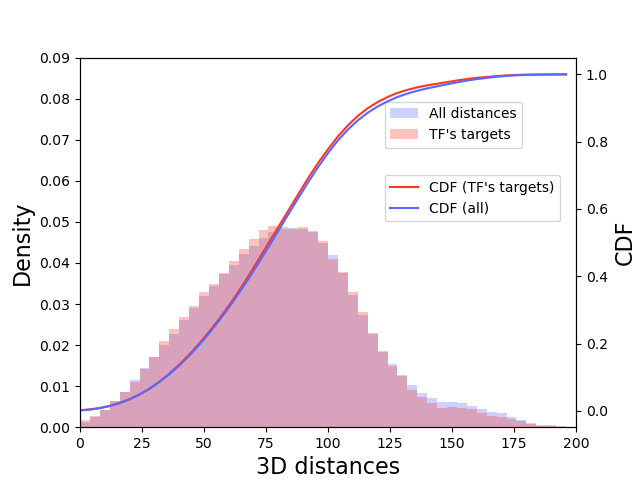

Supplement: Supplementary file 4 — Additional file 4. ZIP file with graphical representations associated to each transcriptional module: (link from Zenodo repository: https://zenodo.org/record/5841177/files/supplementary-data-file-S4.zip?download=1). [file 13104_2022_5940_MOESM4_ESM.zip › 3D_distances_distribution/CIN5_1082_targets.csv.png]

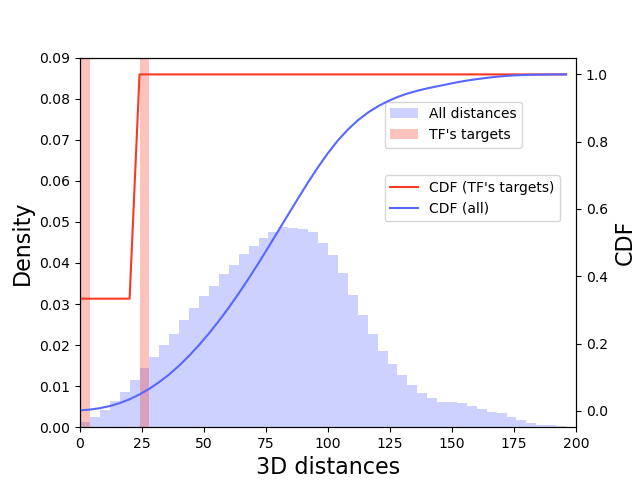

Supplement: Supplementary file 4 — Additional file 4. ZIP file with graphical representations associated to each transcriptional module: (link from Zenodo repository: https://zenodo.org/record/5841177/files/supplementary-data-file-S4.zip?download=1). [file 13104_2022_5940_MOESM4_ESM.zip › 3D_distances_distribution/EDS1_3_targets.csv.png]

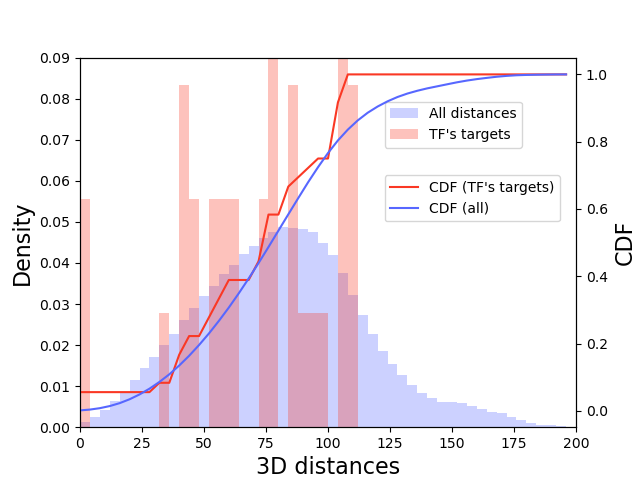

Supplement: Supplementary file 4 — Additional file 4. ZIP file with graphical representations associated to each transcriptional module: (link from Zenodo repository: https://zenodo.org/record/5841177/files/supplementary-data-file-S4.zip?download=1). [file 13104_2022_5940_MOESM4_ESM.zip › 3D_distances_distribution/MIG3_9_targets.csv.png]

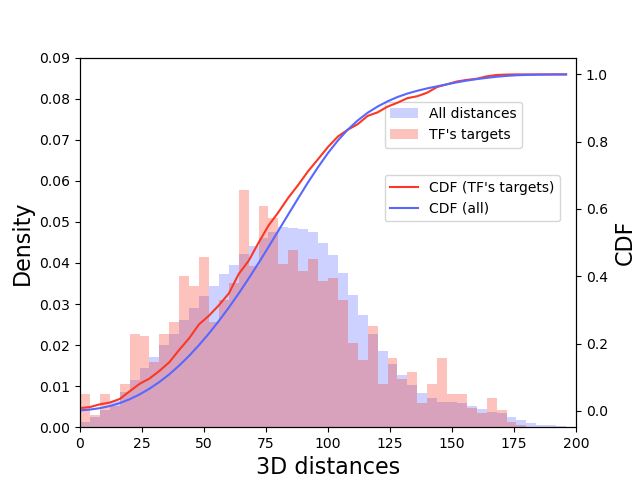

Supplement: Supplementary file 4 — Additional file 4. ZIP file with graphical representations associated to each transcriptional module: (link from Zenodo repository: https://zenodo.org/record/5841177/files/supplementary-data-file-S4.zip?download=1). [file 13104_2022_5940_MOESM4_ESM.zip › 3D_distances_distribution/HMS1_59_targets.csv.png]

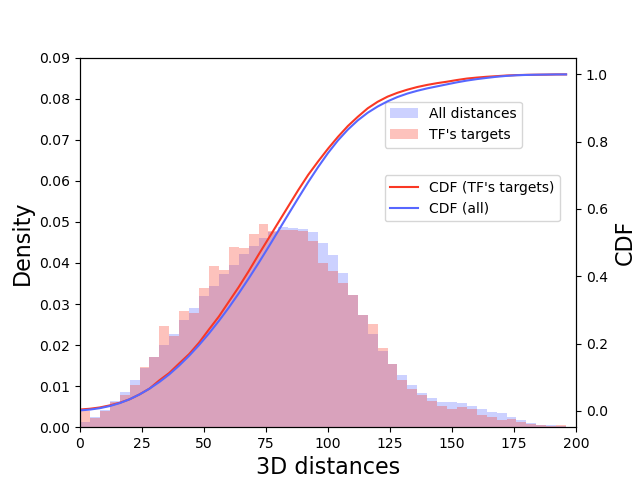

Supplement: Supplementary file 4 — Additional file 4. ZIP file with graphical representations associated to each transcriptional module: (link from Zenodo repository: https://zenodo.org/record/5841177/files/supplementary-data-file-S4.zip?download=1). [file 13104_2022_5940_MOESM4_ESM.zip › 3D_distances_distribution/NRG1_269_targets.csv.png]

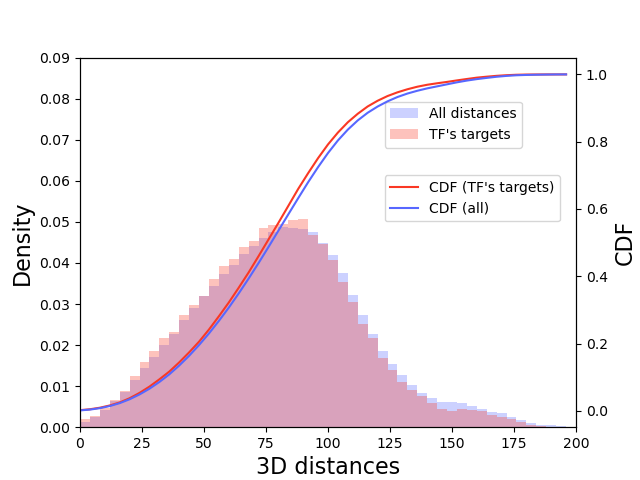

Supplement: Supplementary file 4 — Additional file 4. ZIP file with graphical representations associated to each transcriptional module: (link from Zenodo repository: https://zenodo.org/record/5841177/files/supplementary-data-file-S4.zip?download=1). [file 13104_2022_5940_MOESM4_ESM.zip › 3D_distances_distribution/INO2_824_targets.csv.png]

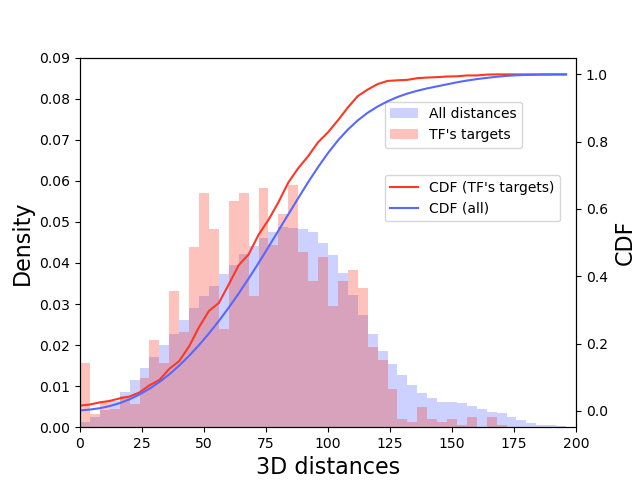

Supplement: Supplementary file 4 — Additional file 4. ZIP file with graphical representations associated to each transcriptional module: (link from Zenodo repository: https://zenodo.org/record/5841177/files/supplementary-data-file-S4.zip?download=1). [file 13104_2022_5940_MOESM4_ESM.zip › 3D_distances_distribution/DAL80_57_targets.csv.png]

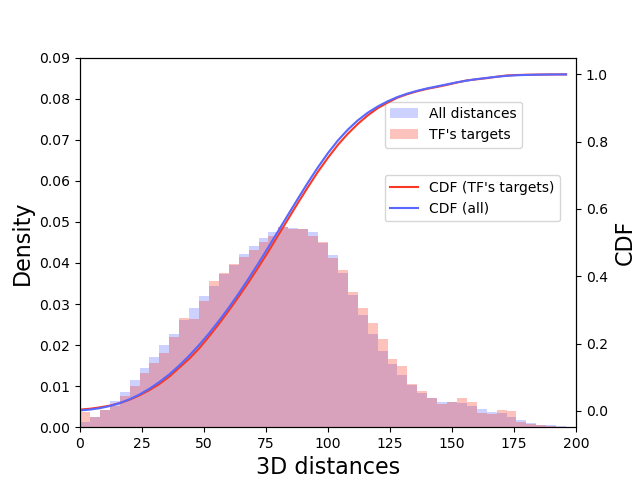

Supplement: Supplementary file 4 — Additional file 4. ZIP file with graphical representations associated to each transcriptional module: (link from Zenodo repository: https://zenodo.org/record/5841177/files/supplementary-data-file-S4.zip?download=1). [file 13104_2022_5940_MOESM4_ESM.zip › 3D_distances_distribution/SWI4_489_targets.csv.png]

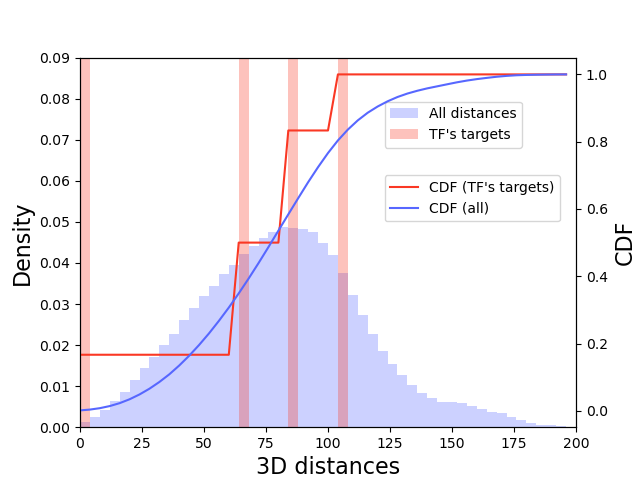

Supplement: Supplementary file 4 — Additional file 4. ZIP file with graphical representations associated to each transcriptional module: (link from Zenodo repository: https://zenodo.org/record/5841177/files/supplementary-data-file-S4.zip?download=1). [file 13104_2022_5940_MOESM4_ESM.zip › 3D_distances_distribution/CUP2_4_targets.csv.png]

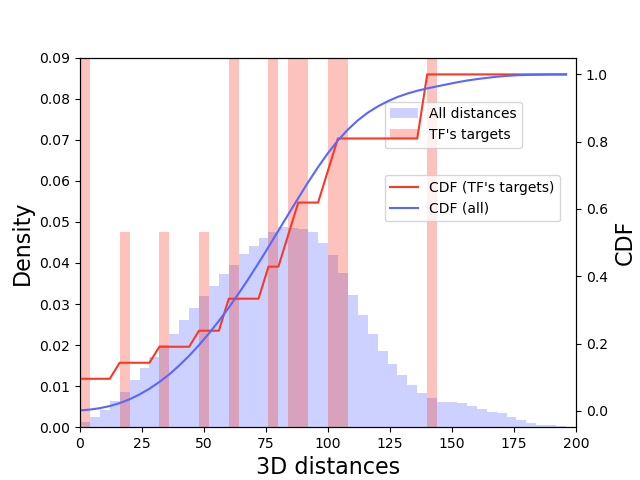

Supplement: Supplementary file 4 — Additional file 4. ZIP file with graphical representations associated to each transcriptional module: (link from Zenodo repository: https://zenodo.org/record/5841177/files/supplementary-data-file-S4.zip?download=1). [file 13104_2022_5940_MOESM4_ESM.zip › 3D_distances_distribution/URC2_7_targets.csv.png]

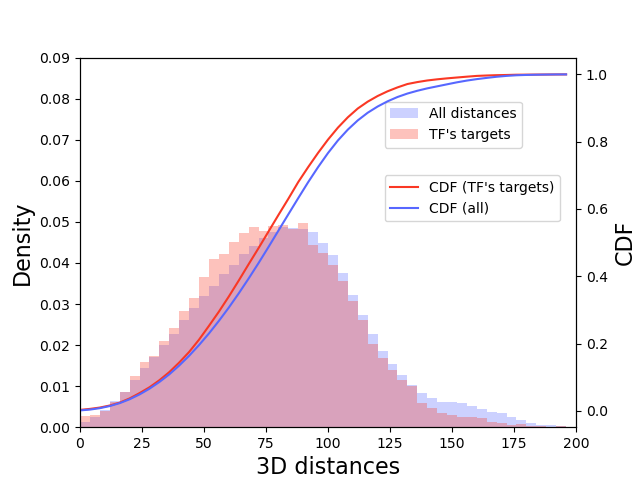

Supplement: Supplementary file 4 — Additional file 4. ZIP file with graphical representations associated to each transcriptional module: (link from Zenodo repository: https://zenodo.org/record/5841177/files/supplementary-data-file-S4.zip?download=1). [file 13104_2022_5940_MOESM4_ESM.zip › 3D_distances_distribution/MGA1_348_targets.csv.png]

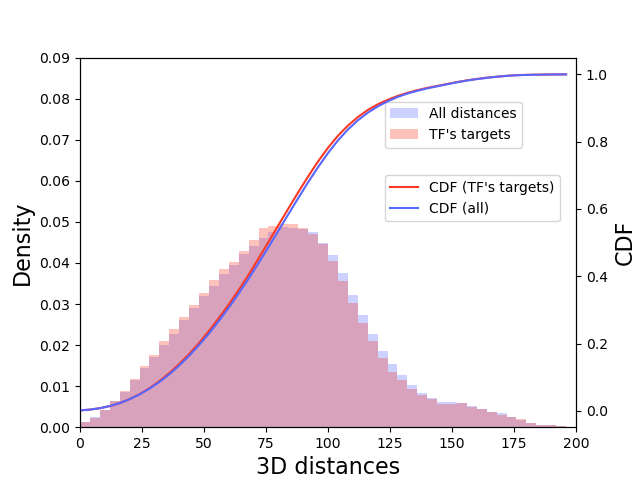

Supplement: Supplementary file 4 — Additional file 4. ZIP file with graphical representations associated to each transcriptional module: (link from Zenodo repository: https://zenodo.org/record/5841177/files/supplementary-data-file-S4.zip?download=1). [file 13104_2022_5940_MOESM4_ESM.zip › 3D_distances_distribution/STE12_2082_targets.csv.png]

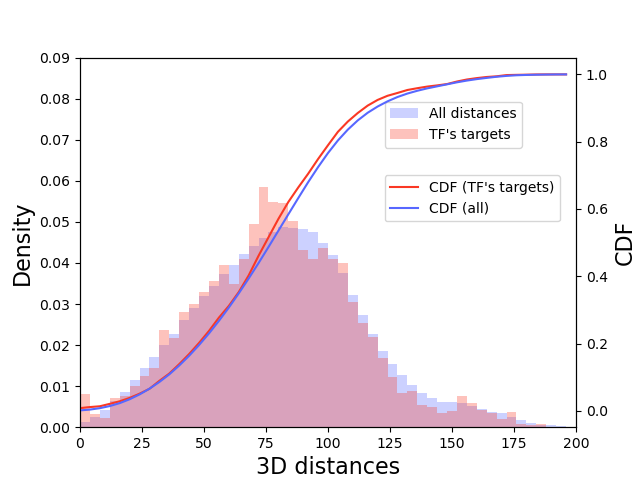

Supplement: Supplementary file 4 — Additional file 4. ZIP file with graphical representations associated to each transcriptional module: (link from Zenodo repository: https://zenodo.org/record/5841177/files/supplementary-data-file-S4.zip?download=1). [file 13104_2022_5940_MOESM4_ESM.zip › 3D_distances_distribution/NDD1_110_targets.csv.png]

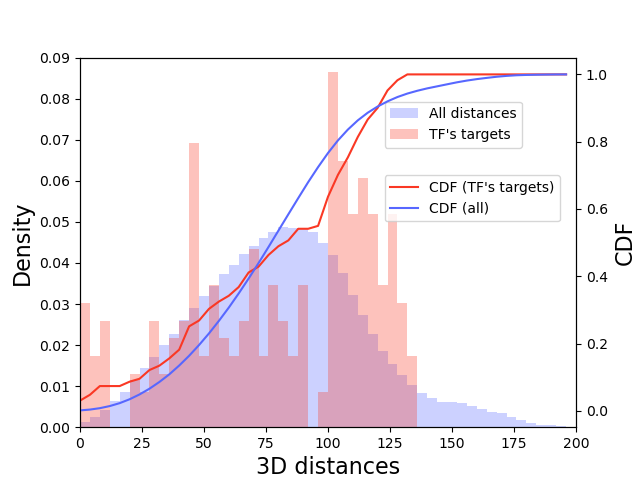

Supplement: Supplementary file 4 — Additional file 4. ZIP file with graphical representations associated to each transcriptional module: (link from Zenodo repository: https://zenodo.org/record/5841177/files/supplementary-data-file-S4.zip?download=1). [file 13104_2022_5940_MOESM4_ESM.zip › 3D_distances_distribution/PDR3_22_targets.csv.png]

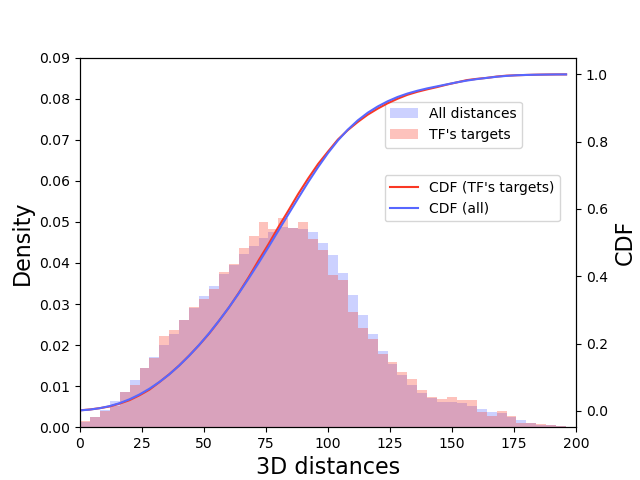

Supplement: Supplementary file 4 — Additional file 4. ZIP file with graphical representations associated to each transcriptional module: (link from Zenodo repository: https://zenodo.org/record/5841177/files/supplementary-data-file-S4.zip?download=1). [file 13104_2022_5940_MOESM4_ESM.zip › 3D_distances_distribution/TUP1_251_targets.csv.png]

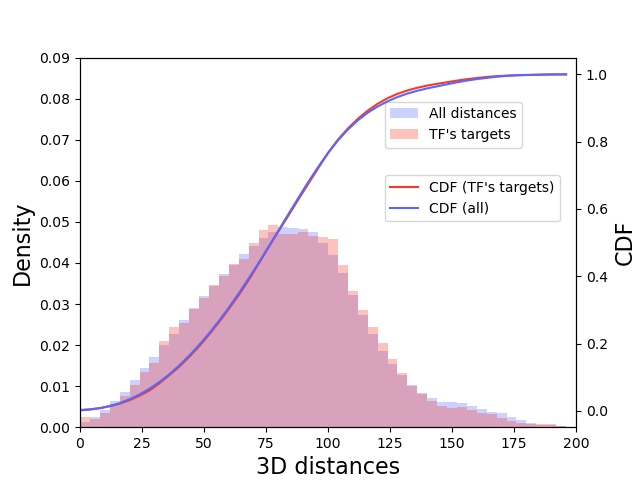

Supplement: Supplementary file 4 — Additional file 4. ZIP file with graphical representations associated to each transcriptional module: (link from Zenodo repository: https://zenodo.org/record/5841177/files/supplementary-data-file-S4.zip?download=1). [file 13104_2022_5940_MOESM4_ESM.zip › 3D_distances_distribution/YAP5_527_targets.csv.png]

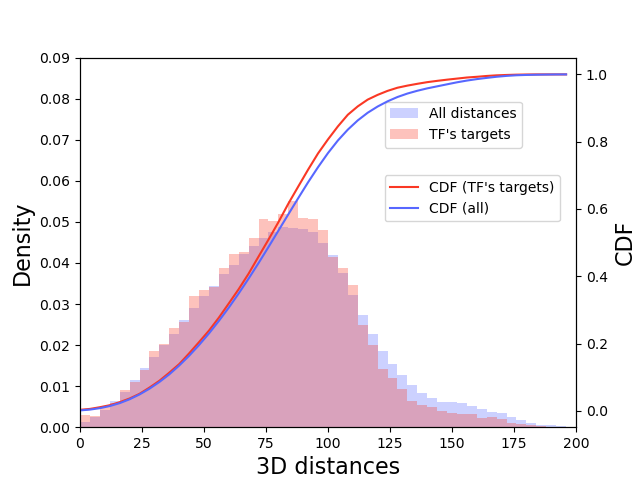

Supplement: Supplementary file 4 — Additional file 4. ZIP file with graphical representations associated to each transcriptional module: (link from Zenodo repository: https://zenodo.org/record/5841177/files/supplementary-data-file-S4.zip?download=1). [file 13104_2022_5940_MOESM4_ESM.zip › 3D_distances_distribution/AFT1_396_targets.csv.png]

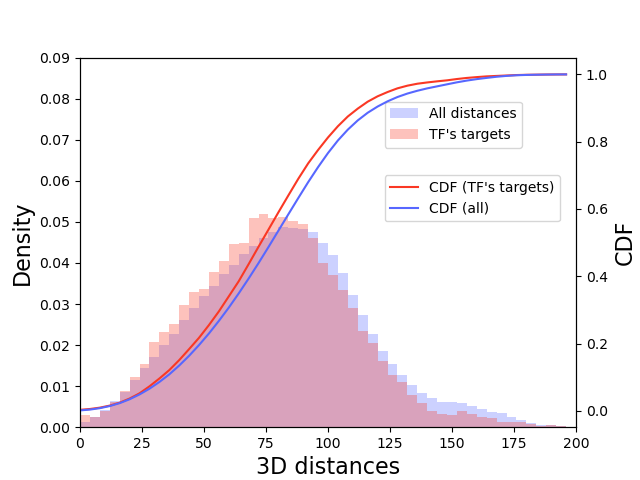

Supplement: Supplementary file 4 — Additional file 4. ZIP file with graphical representations associated to each transcriptional module: (link from Zenodo repository: https://zenodo.org/record/5841177/files/supplementary-data-file-S4.zip?download=1). [file 13104_2022_5940_MOESM4_ESM.zip › 3D_distances_distribution/PHD1_515_targets.csv.png]

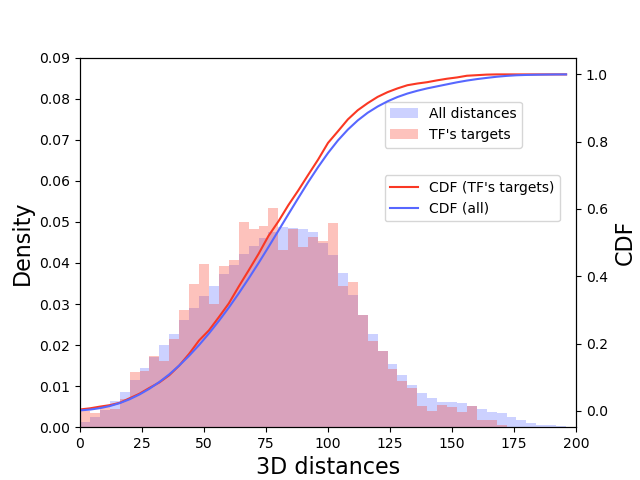

Supplement: Supplementary file 4 — Additional file 4. ZIP file with graphical representations associated to each transcriptional module: (link from Zenodo repository: https://zenodo.org/record/5841177/files/supplementary-data-file-S4.zip?download=1). [file 13104_2022_5940_MOESM4_ESM.zip › 3D_distances_distribution/ARG80_98_targets.csv.png]

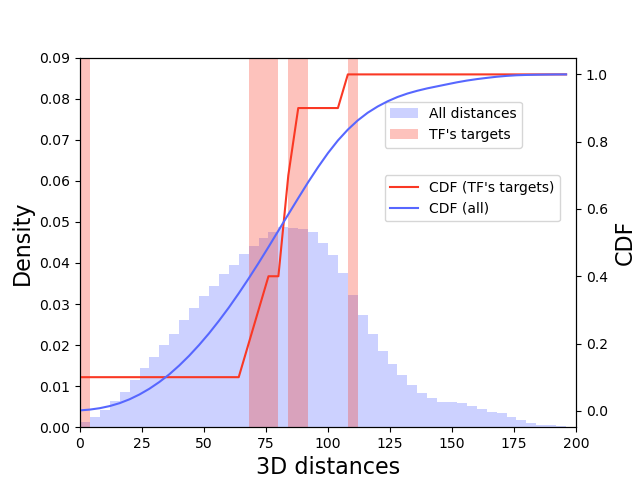

Supplement: Supplementary file 4 — Additional file 4. ZIP file with graphical representations associated to each transcriptional module: (link from Zenodo repository: https://zenodo.org/record/5841177/files/supplementary-data-file-S4.zip?download=1). [file 13104_2022_5940_MOESM4_ESM.zip › 3D_distances_distribution/ZNF1_5_targets.csv.png]

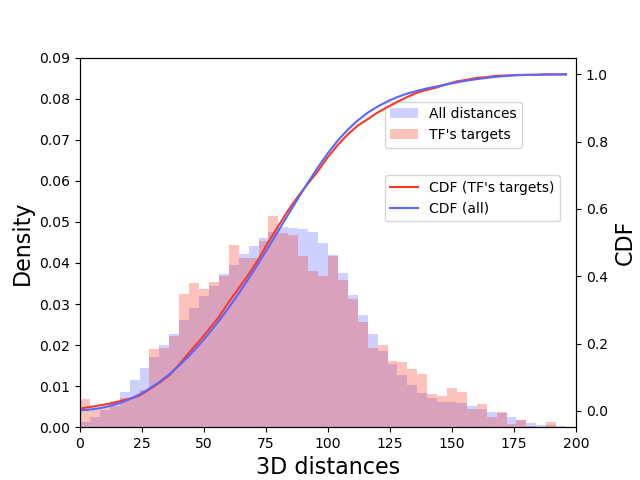

Supplement: Supplementary file 4 — Additional file 4. ZIP file with graphical representations associated to each transcriptional module: (link from Zenodo repository: https://zenodo.org/record/5841177/files/supplementary-data-file-S4.zip?download=1). [file 13104_2022_5940_MOESM4_ESM.zip › 3D_distances_distribution/BAS1_137_targets.csv.png]

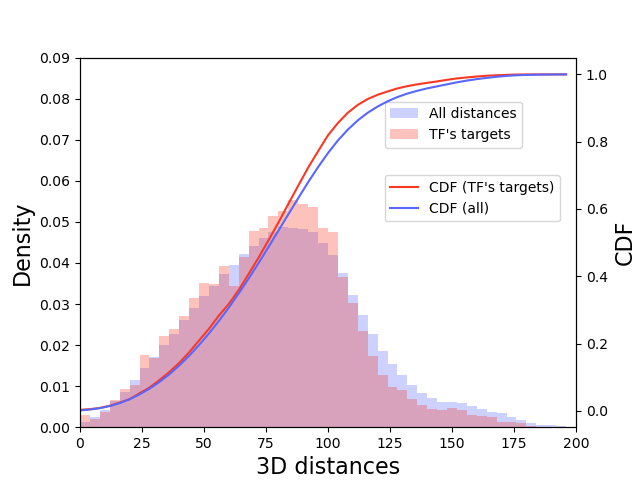

Supplement: Supplementary file 4 — Additional file 4. ZIP file with graphical representations associated to each transcriptional module: (link from Zenodo repository: https://zenodo.org/record/5841177/files/supplementary-data-file-S4.zip?download=1). [file 13104_2022_5940_MOESM4_ESM.zip › 3D_distances_distribution/SFP1_232_targets.csv.png]

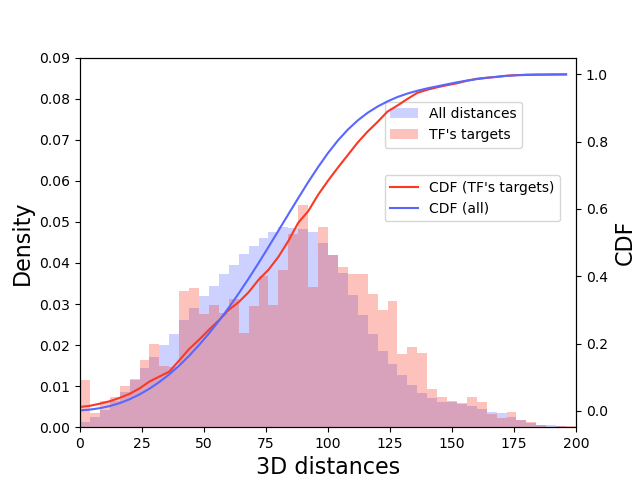

Supplement: Supplementary file 4 — Additional file 4. ZIP file with graphical representations associated to each transcriptional module: (link from Zenodo repository: https://zenodo.org/record/5841177/files/supplementary-data-file-S4.zip?download=1). [file 13104_2022_5940_MOESM4_ESM.zip › 3D_distances_distribution/RPH1_91_targets.csv.png]

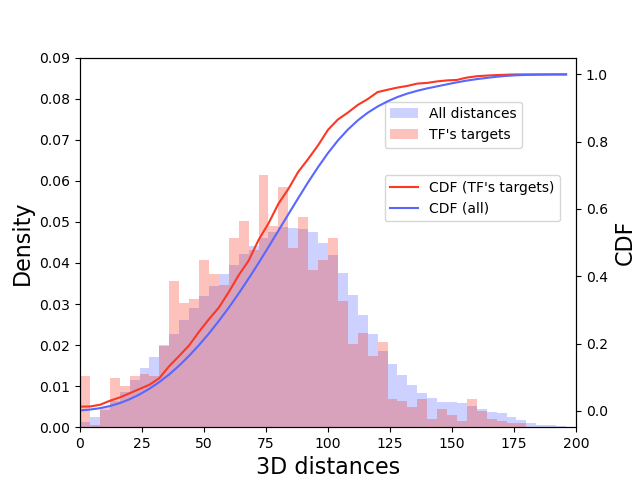

Supplement: Supplementary file 4 — Additional file 4. ZIP file with graphical representations associated to each transcriptional module: (link from Zenodo repository: https://zenodo.org/record/5841177/files/supplementary-data-file-S4.zip?download=1). [file 13104_2022_5940_MOESM4_ESM.zip › 3D_distances_distribution/MSS11_64_targets.csv.png]

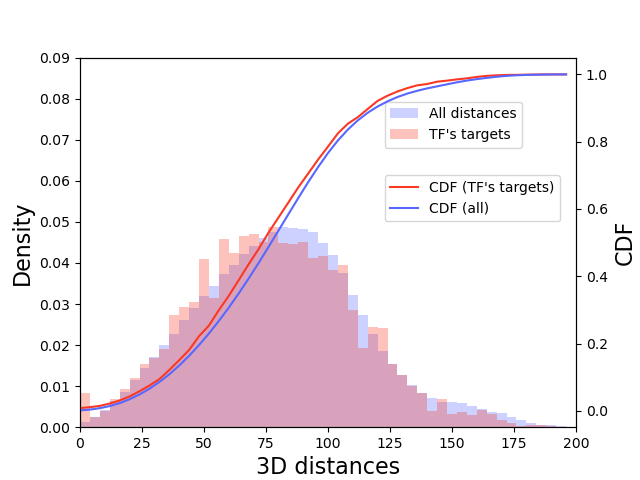

Supplement: Supplementary file 4 — Additional file 4. ZIP file with graphical representations associated to each transcriptional module: (link from Zenodo repository: https://zenodo.org/record/5841177/files/supplementary-data-file-S4.zip?download=1). [file 13104_2022_5940_MOESM4_ESM.zip › 3D_distances_distribution/SUM1_129_targets.csv.png]

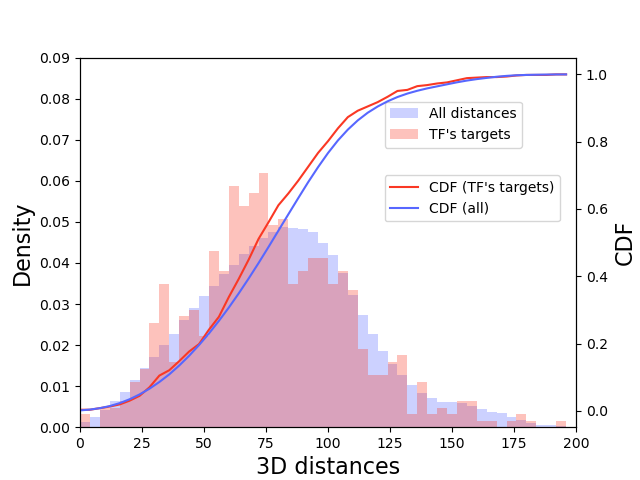

Supplement: Supplementary file 4 — Additional file 4. ZIP file with graphical representations associated to each transcriptional module: (link from Zenodo repository: https://zenodo.org/record/5841177/files/supplementary-data-file-S4.zip?download=1). [file 13104_2022_5940_MOESM4_ESM.zip › 3D_distances_distribution/SFL1_36_targets.csv.png]

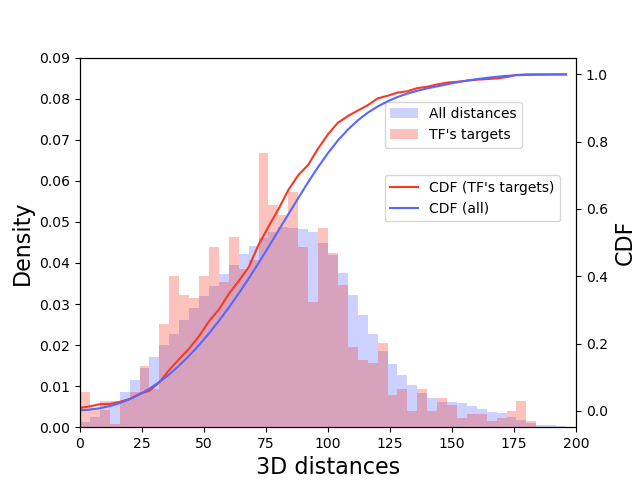

Supplement: Supplementary file 4 — Additional file 4. ZIP file with graphical representations associated to each transcriptional module: (link from Zenodo repository: https://zenodo.org/record/5841177/files/supplementary-data-file-S4.zip?download=1). [file 13104_2022_5940_MOESM4_ESM.zip › 3D_distances_distribution/RGT1_51_targets.csv.png]

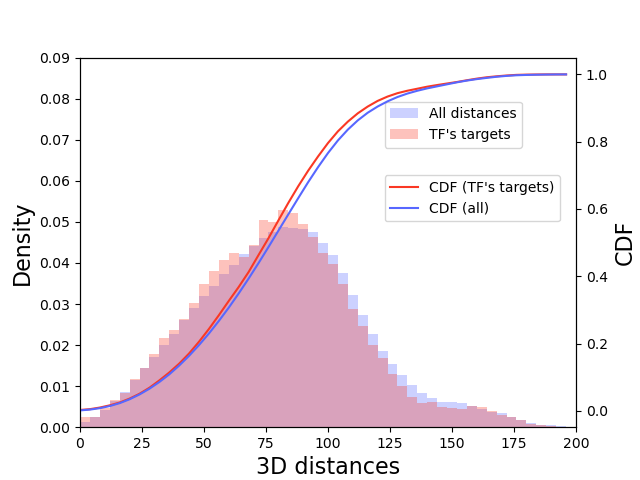

Supplement: Supplementary file 4 — Additional file 4. ZIP file with graphical representations associated to each transcriptional module: (link from Zenodo repository: https://zenodo.org/record/5841177/files/supplementary-data-file-S4.zip?download=1). [file 13104_2022_5940_MOESM4_ESM.zip › 3D_distances_distribution/HAP1_487_targets.csv.png]

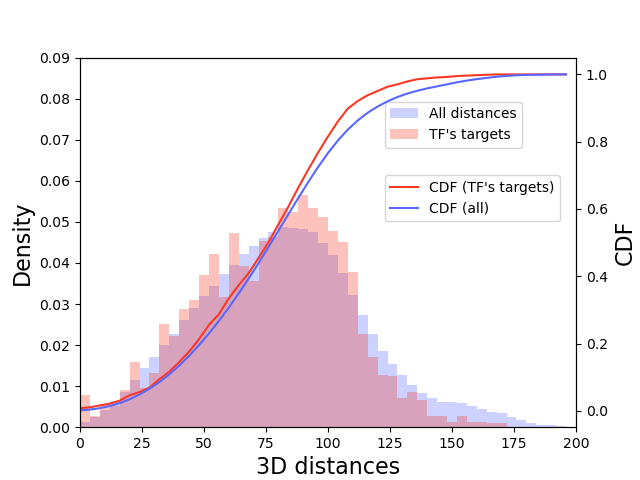

Supplement: Supplementary file 4 — Additional file 4. ZIP file with graphical representations associated to each transcriptional module: (link from Zenodo repository: https://zenodo.org/record/5841177/files/supplementary-data-file-S4.zip?download=1). [file 13104_2022_5940_MOESM4_ESM.zip › 3D_distances_distribution/CHA4_95_targets.csv.png]

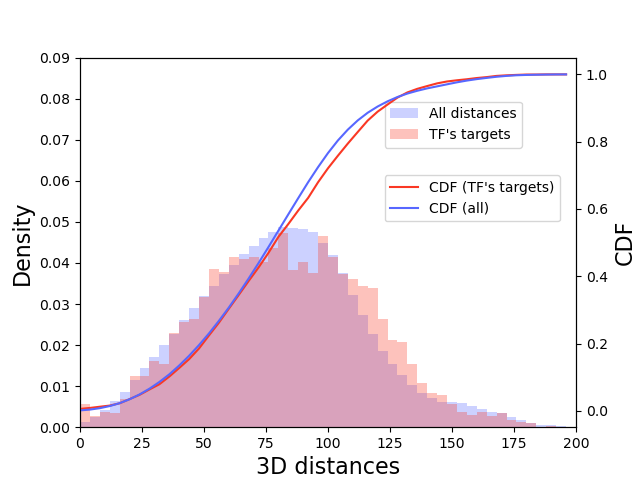

Supplement: Supplementary file 4 — Additional file 4. ZIP file with graphical representations associated to each transcriptional module: (link from Zenodo repository: https://zenodo.org/record/5841177/files/supplementary-data-file-S4.zip?download=1). [file 13104_2022_5940_MOESM4_ESM.zip › 3D_distances_distribution/HAP4_202_targets.csv.png]

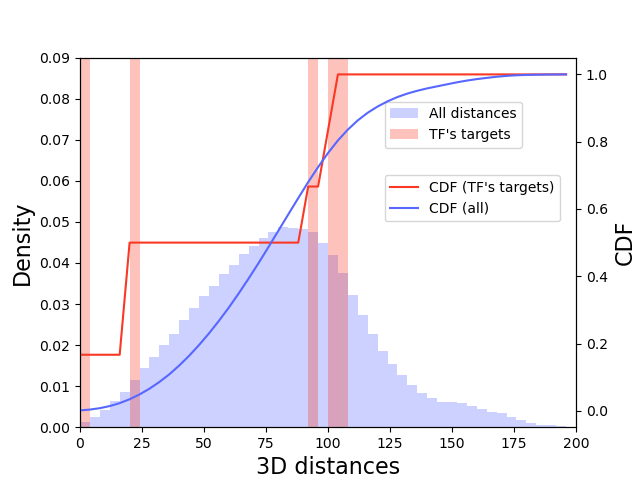

Supplement: Supplementary file 4 — Additional file 4. ZIP file with graphical representations associated to each transcriptional module: (link from Zenodo repository: https://zenodo.org/record/5841177/files/supplementary-data-file-S4.zip?download=1). [file 13104_2022_5940_MOESM4_ESM.zip › 3D_distances_distribution/GAL3_4_targets.csv.png]

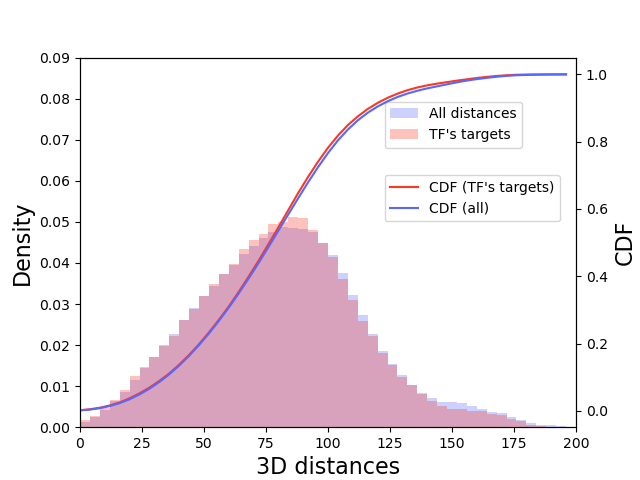

Supplement: Supplementary file 4 — Additional file 4. ZIP file with graphical representations associated to each transcriptional module: (link from Zenodo repository: https://zenodo.org/record/5841177/files/supplementary-data-file-S4.zip?download=1). [file 13104_2022_5940_MOESM4_ESM.zip › 3D_distances_distribution/INO4_1062_targets.csv.png]

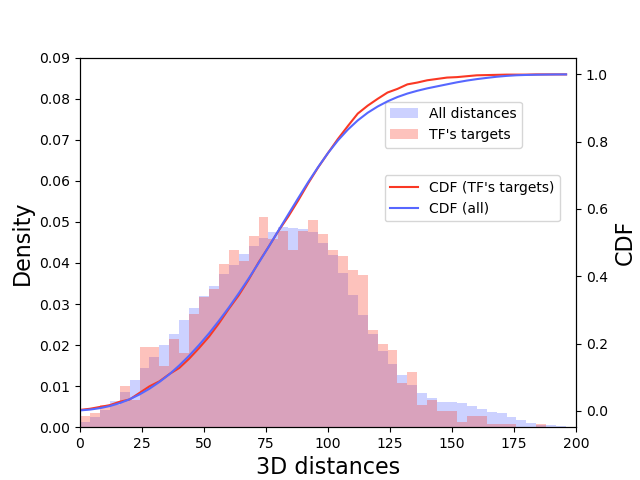

Supplement: Supplementary file 4 — Additional file 4. ZIP file with graphical representations associated to each transcriptional module: (link from Zenodo repository: https://zenodo.org/record/5841177/files/supplementary-data-file-S4.zip?download=1). [file 13104_2022_5940_MOESM4_ESM.zip › 3D_distances_distribution/SRD1_55_targets.csv.png]

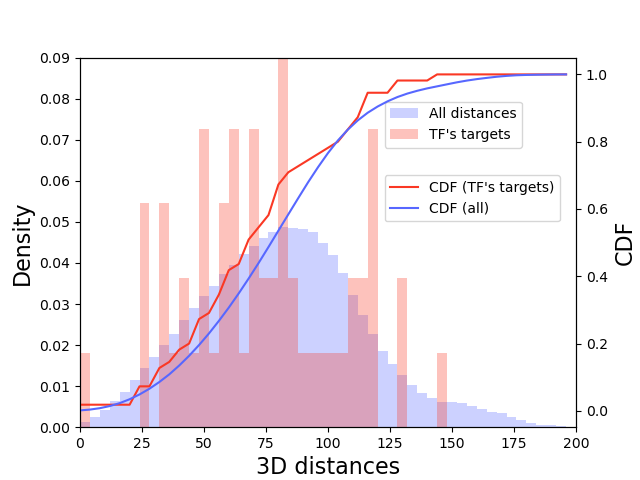

Supplement: Supplementary file 4 — Additional file 4. ZIP file with graphical representations associated to each transcriptional module: (link from Zenodo repository: https://zenodo.org/record/5841177/files/supplementary-data-file-S4.zip?download=1). [file 13104_2022_5940_MOESM4_ESM.zip › 3D_distances_distribution/IME1_11_targets.csv.png]

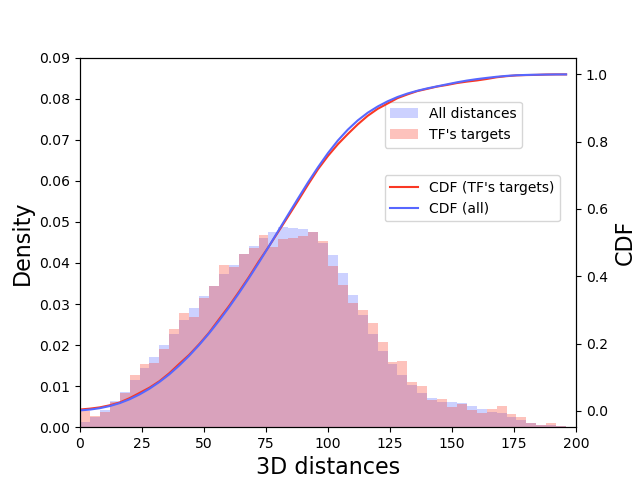

Supplement: Supplementary file 4 — Additional file 4. ZIP file with graphical representations associated to each transcriptional module: (link from Zenodo repository: https://zenodo.org/record/5841177/files/supplementary-data-file-S4.zip?download=1). [file 13104_2022_5940_MOESM4_ESM.zip › 3D_distances_distribution/PHO4_287_targets.csv.png]

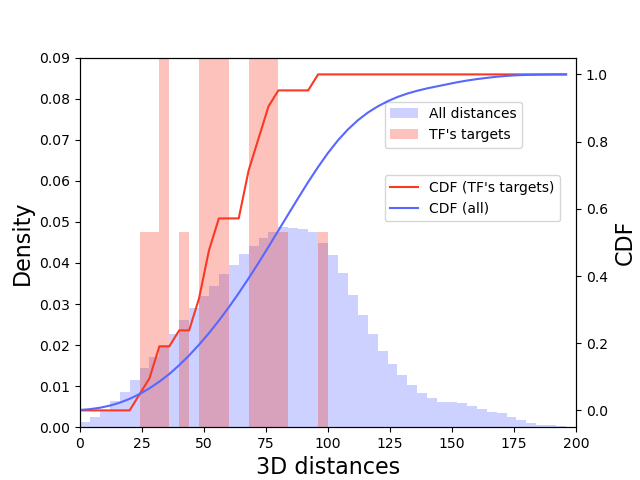

Supplement: Supplementary file 4 — Additional file 4. ZIP file with graphical representations associated to each transcriptional module: (link from Zenodo repository: https://zenodo.org/record/5841177/files/supplementary-data-file-S4.zip?download=1). [file 13104_2022_5940_MOESM4_ESM.zip › 3D_distances_distribution/HAA1_7_targets.csv.png]
