# Supplementary material for: Additional insights into the organization of transcriptional regulatory modules based on a 3D model of the Saccharomyces cerevisiae genome
Source: BMC Res Notes. 2022 Feb 19;15:67. doi: 10.1186/s13104-022-05940-5 (PMC8858486; doi:10.1186/s13104-022-05940-5)
Supplement: Supplementary file 2 — Additional file 2. Text file describing the method that was applied to associate the 9185 S. cerevisiae chromosomal features with the spatial coordinates of the 3D model. [file 13104_2022_5940_MOESM2_ESM.pdf]

Supplementary file S2 : Method to associate the 9,185 *S. cerevisiae* chromosomal features with the spatial coordinates of the 3D model

We recovered the 3D model from the study of Duan *et al.* (supplementary data). In the related supplementary data, we found the 3D coordinates for 26,538 points. Chromosomes are modeled by a sequence of points in space. First, we measured the lengths of the segments connecting these points by a calculation of the Euclidean distance. Their sizes on the chromosome are obtained with the following calculation: (length of the segment * length of the chromosome) / sums of the lengths of the segments constituting this chromosome. A chromosome is thus divided into a set of segments of variable sizes. Indeed, points in the initial model are not equidistant. We observed that when a chromosome changes its direction in space, more points were used to model the same length in pb (hence they are closer from one another). We then retrieved 9,185 *S. cerevisiae* genome features from the SQL database. Each feature is associated with all the segments intersecting it. Each segment is therefore associated with a list of features. In order to represent all the features in space, segments had to be subdivided. New segments were associated with only one feature. If a feature was associated with several segments, it was taken into account only for the first segment's subdivision. One segment was divided into new segments all equal in length: initial segment length / number of features associated with the initial segment. The distance matrix was calculated by the Euclidean distance between positions of the 9,185 features studied. Features were summarized with the beginning point of their associated segment. Each feature was thus associated with three coordinates in space.
