# Supplementary figures and images for: Additional insights into the organization of transcriptional regulatory modules based on a 3D model of the Saccharomyces cerevisiae genome (part 2 of 2)
Source: BMC Res Notes. 2022 Feb 19;15:67. doi: 10.1186/s13104-022-05940-5 (PMC8858486; doi:10.1186/s13104-022-05940-5)

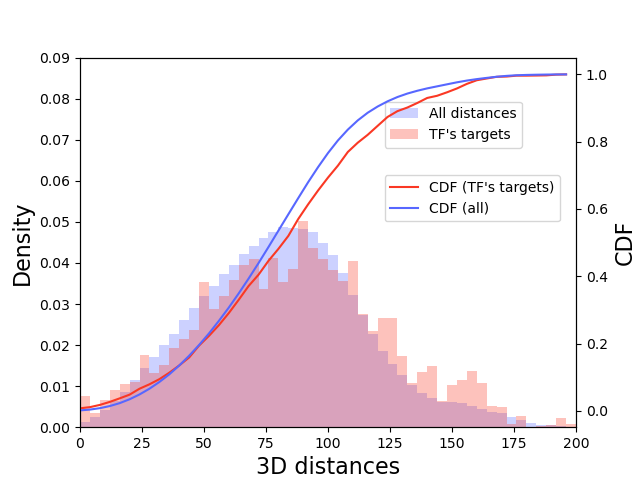

Supplement: Supplementary file 4 — Additional file 4. ZIP file with graphical representations associated to each transcriptional module: (link from Zenodo repository: https://zenodo.org/record/5841177/files/supplementary-data-file-S4.zip?download=1). [file 13104_2022_5940_MOESM4_ESM.zip › 3D_distances_distribution/PUT3_143_targets.csv.png]

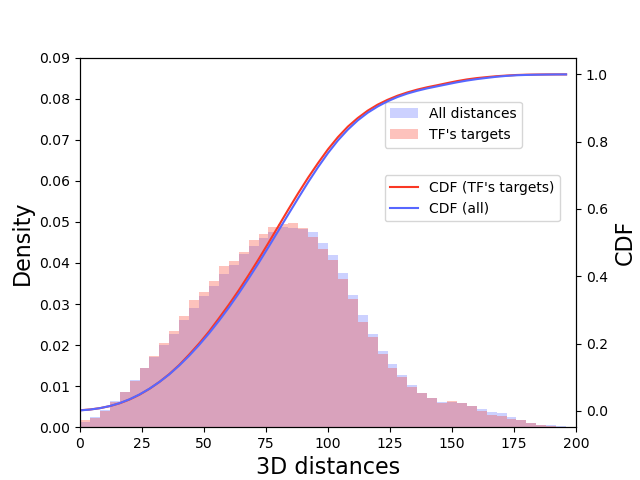

Supplement: Supplementary file 4 — Additional file 4. ZIP file with graphical representations associated to each transcriptional module: (link from Zenodo repository: https://zenodo.org/record/5841177/files/supplementary-data-file-S4.zip?download=1). [file 13104_2022_5940_MOESM4_ESM.zip › 3D_distances_distribution/RAP1_1551_targets.csv.png]

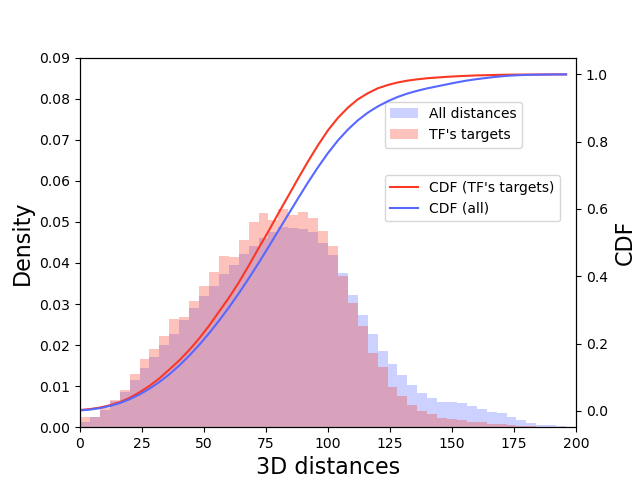

Supplement: Supplementary file 4 — Additional file 4. ZIP file with graphical representations associated to each transcriptional module: (link from Zenodo repository: https://zenodo.org/record/5841177/files/supplementary-data-file-S4.zip?download=1). [file 13104_2022_5940_MOESM4_ESM.zip › 3D_distances_distribution/GCN4_541_targets.csv.png]

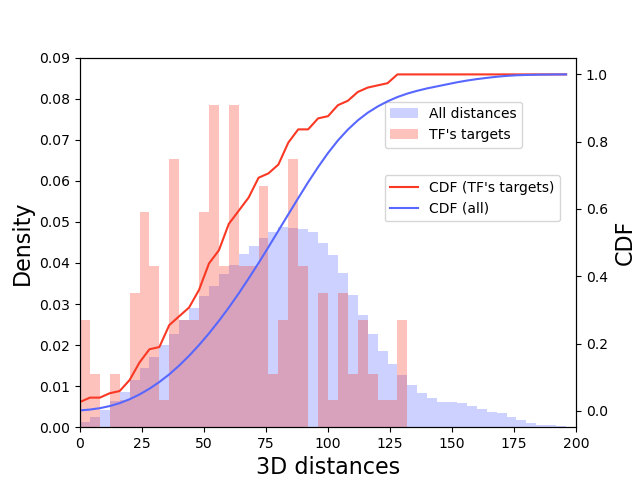

Supplement: Supplementary file 4 — Additional file 4. ZIP file with graphical representations associated to each transcriptional module: (link from Zenodo repository: https://zenodo.org/record/5841177/files/supplementary-data-file-S4.zip?download=1). [file 13104_2022_5940_MOESM4_ESM.zip › 3D_distances_distribution/HAC1_18_targets.csv.png]

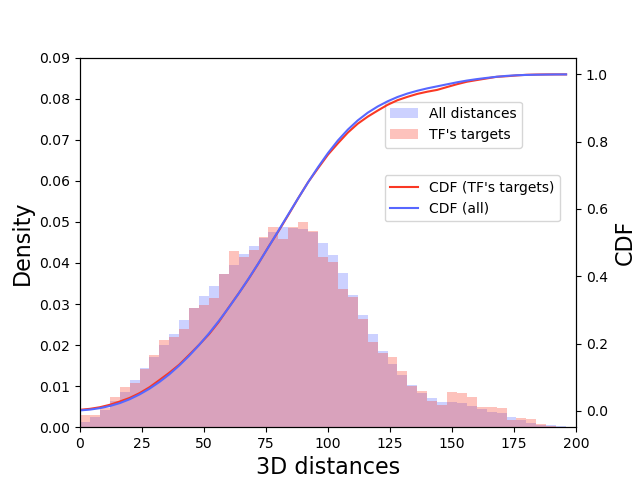

Supplement: Supplementary file 4 — Additional file 4. ZIP file with graphical representations associated to each transcriptional module: (link from Zenodo repository: https://zenodo.org/record/5841177/files/supplementary-data-file-S4.zip?download=1). [file 13104_2022_5940_MOESM4_ESM.zip › 3D_distances_distribution/YAP1_312_targets.csv.png]

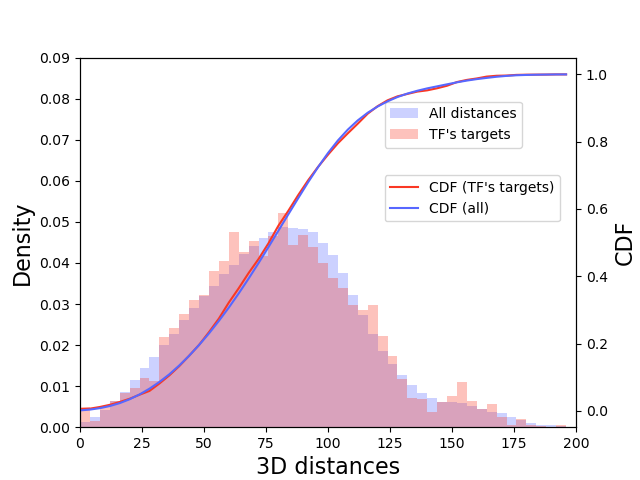

Supplement: Supplementary file 4 — Additional file 4. ZIP file with graphical representations associated to each transcriptional module: (link from Zenodo repository: https://zenodo.org/record/5841177/files/supplementary-data-file-S4.zip?download=1). [file 13104_2022_5940_MOESM4_ESM.zip › 3D_distances_distribution/AFT2_167_targets.csv.png]

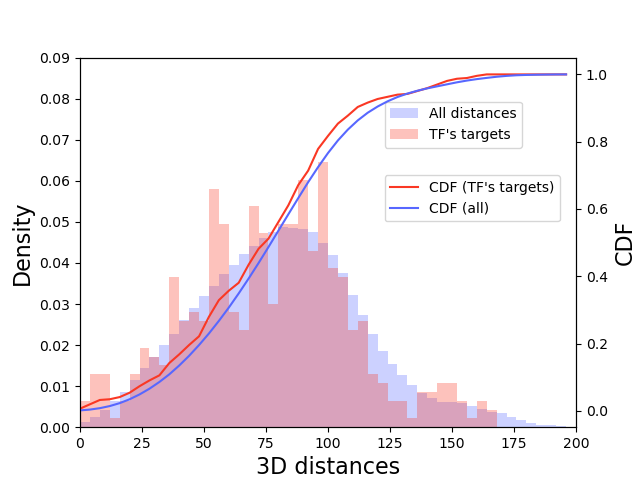

Supplement: Supplementary file 4 — Additional file 4. ZIP file with graphical representations associated to each transcriptional module: (link from Zenodo repository: https://zenodo.org/record/5841177/files/supplementary-data-file-S4.zip?download=1). [file 13104_2022_5940_MOESM4_ESM.zip › 3D_distances_distribution/MOT2_31_targets.csv.png]

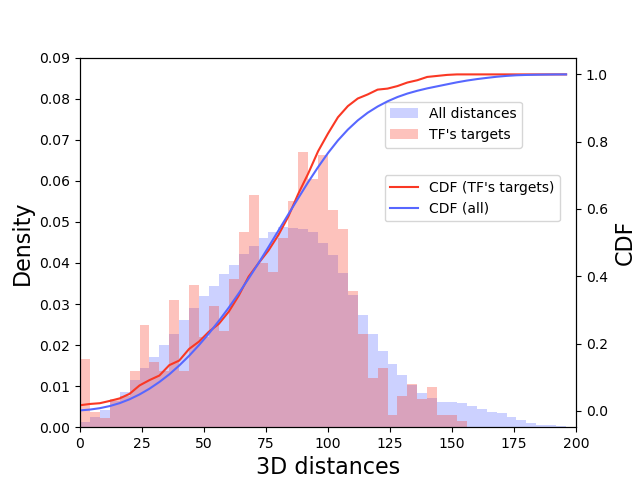

Supplement: Supplementary file 4 — Additional file 4. ZIP file with graphical representations associated to each transcriptional module: (link from Zenodo repository: https://zenodo.org/record/5841177/files/supplementary-data-file-S4.zip?download=1). [file 13104_2022_5940_MOESM4_ESM.zip › 3D_distances_distribution/YJL206C_52_targets.csv.png]

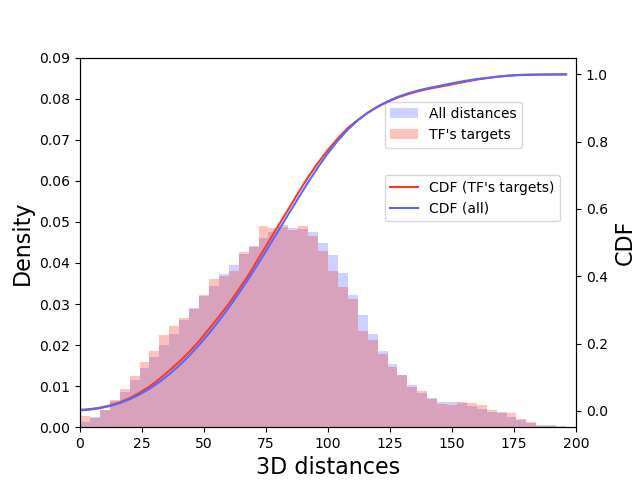

Supplement: Supplementary file 4 — Additional file 4. ZIP file with graphical representations associated to each transcriptional module: (link from Zenodo repository: https://zenodo.org/record/5841177/files/supplementary-data-file-S4.zip?download=1). [file 13104_2022_5940_MOESM4_ESM.zip › 3D_distances_distribution/SKN7_555_targets.csv.png]

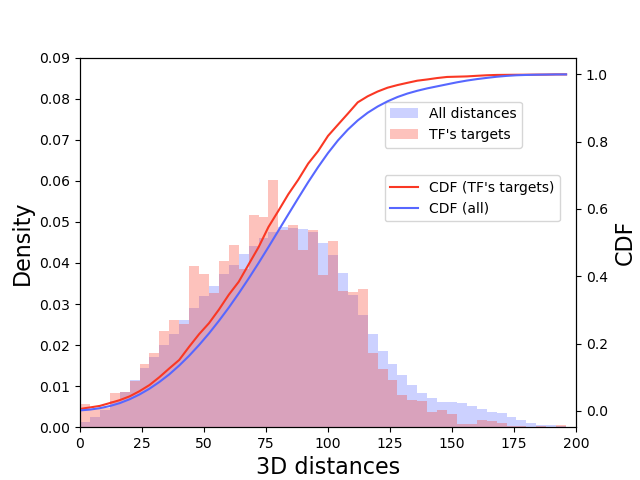

Supplement: Supplementary file 4 — Additional file 4. ZIP file with graphical representations associated to each transcriptional module: (link from Zenodo repository: https://zenodo.org/record/5841177/files/supplementary-data-file-S4.zip?download=1). [file 13104_2022_5940_MOESM4_ESM.zip › 3D_distances_distribution/TYE7_122_targets.csv.png]

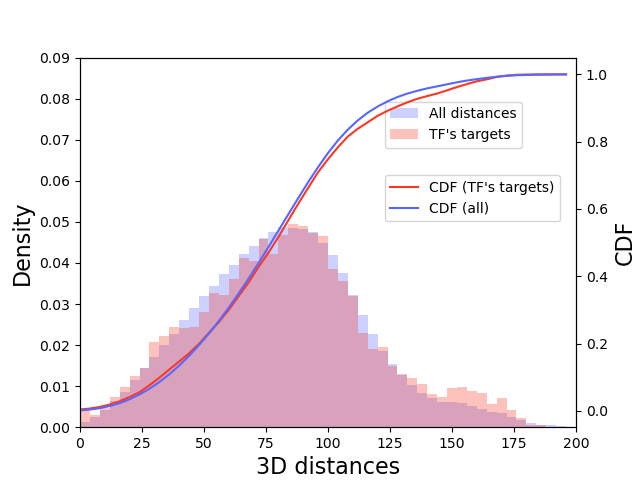

Supplement: Supplementary file 4 — Additional file 4. ZIP file with graphical representations associated to each transcriptional module: (link from Zenodo repository: https://zenodo.org/record/5841177/files/supplementary-data-file-S4.zip?download=1). [file 13104_2022_5940_MOESM4_ESM.zip › 3D_distances_distribution/DAL81_244_targets.csv.png]

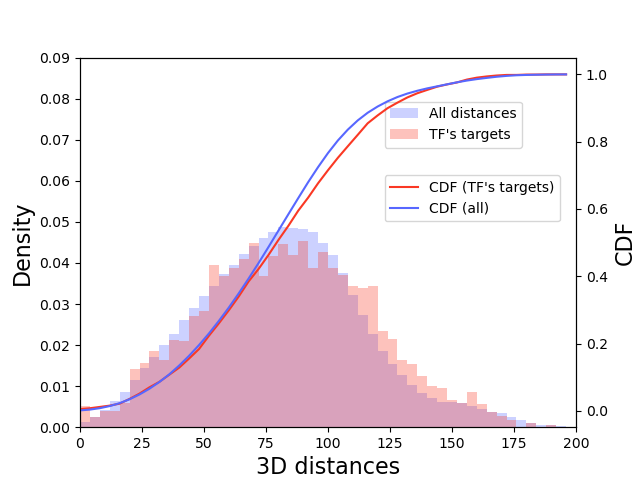

Supplement: Supplementary file 4 — Additional file 4. ZIP file with graphical representations associated to each transcriptional module: (link from Zenodo repository: https://zenodo.org/record/5841177/files/supplementary-data-file-S4.zip?download=1). [file 13104_2022_5940_MOESM4_ESM.zip › 3D_distances_distribution/HAP5_173_targets.csv.png]

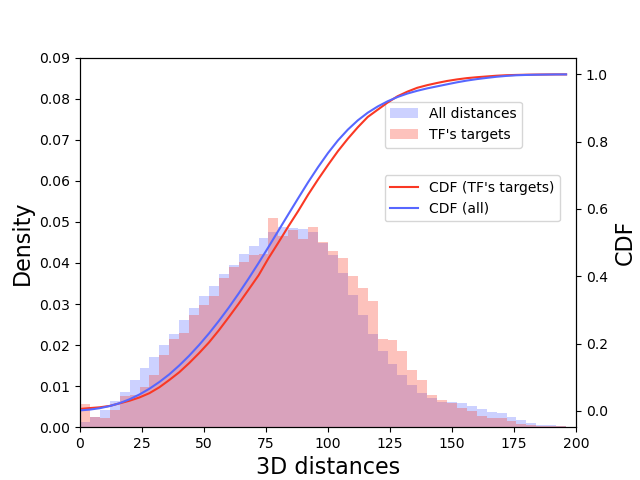

Supplement: Supplementary file 4 — Additional file 4. ZIP file with graphical representations associated to each transcriptional module: (link from Zenodo repository: https://zenodo.org/record/5841177/files/supplementary-data-file-S4.zip?download=1). [file 13104_2022_5940_MOESM4_ESM.zip › 3D_distances_distribution/SMP1_223_targets.csv.png]

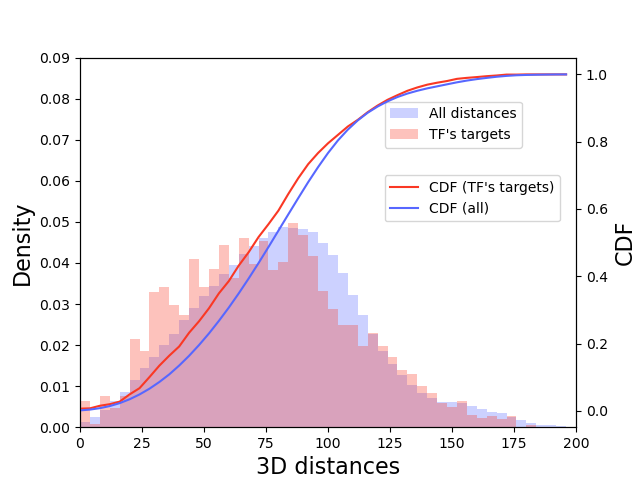

Supplement: Supplementary file 4 — Additional file 4. ZIP file with graphical representations associated to each transcriptional module: (link from Zenodo repository: https://zenodo.org/record/5841177/files/supplementary-data-file-S4.zip?download=1). [file 13104_2022_5940_MOESM4_ESM.zip › 3D_distances_distribution/MSN1_83_targets.csv.png]

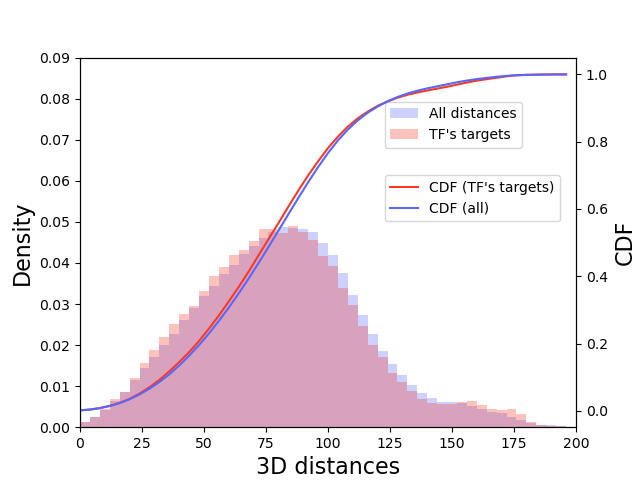

Supplement: Supplementary file 4 — Additional file 4. ZIP file with graphical representations associated to each transcriptional module: (link from Zenodo repository: https://zenodo.org/record/5841177/files/supplementary-data-file-S4.zip?download=1). [file 13104_2022_5940_MOESM4_ESM.zip › 3D_distances_distribution/IXR1_493_targets.csv.png]

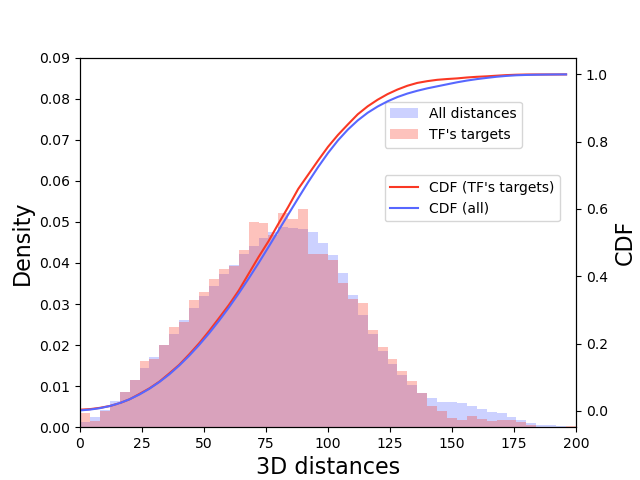

Supplement: Supplementary file 4 — Additional file 4. ZIP file with graphical representations associated to each transcriptional module: (link from Zenodo repository: https://zenodo.org/record/5841177/files/supplementary-data-file-S4.zip?download=1). [file 13104_2022_5940_MOESM4_ESM.zip › 3D_distances_distribution/RDS2_200_targets.csv.png]

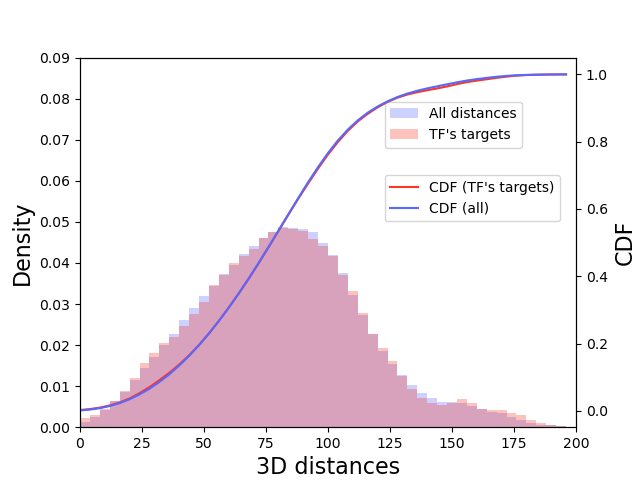

Supplement: Supplementary file 4 — Additional file 4. ZIP file with graphical representations associated to each transcriptional module: (link from Zenodo repository: https://zenodo.org/record/5841177/files/supplementary-data-file-S4.zip?download=1). [file 13104_2022_5940_MOESM4_ESM.zip › 3D_distances_distribution/CBF1_751_targets.csv.png]

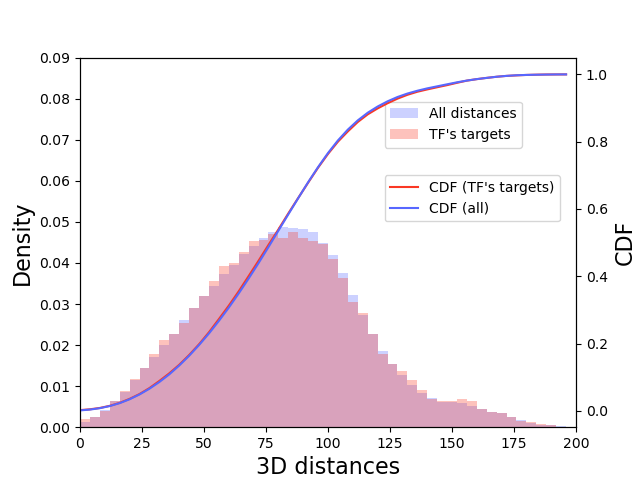

Supplement: Supplementary file 4 — Additional file 4. ZIP file with graphical representations associated to each transcriptional module: (link from Zenodo repository: https://zenodo.org/record/5841177/files/supplementary-data-file-S4.zip?download=1). [file 13104_2022_5940_MOESM4_ESM.zip › 3D_distances_distribution/YRR1_655_targets.csv.png]

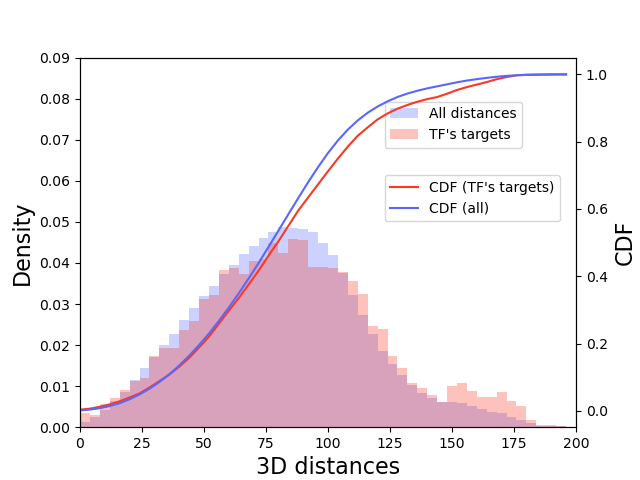

Supplement: Supplementary file 4 — Additional file 4. ZIP file with graphical representations associated to each transcriptional module: (link from Zenodo repository: https://zenodo.org/record/5841177/files/supplementary-data-file-S4.zip?download=1). [file 13104_2022_5940_MOESM4_ESM.zip › 3D_distances_distribution/RTG3_185_targets.csv.png]

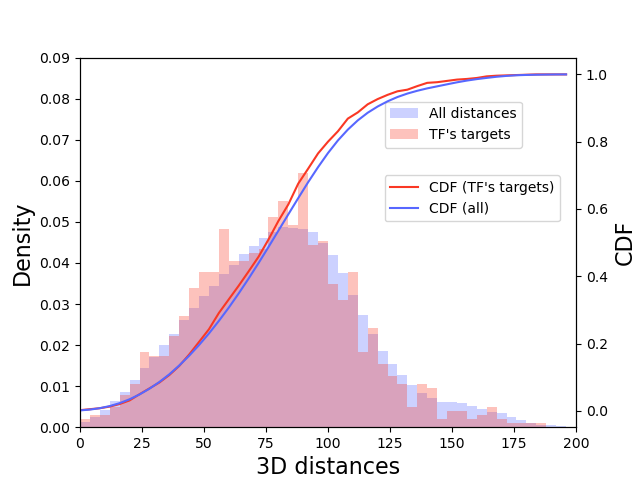

Supplement: Supplementary file 4 — Additional file 4. ZIP file with graphical representations associated to each transcriptional module: (link from Zenodo repository: https://zenodo.org/record/5841177/files/supplementary-data-file-S4.zip?download=1). [file 13104_2022_5940_MOESM4_ESM.zip › 3D_distances_distribution/USV1_46_targets.csv.png]

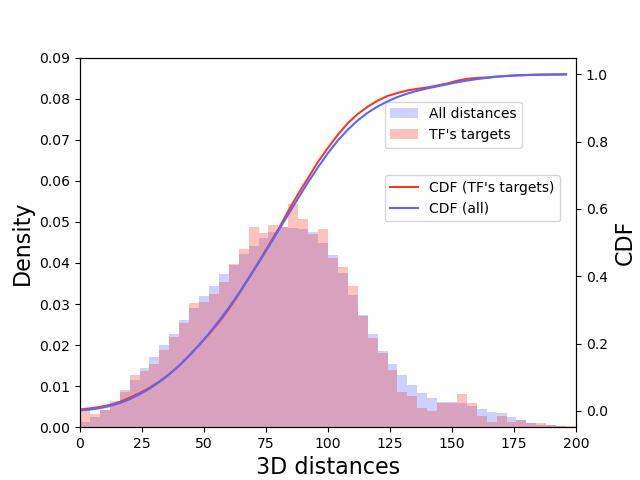

Supplement: Supplementary file 4 — Additional file 4. ZIP file with graphical representations associated to each transcriptional module: (link from Zenodo repository: https://zenodo.org/record/5841177/files/supplementary-data-file-S4.zip?download=1). [file 13104_2022_5940_MOESM4_ESM.zip › 3D_distances_distribution/PIP2_272_targets.csv.png]

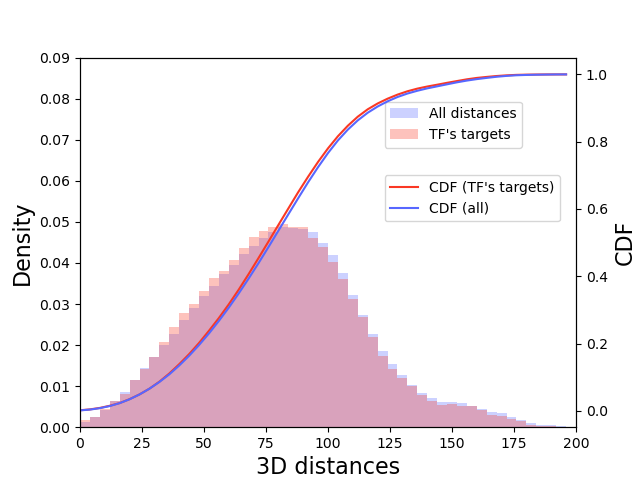

Supplement: Supplementary file 4 — Additional file 4. ZIP file with graphical representations associated to each transcriptional module: (link from Zenodo repository: https://zenodo.org/record/5841177/files/supplementary-data-file-S4.zip?download=1). [file 13104_2022_5940_MOESM4_ESM.zip › 3D_distances_distribution/YAP6_910_targets.csv.png]

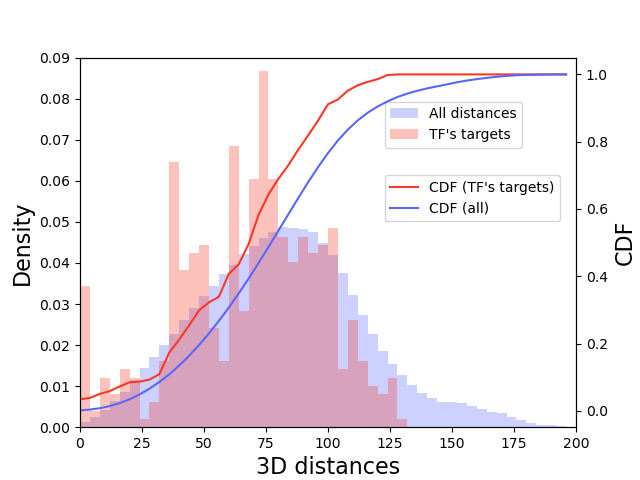

Supplement: Supplementary file 4 — Additional file 4. ZIP file with graphical representations associated to each transcriptional module: (link from Zenodo repository: https://zenodo.org/record/5841177/files/supplementary-data-file-S4.zip?download=1). [file 13104_2022_5940_MOESM4_ESM.zip › 3D_distances_distribution/STB4_32_targets.csv.png]

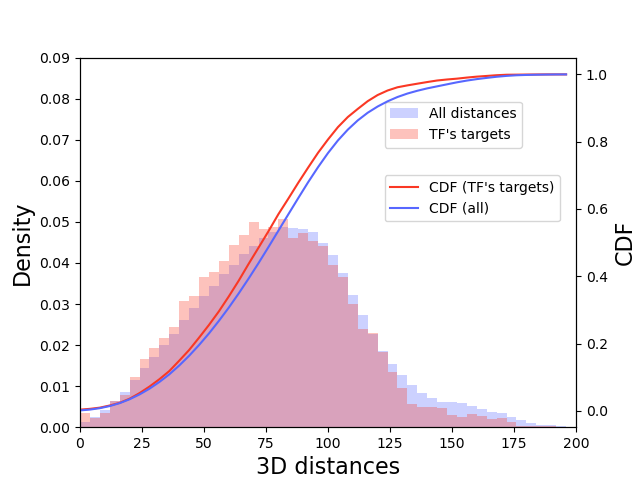

Supplement: Supplementary file 4 — Additional file 4. ZIP file with graphical representations associated to each transcriptional module: (link from Zenodo repository: https://zenodo.org/record/5841177/files/supplementary-data-file-S4.zip?download=1). [file 13104_2022_5940_MOESM4_ESM.zip › 3D_distances_distribution/TOS8_309_targets.csv.png]

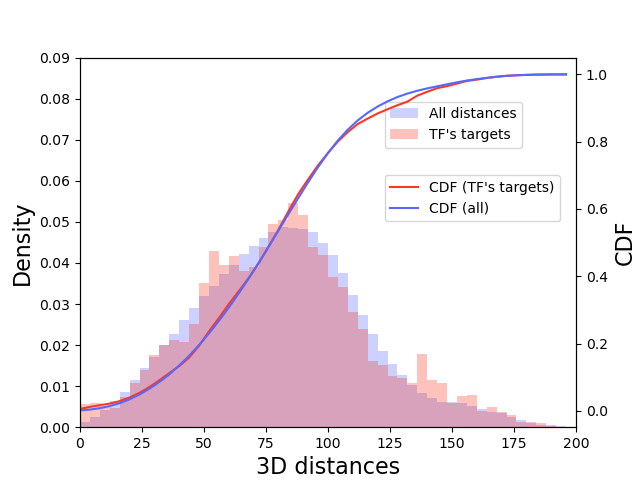

Supplement: Supplementary file 4 — Additional file 4. ZIP file with graphical representations associated to each transcriptional module: (link from Zenodo repository: https://zenodo.org/record/5841177/files/supplementary-data-file-S4.zip?download=1). [file 13104_2022_5940_MOESM4_ESM.zip › 3D_distances_distribution/DAL82_162_targets.csv.png]

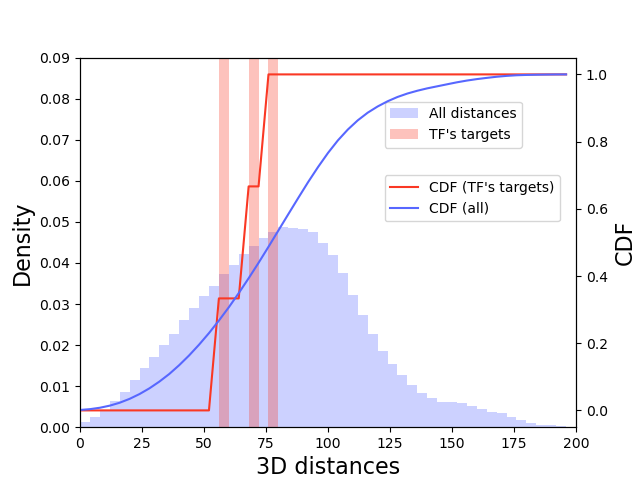

Supplement: Supplementary file 4 — Additional file 4. ZIP file with graphical representations associated to each transcriptional module: (link from Zenodo repository: https://zenodo.org/record/5841177/files/supplementary-data-file-S4.zip?download=1). [file 13104_2022_5940_MOESM4_ESM.zip › 3D_distances_distribution/KAR4_3_targets.csv.png]

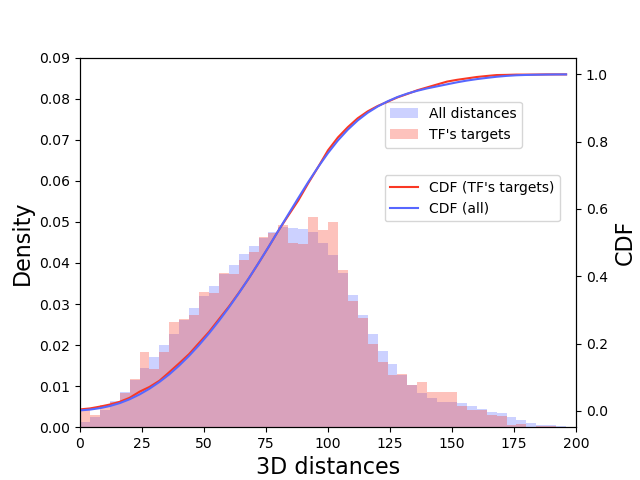

Supplement: Supplementary file 4 — Additional file 4. ZIP file with graphical representations associated to each transcriptional module: (link from Zenodo repository: https://zenodo.org/record/5841177/files/supplementary-data-file-S4.zip?download=1). [file 13104_2022_5940_MOESM4_ESM.zip › 3D_distances_distribution/ECM22_140_targets.csv.png]

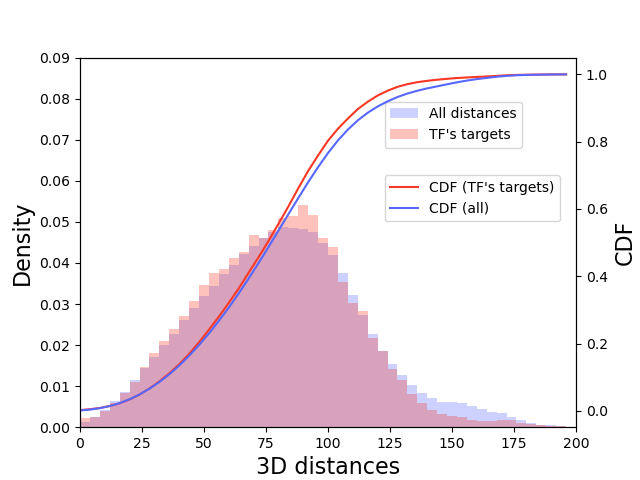

Supplement: Supplementary file 4 — Additional file 4. ZIP file with graphical representations associated to each transcriptional module: (link from Zenodo repository: https://zenodo.org/record/5841177/files/supplementary-data-file-S4.zip?download=1). [file 13104_2022_5940_MOESM4_ESM.zip › 3D_distances_distribution/YOX1_475_targets.csv.png]

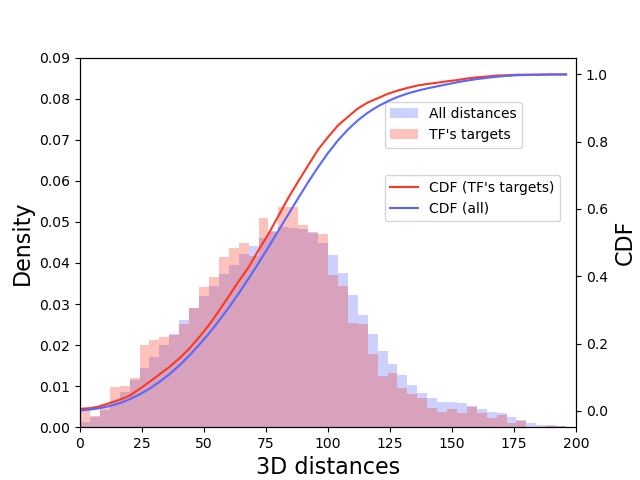

Supplement: Supplementary file 4 — Additional file 4. ZIP file with graphical representations associated to each transcriptional module: (link from Zenodo repository: https://zenodo.org/record/5841177/files/supplementary-data-file-S4.zip?download=1). [file 13104_2022_5940_MOESM4_ESM.zip › 3D_distances_distribution/GLN3_157_targets.csv.png]

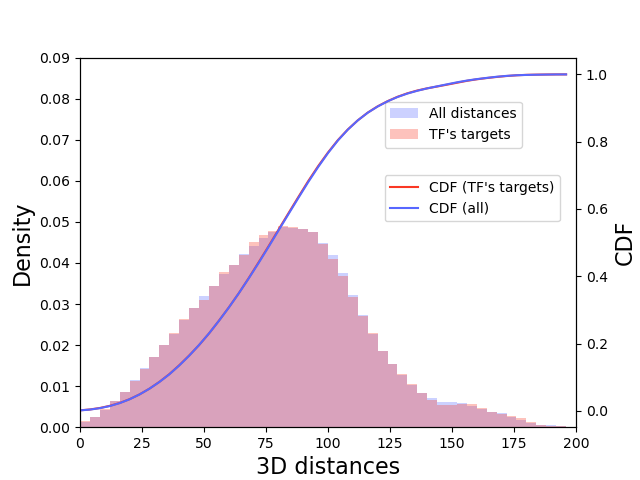

Supplement: Supplementary file 4 — Additional file 4. ZIP file with graphical representations associated to each transcriptional module: (link from Zenodo repository: https://zenodo.org/record/5841177/files/supplementary-data-file-S4.zip?download=1). [file 13104_2022_5940_MOESM4_ESM.zip › 3D_distances_distribution/MSN2_1854_targets.csv.png]

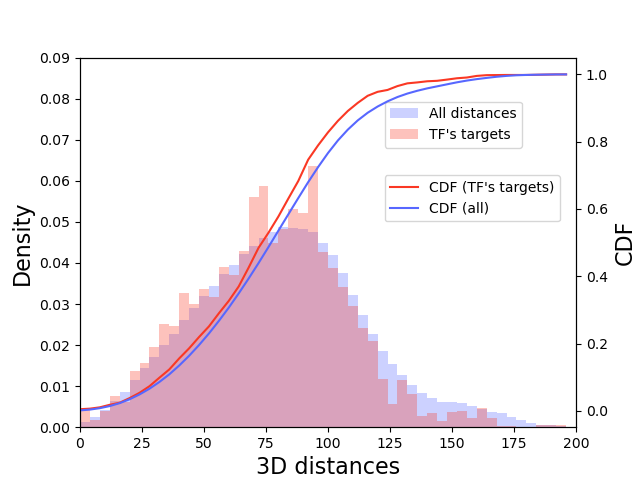

Supplement: Supplementary file 4 — Additional file 4. ZIP file with graphical representations associated to each transcriptional module: (link from Zenodo repository: https://zenodo.org/record/5841177/files/supplementary-data-file-S4.zip?download=1). [file 13104_2022_5940_MOESM4_ESM.zip › 3D_distances_distribution/GCR1_85_targets.csv.png]

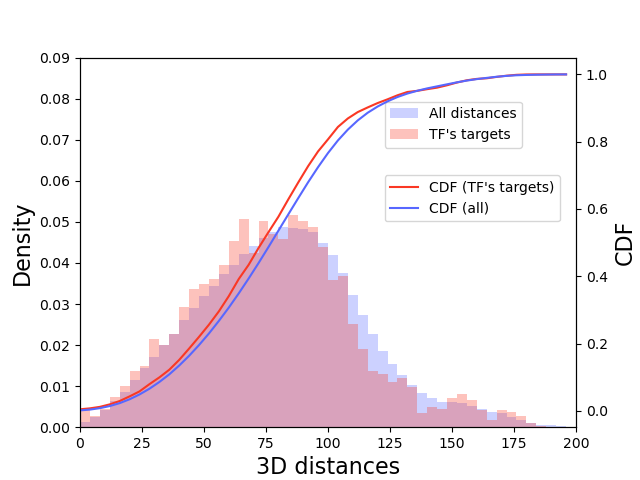

Supplement: Supplementary file 4 — Additional file 4. ZIP file with graphical representations associated to each transcriptional module: (link from Zenodo repository: https://zenodo.org/record/5841177/files/supplementary-data-file-S4.zip?download=1). [file 13104_2022_5940_MOESM4_ESM.zip › 3D_distances_distribution/YAP7_180_targets.csv.png]

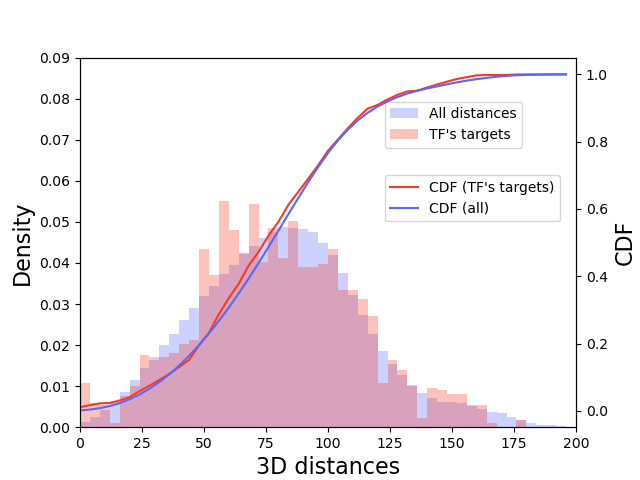

Supplement: Supplementary file 4 — Additional file 4. ZIP file with graphical representations associated to each transcriptional module: (link from Zenodo repository: https://zenodo.org/record/5841177/files/supplementary-data-file-S4.zip?download=1). [file 13104_2022_5940_MOESM4_ESM.zip › 3D_distances_distribution/THI2_67_targets.csv.png]

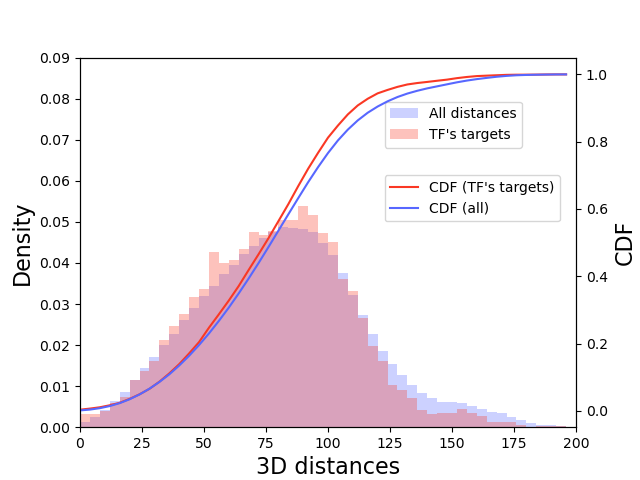

Supplement: Supplementary file 4 — Additional file 4. ZIP file with graphical representations associated to each transcriptional module: (link from Zenodo repository: https://zenodo.org/record/5841177/files/supplementary-data-file-S4.zip?download=1). [file 13104_2022_5940_MOESM4_ESM.zip › 3D_distances_distribution/HCM1_318_targets.csv.png]

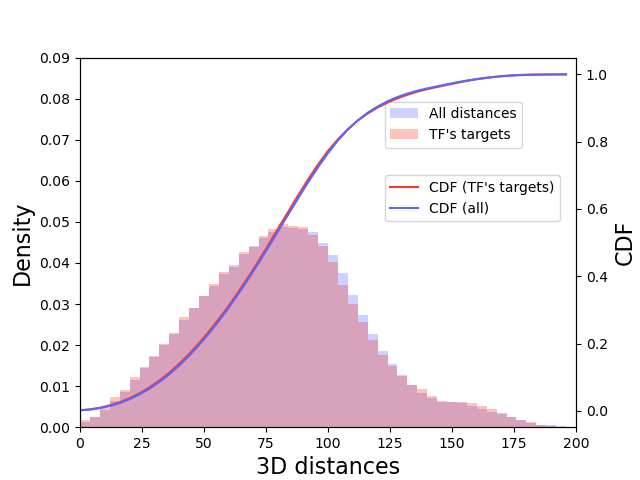

Supplement: Supplementary file 4 — Additional file 4. ZIP file with graphical representations associated to each transcriptional module: (link from Zenodo repository: https://zenodo.org/record/5841177/files/supplementary-data-file-S4.zip?download=1). [file 13104_2022_5940_MOESM4_ESM.zip › 3D_distances_distribution/FKH2_1689_targets.csv.png]

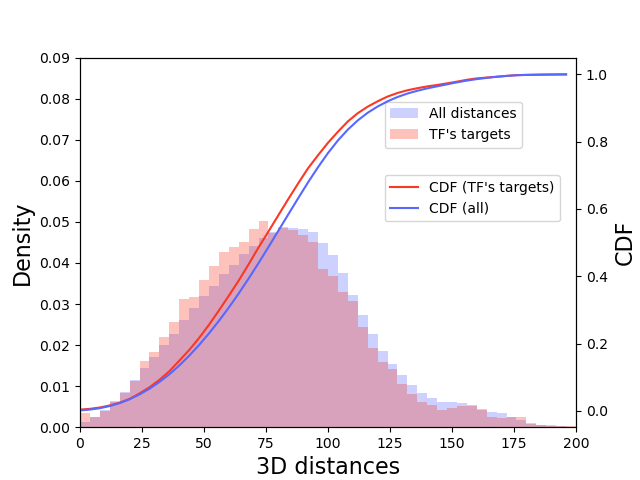

Supplement: Supplementary file 4 — Additional file 4. ZIP file with graphical representations associated to each transcriptional module: (link from Zenodo repository: https://zenodo.org/record/5841177/files/supplementary-data-file-S4.zip?download=1). [file 13104_2022_5940_MOESM4_ESM.zip › 3D_distances_distribution/FLO8_323_targets.csv.png]

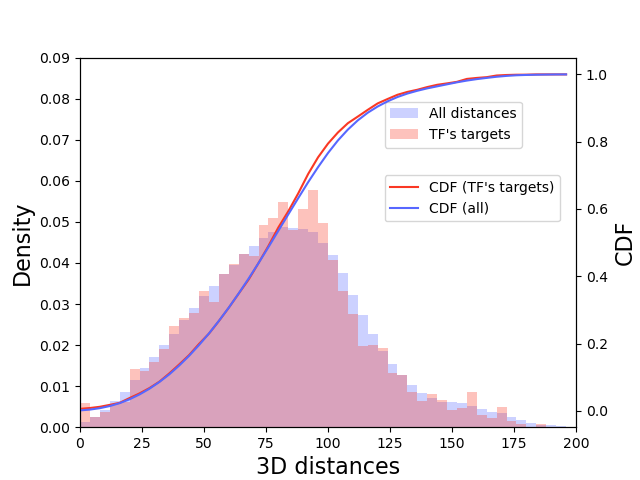

Supplement: Supplementary file 4 — Additional file 4. ZIP file with graphical representations associated to each transcriptional module: (link from Zenodo repository: https://zenodo.org/record/5841177/files/supplementary-data-file-S4.zip?download=1). [file 13104_2022_5940_MOESM4_ESM.zip › 3D_distances_distribution/ASH1_111_targets.csv.png]

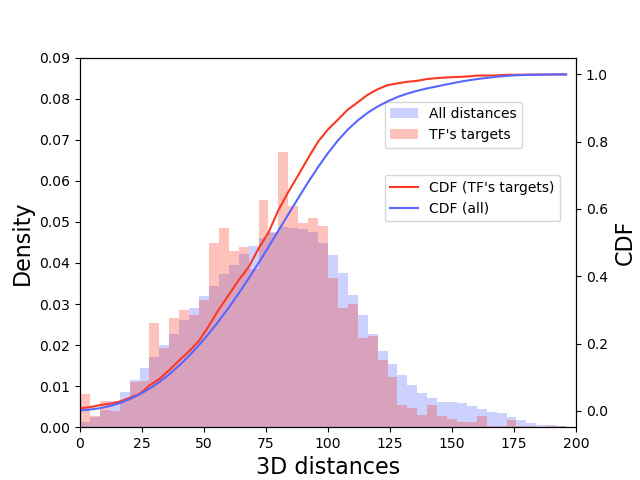

Supplement: Supplementary file 4 — Additional file 4. ZIP file with graphical representations associated to each transcriptional module: (link from Zenodo repository: https://zenodo.org/record/5841177/files/supplementary-data-file-S4.zip?download=1). [file 13104_2022_5940_MOESM4_ESM.zip › 3D_distances_distribution/CST6_78_targets.csv.png]

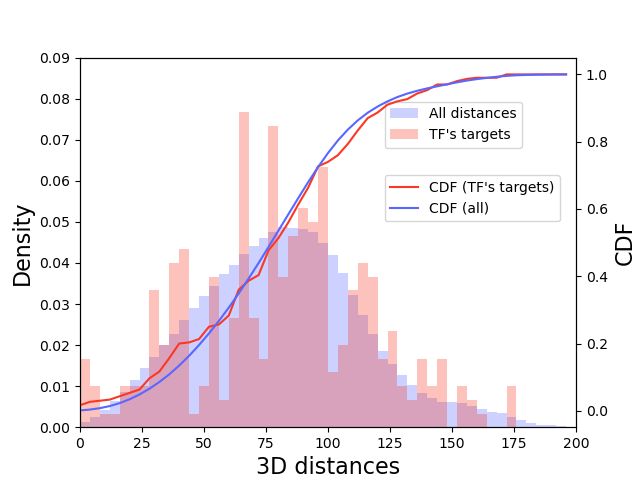

Supplement: Supplementary file 4 — Additional file 4. ZIP file with graphical representations associated to each transcriptional module: (link from Zenodo repository: https://zenodo.org/record/5841177/files/supplementary-data-file-S4.zip?download=1). [file 13104_2022_5940_MOESM4_ESM.zip › 3D_distances_distribution/ARR1_25_targets.csv.png]

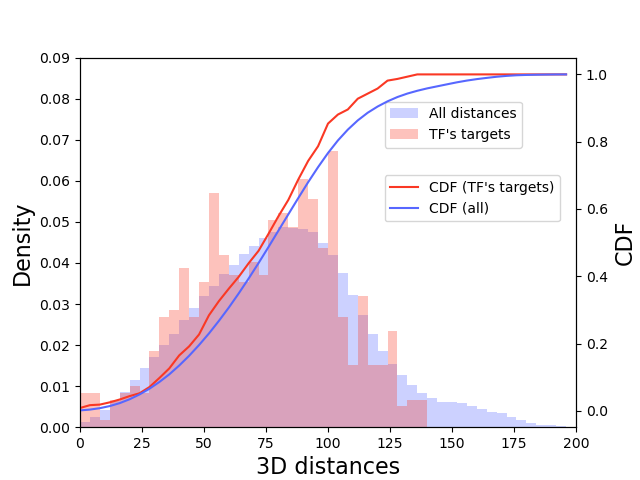

Supplement: Supplementary file 4 — Additional file 4. ZIP file with graphical representations associated to each transcriptional module: (link from Zenodo repository: https://zenodo.org/record/5841177/files/supplementary-data-file-S4.zip?download=1). [file 13104_2022_5940_MOESM4_ESM.zip › 3D_distances_distribution/RSF2_35_targets.csv.png]

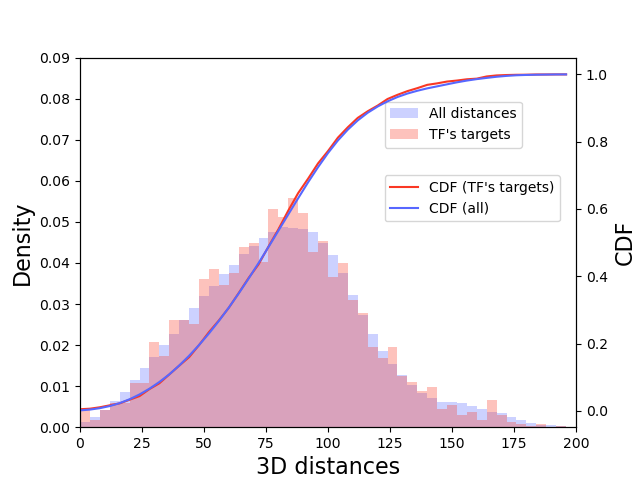

Supplement: Supplementary file 4 — Additional file 4. ZIP file with graphical representations associated to each transcriptional module: (link from Zenodo repository: https://zenodo.org/record/5841177/files/supplementary-data-file-S4.zip?download=1). [file 13104_2022_5940_MOESM4_ESM.zip › 3D_distances_distribution/RTG1_93_targets.csv.png]

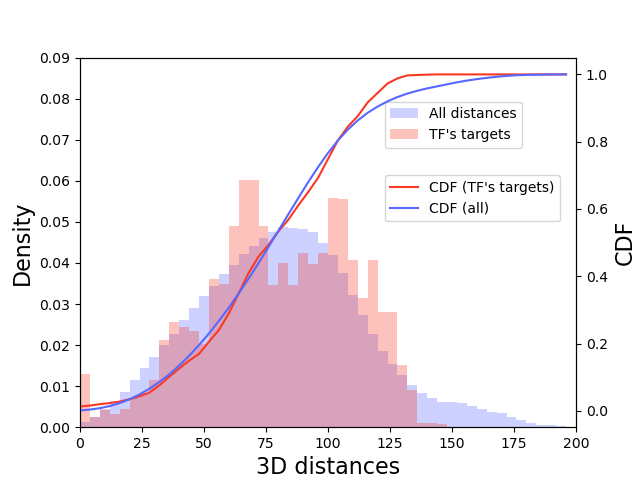

Supplement: Supplementary file 4 — Additional file 4. ZIP file with graphical representations associated to each transcriptional module: (link from Zenodo repository: https://zenodo.org/record/5841177/files/supplementary-data-file-S4.zip?download=1). [file 13104_2022_5940_MOESM4_ESM.zip › 3D_distances_distribution/RGM1_75_targets.csv.png]

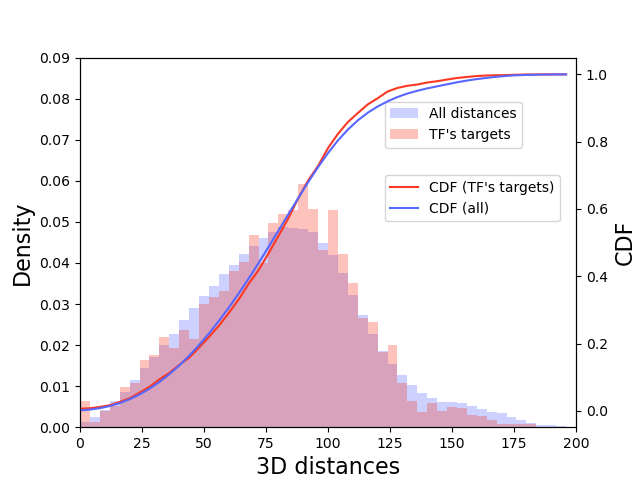

Supplement: Supplementary file 4 — Additional file 4. ZIP file with graphical representations associated to each transcriptional module: (link from Zenodo repository: https://zenodo.org/record/5841177/files/supplementary-data-file-S4.zip?download=1). [file 13104_2022_5940_MOESM4_ESM.zip › 3D_distances_distribution/MIG1_109_targets.csv.png]

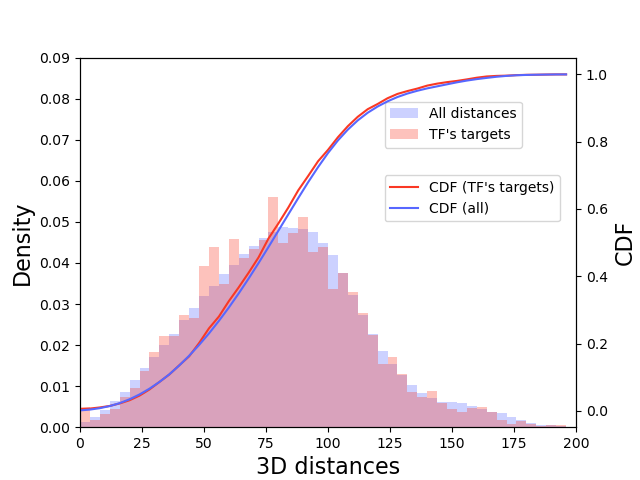

Supplement: Supplementary file 4 — Additional file 4. ZIP file with graphical representations associated to each transcriptional module: (link from Zenodo repository: https://zenodo.org/record/5841177/files/supplementary-data-file-S4.zip?download=1). [file 13104_2022_5940_MOESM4_ESM.zip › 3D_distances_distribution/MAL33_136_targets.csv.png]

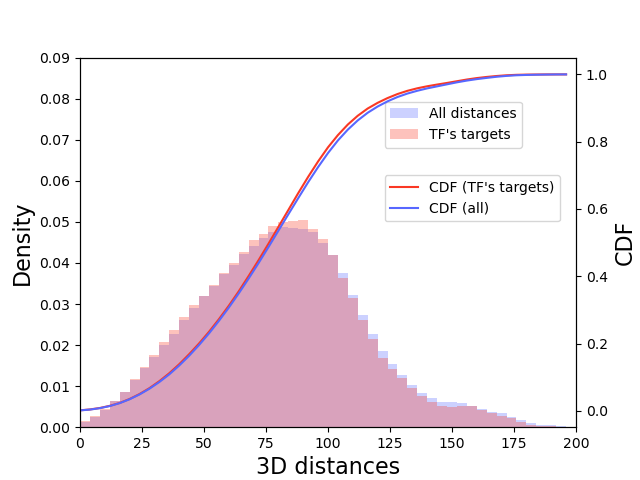

Supplement: Supplementary file 4 — Additional file 4. ZIP file with graphical representations associated to each transcriptional module: (link from Zenodo repository: https://zenodo.org/record/5841177/files/supplementary-data-file-S4.zip?download=1). [file 13104_2022_5940_MOESM4_ESM.zip › 3D_distances_distribution/NDT80_1755_targets.csv.png]

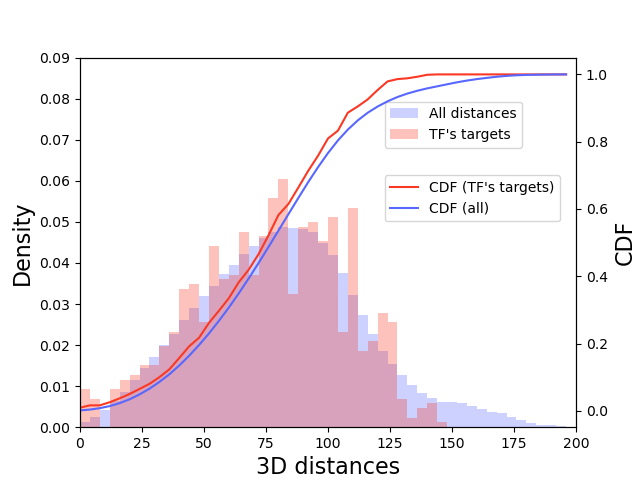

Supplement: Supplementary file 4 — Additional file 4. ZIP file with graphical representations associated to each transcriptional module: (link from Zenodo repository: https://zenodo.org/record/5841177/files/supplementary-data-file-S4.zip?download=1). [file 13104_2022_5940_MOESM4_ESM.zip › 3D_distances_distribution/HMS2_42_targets.csv.png]

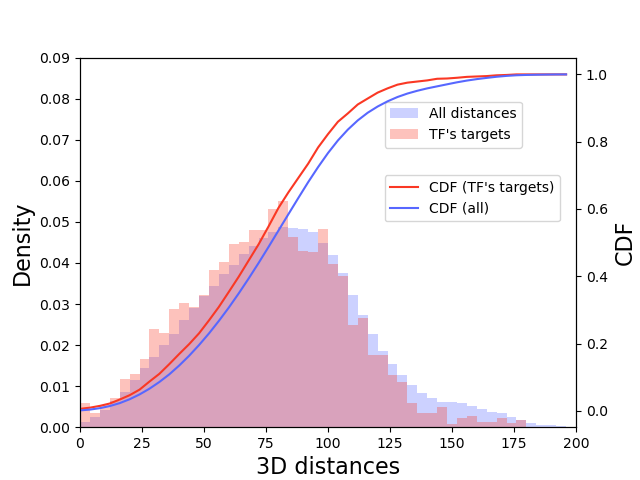

Supplement: Supplementary file 4 — Additional file 4. ZIP file with graphical representations associated to each transcriptional module: (link from Zenodo repository: https://zenodo.org/record/5841177/files/supplementary-data-file-S4.zip?download=1). [file 13104_2022_5940_MOESM4_ESM.zip › 3D_distances_distribution/STP2_110_targets.csv.png]

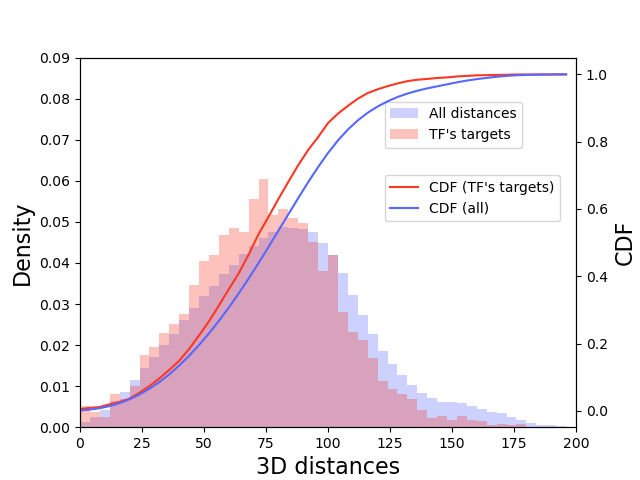

Supplement: Supplementary file 4 — Additional file 4. ZIP file with graphical representations associated to each transcriptional module: (link from Zenodo repository: https://zenodo.org/record/5841177/files/supplementary-data-file-S4.zip?download=1). [file 13104_2022_5940_MOESM4_ESM.zip › 3D_distances_distribution/PHO2_134_targets.csv.png]

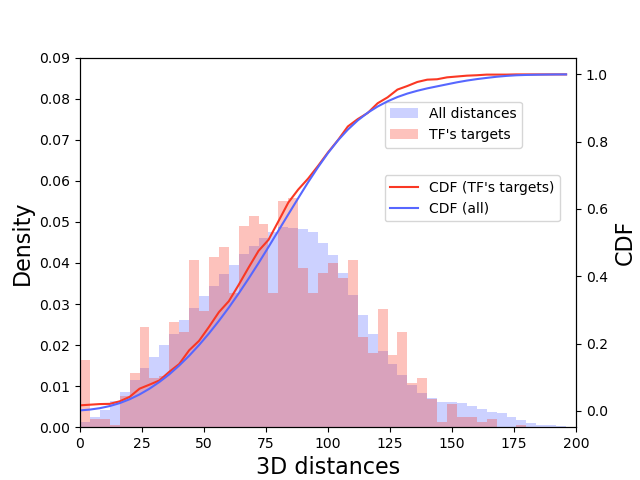

Supplement: Supplementary file 4 — Additional file 4. ZIP file with graphical representations associated to each transcriptional module: (link from Zenodo repository: https://zenodo.org/record/5841177/files/supplementary-data-file-S4.zip?download=1). [file 13104_2022_5940_MOESM4_ESM.zip › 3D_distances_distribution/GZF3_57_targets.csv.png]

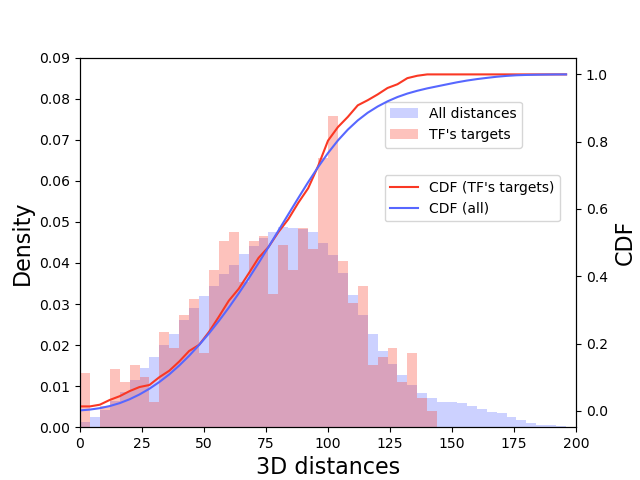

Supplement: Supplementary file 4 — Additional file 4. ZIP file with graphical representations associated to each transcriptional module: (link from Zenodo repository: https://zenodo.org/record/5841177/files/supplementary-data-file-S4.zip?download=1). [file 13104_2022_5940_MOESM4_ESM.zip › 3D_distances_distribution/LEU3_45_targets.csv.png]

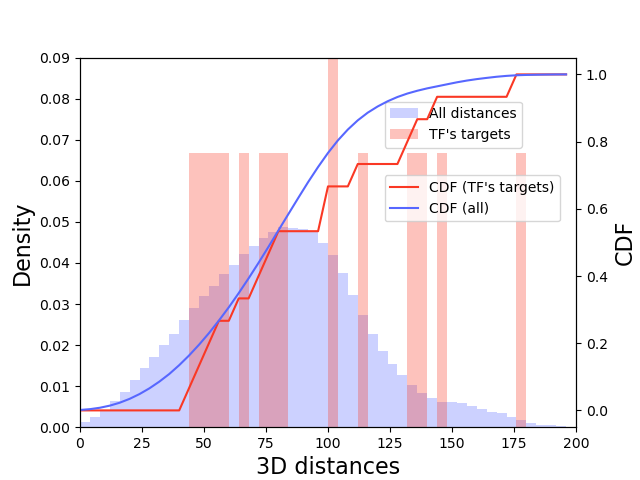

Supplement: Supplementary file 4 — Additional file 4. ZIP file with graphical representations associated to each transcriptional module: (link from Zenodo repository: https://zenodo.org/record/5841177/files/supplementary-data-file-S4.zip?download=1). [file 13104_2022_5940_MOESM4_ESM.zip › 3D_distances_distribution/WTM1_6_targets.csv.png]

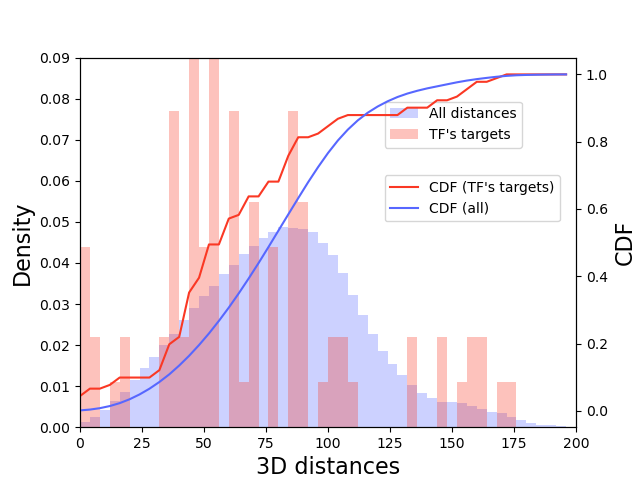

Supplement: Supplementary file 4 — Additional file 4. ZIP file with graphical representations associated to each transcriptional module: (link from Zenodo repository: https://zenodo.org/record/5841177/files/supplementary-data-file-S4.zip?download=1). [file 13104_2022_5940_MOESM4_ESM.zip › 3D_distances_distribution/COM2_14_targets.csv.png]

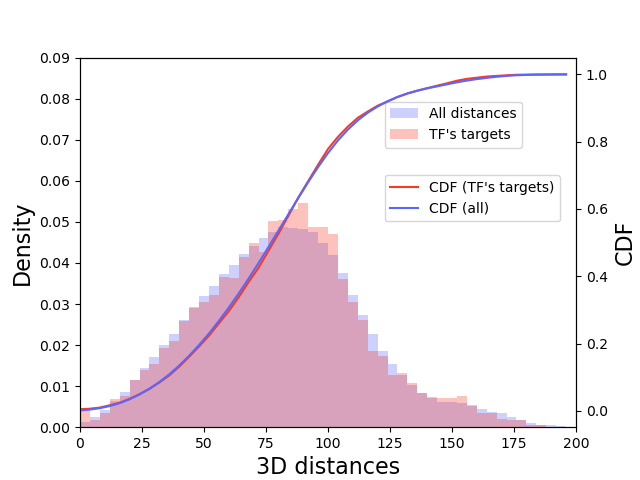

Supplement: Supplementary file 4 — Additional file 4. ZIP file with graphical representations associated to each transcriptional module: (link from Zenodo repository: https://zenodo.org/record/5841177/files/supplementary-data-file-S4.zip?download=1). [file 13104_2022_5940_MOESM4_ESM.zip › 3D_distances_distribution/IFH1_300_targets.csv.png]

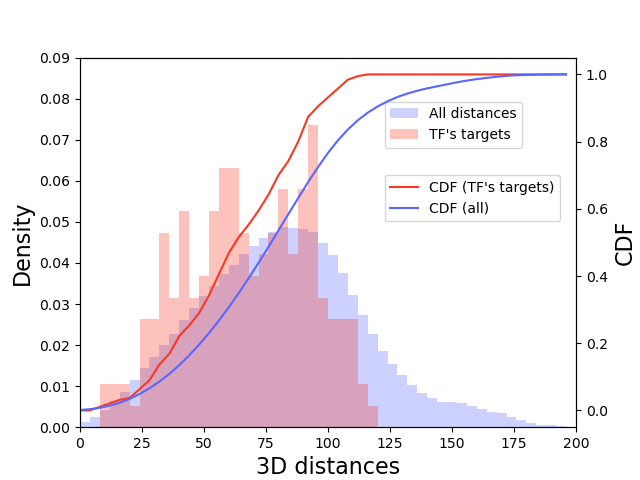

Supplement: Supplementary file 4 — Additional file 4. ZIP file with graphical representations associated to each transcriptional module: (link from Zenodo repository: https://zenodo.org/record/5841177/files/supplementary-data-file-S4.zip?download=1). [file 13104_2022_5940_MOESM4_ESM.zip › 3D_distances_distribution/GSM1_20_targets.csv.png]

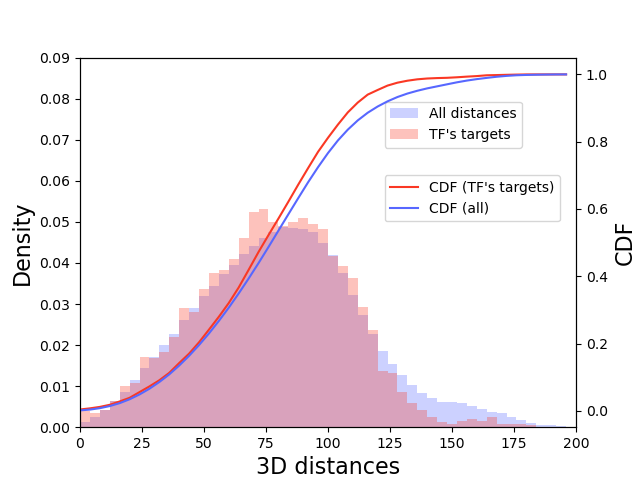

Supplement: Supplementary file 4 — Additional file 4. ZIP file with graphical representations associated to each transcriptional module: (link from Zenodo repository: https://zenodo.org/record/5841177/files/supplementary-data-file-S4.zip?download=1). [file 13104_2022_5940_MOESM4_ESM.zip › 3D_distances_distribution/STB5_214_targets.csv.png]

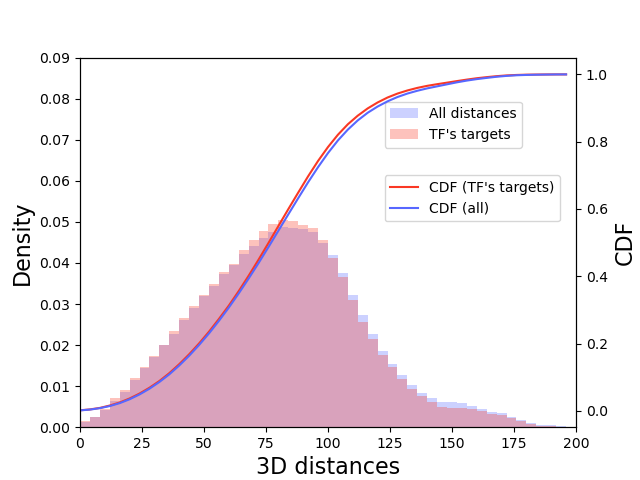

Supplement: Supplementary file 4 — Additional file 4. ZIP file with graphical representations associated to each transcriptional module: (link from Zenodo repository: https://zenodo.org/record/5841177/files/supplementary-data-file-S4.zip?download=1). [file 13104_2022_5940_MOESM4_ESM.zip › 3D_distances_distribution/FKH1_2263_targets.csv.png]

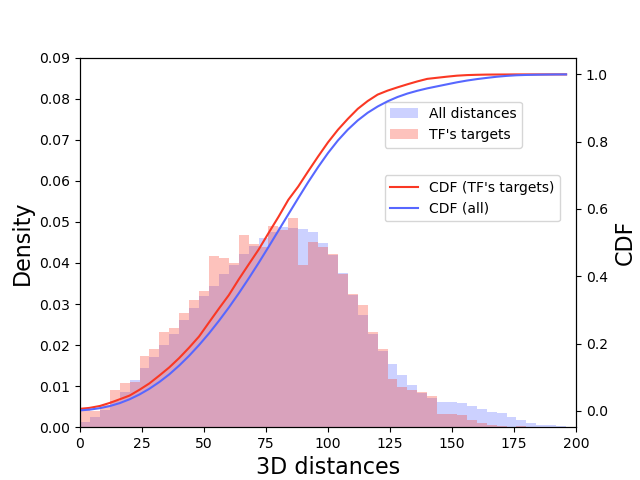

Supplement: Supplementary file 4 — Additional file 4. ZIP file with graphical representations associated to each transcriptional module: (link from Zenodo repository: https://zenodo.org/record/5841177/files/supplementary-data-file-S4.zip?download=1). [file 13104_2022_5940_MOESM4_ESM.zip › 3D_distances_distribution/RPN4_194_targets.csv.png]

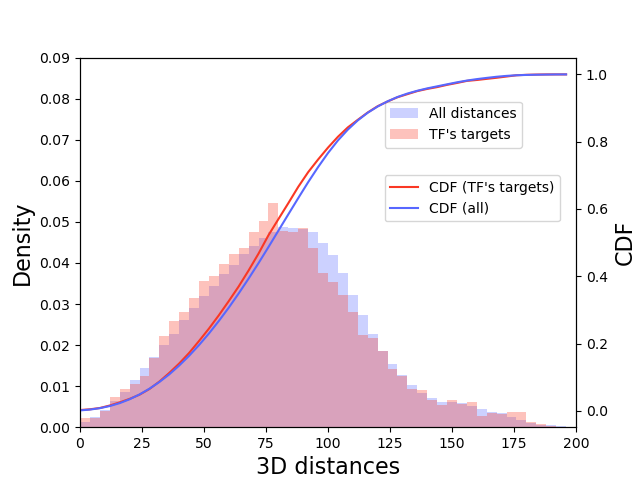

Supplement: Supplementary file 4 — Additional file 4. ZIP file with graphical representations associated to each transcriptional module: (link from Zenodo repository: https://zenodo.org/record/5841177/files/supplementary-data-file-S4.zip?download=1). [file 13104_2022_5940_MOESM4_ESM.zip › 3D_distances_distribution/CAD1_213_targets.csv.png]

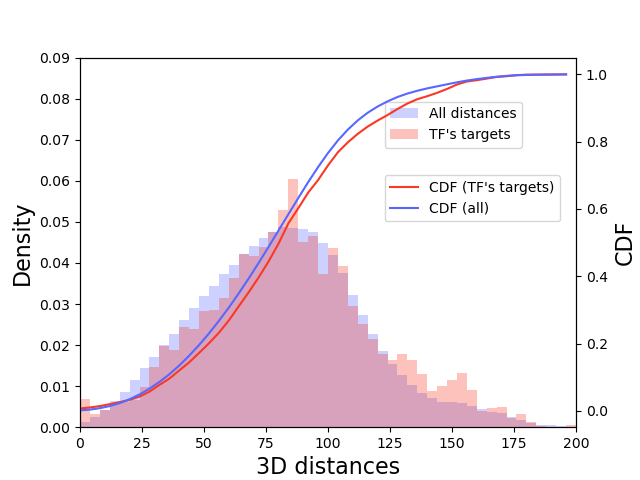

Supplement: Supplementary file 4 — Additional file 4. ZIP file with graphical representations associated to each transcriptional module: (link from Zenodo repository: https://zenodo.org/record/5841177/files/supplementary-data-file-S4.zip?download=1). [file 13104_2022_5940_MOESM4_ESM.zip › 3D_distances_distribution/RLM1_137_targets.csv.png]

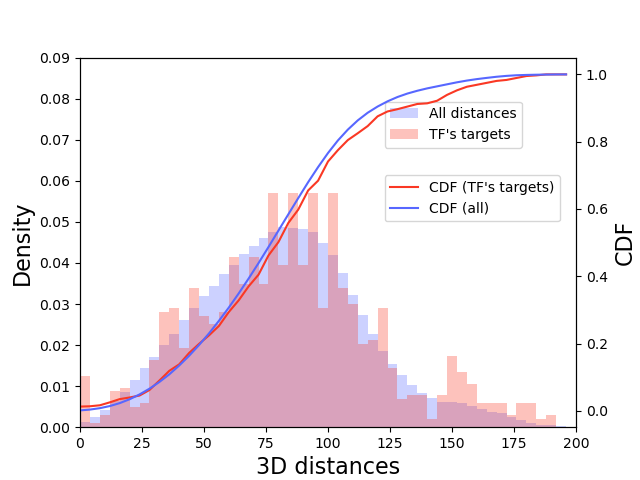

Supplement: Supplementary file 4 — Additional file 4. ZIP file with graphical representations associated to each transcriptional module: (link from Zenodo repository: https://zenodo.org/record/5841177/files/supplementary-data-file-S4.zip?download=1). [file 13104_2022_5940_MOESM4_ESM.zip › 3D_distances_distribution/MET28_46_targets.csv.png]

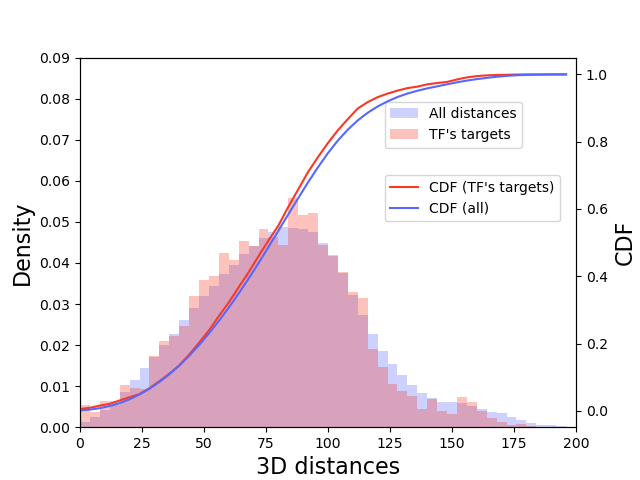

Supplement: Supplementary file 4 — Additional file 4. ZIP file with graphical representations associated to each transcriptional module: (link from Zenodo repository: https://zenodo.org/record/5841177/files/supplementary-data-file-S4.zip?download=1). [file 13104_2022_5940_MOESM4_ESM.zip › 3D_distances_distribution/STP1_144_targets.csv.png]

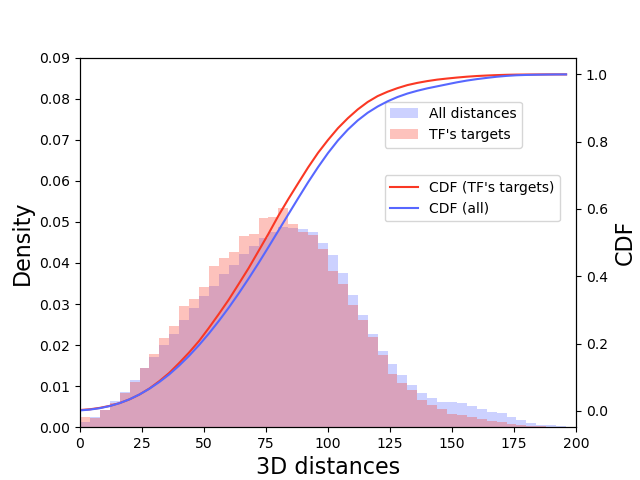

Supplement: Supplementary file 4 — Additional file 4. ZIP file with graphical representations associated to each transcriptional module: (link from Zenodo repository: https://zenodo.org/record/5841177/files/supplementary-data-file-S4.zip?download=1). [file 13104_2022_5940_MOESM4_ESM.zip › 3D_distances_distribution/TEC1_522_targets.csv.png]

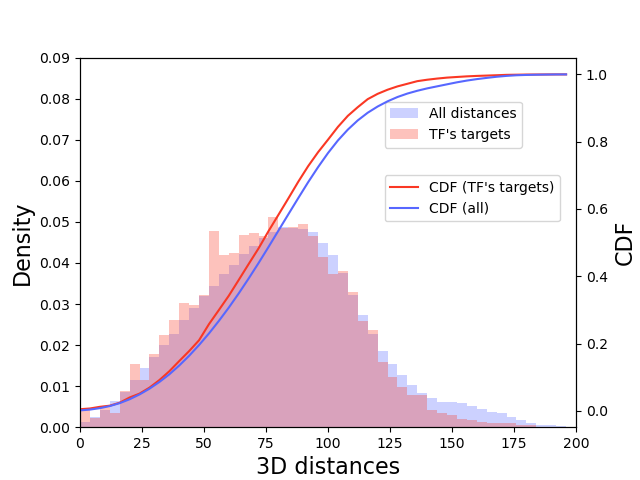

Supplement: Supplementary file 4 — Additional file 4. ZIP file with graphical representations associated to each transcriptional module: (link from Zenodo repository: https://zenodo.org/record/5841177/files/supplementary-data-file-S4.zip?download=1). [file 13104_2022_5940_MOESM4_ESM.zip › 3D_distances_distribution/ROX1_200_targets.csv.png]

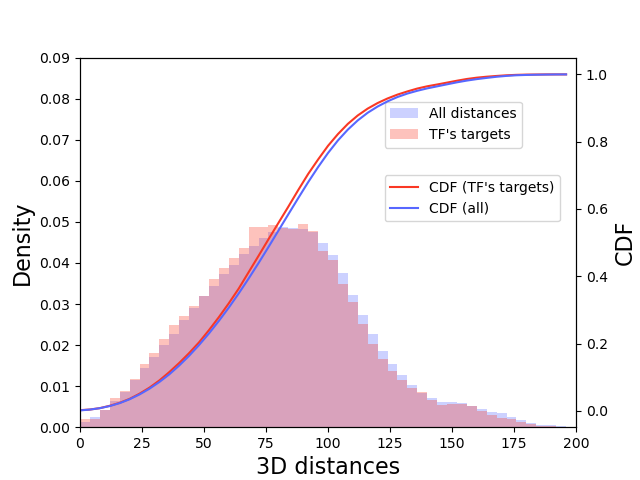

Supplement: Supplementary file 4 — Additional file 4. ZIP file with graphical representations associated to each transcriptional module: (link from Zenodo repository: https://zenodo.org/record/5841177/files/supplementary-data-file-S4.zip?download=1). [file 13104_2022_5940_MOESM4_ESM.zip › 3D_distances_distribution/SKO1_592_targets.csv.png]

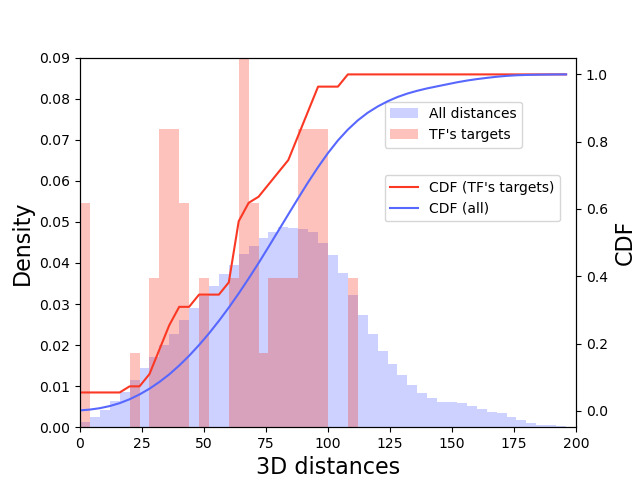

Supplement: Supplementary file 4 — Additional file 4. ZIP file with graphical representations associated to each transcriptional module: (link from Zenodo repository: https://zenodo.org/record/5841177/files/supplementary-data-file-S4.zip?download=1). [file 13104_2022_5940_MOESM4_ESM.zip › 3D_distances_distribution/STP4_11_targets.csv.png]

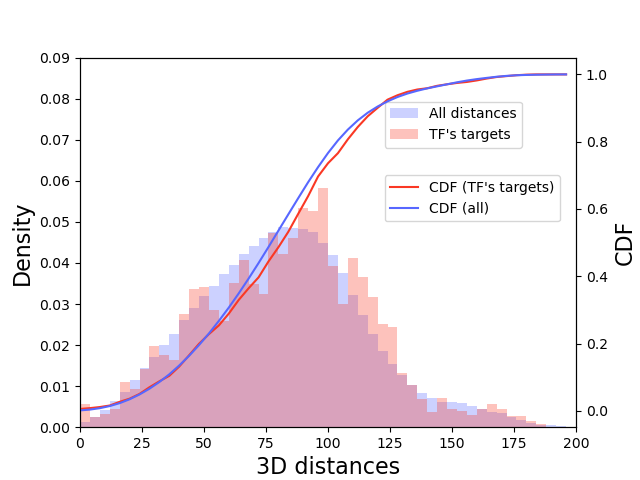

Supplement: Supplementary file 4 — Additional file 4. ZIP file with graphical representations associated to each transcriptional module: (link from Zenodo repository: https://zenodo.org/record/5841177/files/supplementary-data-file-S4.zip?download=1). [file 13104_2022_5940_MOESM4_ESM.zip › 3D_distances_distribution/FZF1_87_targets.csv.png]

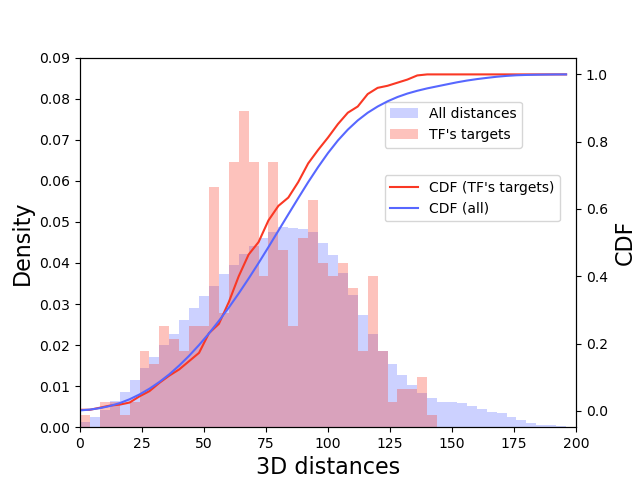

Supplement: Supplementary file 4 — Additional file 4. ZIP file with graphical representations associated to each transcriptional module: (link from Zenodo repository: https://zenodo.org/record/5841177/files/supplementary-data-file-S4.zip?download=1). [file 13104_2022_5940_MOESM4_ESM.zip › 3D_distances_distribution/MGA2_26_targets.csv.png]

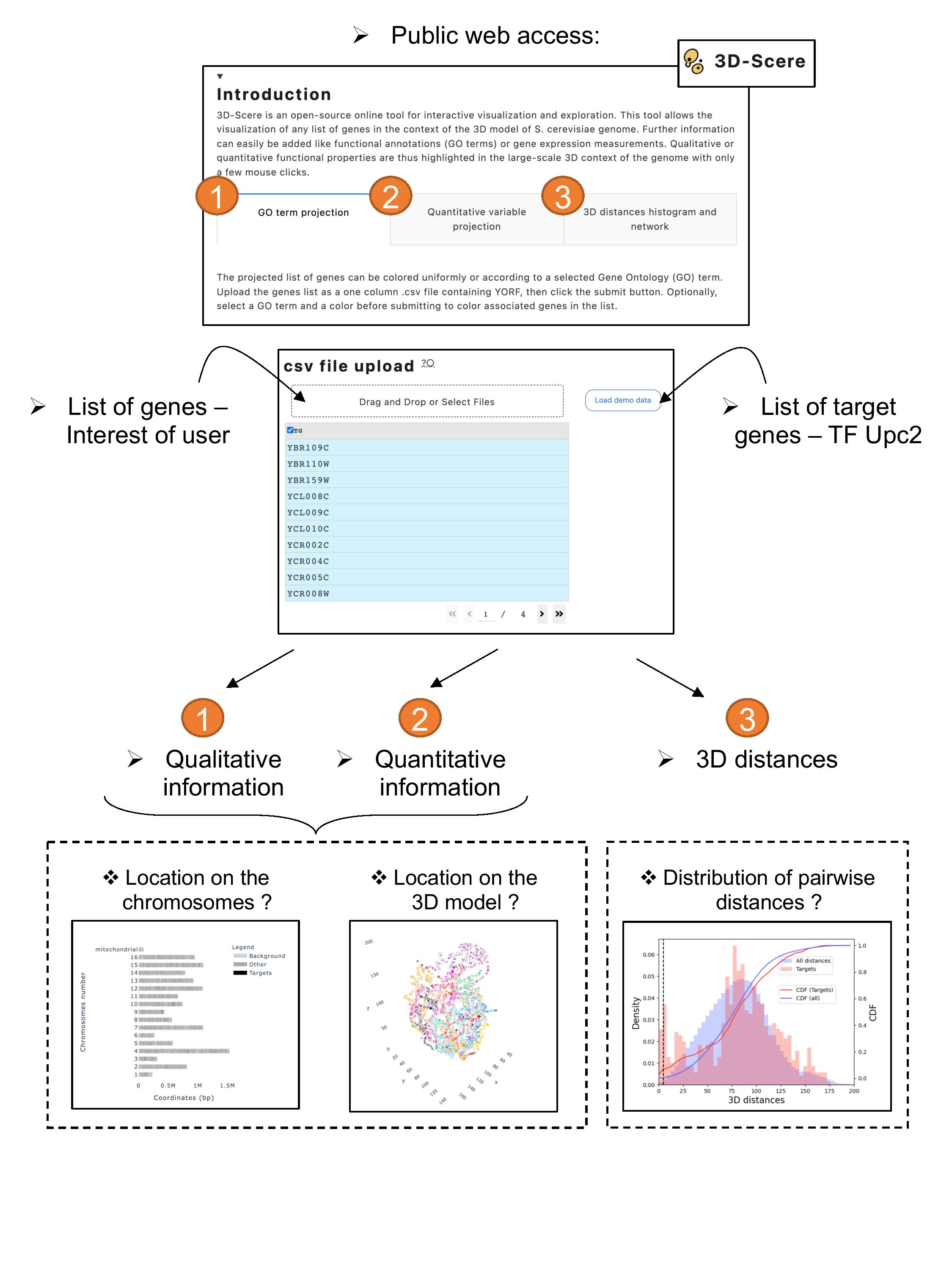

Supplement: Supplementary file 5 — Additional file 5. General overview of the 3d-Scere tool. A public web acces is available at https://3d-scere.ijm.fr. Three different uses are proposed to users: (1) «GO term projection», (2) Quantitative variable projection and (3) 3D distances histogram and network. Each access starts with the upload of a list of genes of interest for the user. Note that the list of Upc2 targets can be loaded as a «demo data». From the list of genes, users can manipulate either qualitative information (Access 1) or quantitative information (Access 2) and obtain graphics showing location on the chromosome or location on the 3D model of S. cerevisiae genome. Distribution of pairwise distances between genes is obtain with the Access 3. [file 13104_2022_5940_MOESM5_ESM.jpg]
