# Supplementary material for: Additional insights into the organization of transcriptional regulatory modules based on a 3D model of the Saccharomyces cerevisiae genome
Source: BMC Res Notes. 2022 Feb 19;15:67. doi: 10.1186/s13104-022-05940-5 (PMC8858486; doi:10.1186/s13104-022-05940-5)
Supplement: Supplementary file 6 — Additional file 6. New insights into the transcriptional module related to Upc2 transcription factor. [file 13104_2022_5940_MOESM6_ESM.pdf]

Supplementary file S6

New insights into the transcriptional module related to Upc2 transcription factor

In the main text, we present a systematic analysis of all transcriptional modules in the model yeast *Saccharomyces cerevisiae*. It allowed us to observe an interesting situation, regarding the Upc2 transcriptional module. In the *S. cerevisiae*, Upc2 is a transcription factor that belongs to the family of $Zn(II)_2Cys_6$ regulatory proteins [1]. It regulates the biosynthesis of ergosterols, which are key components of the yeast cytoplasmic membrane, modulating membrane fluidity, stability, and permeability. Notably, ergosterols are the target of the most used antifungals [2]. Better understanding of the underlying cellular processes, by which yeast cells control ergosterol production, thus has interesting applications (study of drug resistance mechanisms for instance [3]).

In our data, the transcriptional module of Upc2 comprises 38 target genes, located on 14 different chromosomes (**Table 1**, below). As expected, these genes are involved in lipid metabolic process (GO:0006629: YBR109C, YBR110W, YBR159W, YDR372C, YDR373W, YMR202W, YOR365C) or response to chemical (GO:0042221: YCR008W, YDR179C, YDR372C, YJR100C, YLR097C). This is consistent with the known function of Upc2 transcription factor, *i.e.* sterol homeostasis and resistance to azole drugs [1]. The distribution of the pairwise Euclidean distances between the 38 target genes (based on the 3D model of *S. cerevisiae* genome, see main text for more details) is shown **Figure 1** (below). It was obtained applying the 3d-Scere tool available online (https://3d-scere.ijm.fr). It is interesting to observe that, inside the Upf2 transcriptional module, a subset of 14 genes appears to be co-localized (see the red arrow, **Figure 1**). Also, it is worth considering that they are located on different chromosomes: 10 genes are on chromosome III, 1 gene is on chromosome VI and 3 genes are on chromosome X (see **Table 1** for more details). Our tool thus makes it possible to show a strong association between a subset of genes that is not visible considering the genomic location only.

Additional research is of course necessary to understand the functional interest of such a grouping of genes in the nucleus, but this example illustrates the interest to take into account the 3D model of the genome of *S. cerevisiae*, offering new insights for the study of transcriptional regulations. Using few mouse clicks, it reveals original features distinguishing target genes within transcriptional modules.

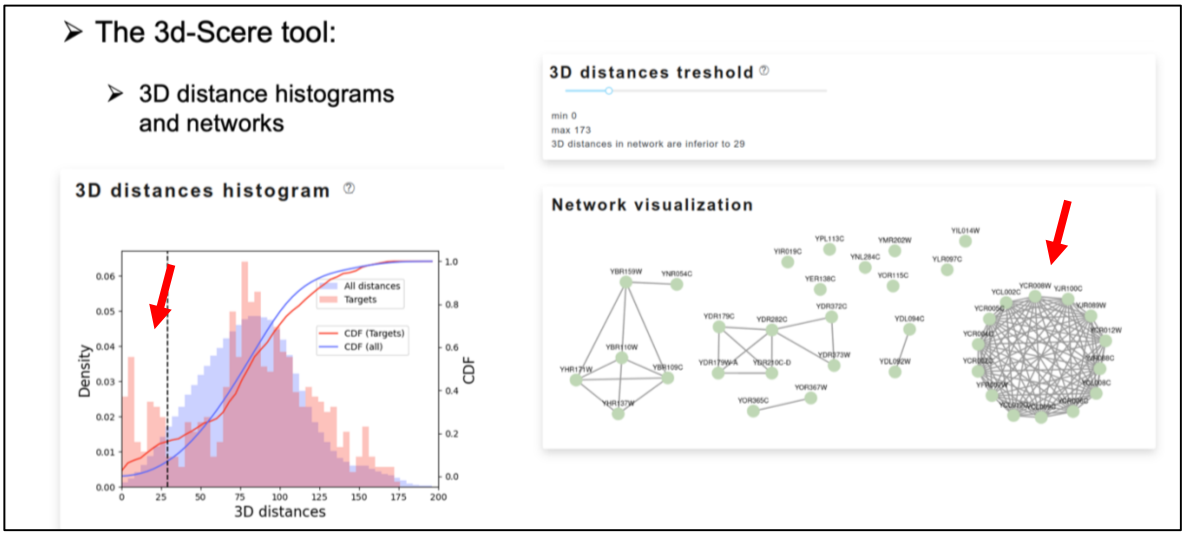

Figure 1: Screenshots of output graphs obtained with the 3d-Scere tool. In this example, the list of the 38 target genes for the Upc2 transcription factor was submitted to the tool (available online https://3d-scere.ijm.fr). Histogram of the 3D distances between all pairwise target genes is shown on the left (pink color) and is compared to the overall distribution of 3D distance (all genes in S. cerevisiae*, in blue). Network visualization of the Upc2 transcriptional module is shown on the right. Target genes are represented by nodes and edges connect them if their 3D distance is lower than a fixed threshold (value 29 here). Red arrows point a subset of target genes for which atypical low distances are observed (see main text for discussion regarding this observation).*

ORF Name	Gene Name	Description	Chromosome location
YBR109C	CMD1	Calmodulin; Ca++ binding protein that regulates Ca++ independent processes (mitosis, bud growth, actin organization, endocytosis, etc.) and Ca++ dependent processes (stress-activated pathways), targets include Nuf1p, Myo2p and calcineurin	II
YBR110W	ALG1	Mannosyltransferase, involved in asparagine-linked glycosylation in the endoplasmic reticulum (ER); essential for viability, mutation is functionally complemented by human ortholog	II
YBR159W	IFA38	Microsomal beta-keto-reductase; contains oleate response element (ORE) sequence in the promoter region; mutants exhibit reduced VLCFA synthesis, accumulate high levels of dihydrosphingosine, phytosphingosine and medium-chain ceramides	II
YCL002C	YCL002C	Putative protein of unknown function; YCL002C is not an essential gene	III
YCL008C	STP22	Component of the ESCRT-I complex, which is involved in ubiquitin-dependent sorting of proteins into the endosome; homologous to the mouse and human Tsg101 tumor susceptibility gene; mutants exhibit a Class E Vps phenotype	III
YCL009C	ILV6	Regulatory subunit of acetolactate synthase, which catalyzes the first step of branched-chain amino acid biosynthesis; enhances activity of the Ilv2p catalytic subunit, localizes to mitochondria	III
YCL010C	SGF29	Component of the HAT/Core module of the SAGA, SLIK, and ADA complexes; HAT/Core module also contains Gcn5p, Ngg1p, and Ada2p; binds methylated histone H3K4; involved in transcriptional regulation through SAGA recruitment to target promoters and H3 acetylation	III

YCR002C	CDC10	Component of the septin ring that is required for cytokinesis; septins recruit proteins to the mother-bud neck and can act as a barrier to diffusion at the membrane, and they comprise the 10 nm filaments seen with EM; required for the transition from a single to double septin ring	III
YCR004C	YCP4	Protein of unknown function, has sequence and structural similarity to flavodoxins; predicted to be palmitoylated; the authentic, non-tagged protein is detected in highly purified mitochondria in high-throughput studies	III
YCR005C	CIT2	Citrate synthase, catalyzes the condensation of acetyl coenzyme A and oxaloacetate to form citrate, peroxisomal isozyme involved in glyoxylate cycle; expression is controlled by Rtg1p and Rtg2p transcription factors	III
YCR006C	YCR006C	Dubious open reading frame unlikely to encode a protein, based on available experimental and comparative sequence data	III
YCR008W	SAT4	Ser/Thr protein kinase involved in salt tolerance; funtions in regulation of Trk1p-Trk2p potassium transporter; partially redundant with Hal5p; has similarity to Npr1p	III
YCR012W	PGK1	3-phosphoglycerate kinase, catalyzes transfer of high-energy phosphoryl groups from the acyl phosphate of 1,3-bisphosphoglycerate to ADP to produce ATP; key enzyme in glycolysis and gluconeogenesis	III
YDL092W	SRP14	Signal recognition particle (SRP) subunit, interacts with the RNA component of SRP to form the Alu domain, which is the region of SRP responsible for arrest of nascent chain elongation during membrane targeting; homolog of mammalian SRP14	IV
YDL094C	YDL094C	Dubious open reading frame unlikely to encode a protein, based on available experimental and comparative sequence data; partially overlaps verfied gene PMT5/YDL093W; YDL094C is not essential	IV
YDR179C	CSN9	Subunit of the Cop9 signalosome, which is required for deneddylation, or removal of the ubiquitin-like protein Rub1p from Cdc53p (cullin); involved in adaptation to pheromone signaling	IV
YDR179W-A	NVJ3	Putative protein of unknown function	IV
YDR210C-D	YDR210C-D	Retrotransposon TYA Gag and TYB Pol genes; transcribed/translated as one unit; polyprotein is processed to make a nucleocapsid-like protein (Gag), reverse transcriptase (RT), protease (PR), and integrase (IN); similar to retroviral genes	IV
YDR282C	MRX10	Putative protein of unknown function	IV
YDR372C	VPS74	Protein required for Golgi localization of glycosyltransferases; binds the cytosolic domains of Golgi glycosyltransferases; binding to PtdIns4P required for Golgi targeting and function; tetramer formation required for function	IV

YDR373W	FRQ1	N-myristoylated calcium-binding protein that may have a role in intracellular signaling through its regulation of the phosphatidylinositol 4-kinase Pik1p; member of the recoverin/frequenin branch of the EF-hand superfamily	IV
YER138C	YER138C	Retrotransposon TYA Gag and TYB Pol genes; transcribed/translated as one unit; polyprotein is processed to make a nucleocapsid-like protein (Gag), reverse transcriptase (RT), protease (PR), and integrase (IN); similar to retroviral genes	V
YFR007W	YFH7	Putative kinase with similarity to the phosphoribulokinase/uridine kinase/bacterial pantothenate kinase (PRK/URK/PANK) subfamily of P-loop kinases	VI
YHR137W	ARO9	Aromatic aminotransferase II, catalyzes the first step of tryptophan, phenylalanine, and tyrosine catabolism	VIII
YHR171W	ATG7	Autophagy-related protein and dual specificity member of the E1 family of ubiquitin-activating enzymes; mediates the conjugation of Atg12p with Atg5p and Atg8p with phosphatidylethanolamine, required steps in autophagosome formation	VIII
YIL014W	MNT3	Alpha-1,3-mannosyltransferase, adds the fourth and fifth alpha-1,3-linked mannose residues to O-linked glycans during protein O-glycosylation	IX
YIR019C	FLO11	GPI-anchored cell surface glycoprotein (flocculin) required for pseudohyphal formation, invasive growth, flocculation, and biofilms; transcriptionally regulated by the MAPK pathway (via Ste12p and Tec1p) and the cAMP pathway (via Flo8p)	IX
YJR088C	EMC2	Member of a transmembrane complex required for efficient folding of proteins in the ER; null mutant displays induction of the unfolded protein response	X
YJR089W	BIR1	Subunit of chromosomal passenger complex (CPC; Ipl1p-Sli15p-Bir1p-Nbl1p), which regulates chromosome segregation; required for chromosome bi-orientation and for spindle assembly checkpoint activation upon reduced sister kinetochore tension	X
YJR100C	AIM25	Putative protein of unknown function; non-tagged protein is detected in purified mitochondria in high-throughput studies; similar to murine NOR1; null mutant is viable and displays elevated frequency of mitochondrial genome loss	X
YLR097C	HRT3	Putative SCF-ubiquitin ligase F-box protein, based on both genetic and physical interactions and sequence similarity; identified in association with Cdc53p, Skp1p and Ubi4 in large and small-scale studies	XII
YMR202W	ERG2	C-8 sterol isomerase, catalyzes the isomerization of the delta-8 double bond to the delta-7 position at an intermediate step in ergosterol biosynthesis	XIII
YNL284C	MRPL10	Mitochondrial ribosomal protein of the large subunit; appears as two protein spots (YmL10 and YmL18) on two-dimensional SDS gels	XIV

YNR054C	ESF2	Essential nucleolar protein involved in pre-18S rRNA processing; binds to RNA and stimulates ATPase activity of Dbp8; involved in assembly of the small subunit (SSU) processome	XIV
YOR115C	TRS33	One of 10 subunits of the transport protein particle (TRAPP) complex of the cis-Golgi which mediates vesicle docking and fusion; involved in endoplasmic reticulum (ER) to Golgi membrane traffic	XV
YOR365C	YOR365C	Putative protein of unknown function; YOR365C is not an essential protein	XV
YOR367W	SCP1	Component of yeast cortical actin cytoskeleton, binds and cross links actin filaments; originally identified by its homology to calponin (contains a calponin-like repeat) but the Scp1p domain structure is more similar to transgelin	XV
YPL113C	YPL113C	Glyoxylate reductase; acts on glyoxylate and hydroxypyruvate substrates; YPL113C is not an essential gene	XVI

Table 1: List of target genes in the Upc2 transcriptional module. The transcriptional module was retrieved from the YEASTRACT database as described in the main text. Gene name, gene description and location on chromosomes were also obtained from YEASTRACT using the tool http://www.yeastract.com/formorftogene.php. Genes which are highlighted in color are those for which low 3D distance values were observed in our analyses (see Figure 1, red arrow).

References

[1] Vik et J. Rine, « Upc2p and Ecm22p, dual regulators of sterol biosynthesis in Saccharomyces cerevisiae », *Mol. Cell. Biol.*, vol. 21, nº 19, p. 6395-6405, oct. 2001, doi: 10.1128/MCB.21.19.6395-6405.2001.

[2] T. Jordá et S. Puig, « Regulation of Ergosterol Biosynthesis in Saccharomyces cerevisiae », *Genes*, vol. 11, nº 7, p. E795, juill. 2020, doi: 10.3390/genes11070795.

[3] P. Yibmantasiri, P. W. Bircham, D. R. Maass, D. S. Bellows, et P. H. Atkinson, « Networks of genes modulating the pleiotropic drug response in Saccharomyces cerevisiae », *Mol. Biosyst.*, vol. 10, nº 1, p. 128-137, janv. 2014, doi: 10.1039/c3mb70351g.
